# Presynaptic filopodia form kinapses and promote convective membrane flow to sustain synchronous neurotransmitter release

**DOI:** 10.1126/sciadv.aei2661

**Published:** 2026-10-28

**Authors:** Akanksha Jain, Rico Dong, Jana Kroll, Jack F. Webster, Jon Moss, Arthur Bikbaev, Sila K. Ultanir, Alfredo Gonzalez-Sulser, Christian Rosenmund, Martin Heine, Jian Liu, Michael A. Cousin, Daniela Ivanova

**Affiliations:** ^1^ Institute for Neuroscience and Cardiovascular Research, Hugh Robson Building, George Square, University of Edinburgh, Edinburgh, Scotland, UK.; ^2^ Muir Maxwell Epilepsy Centre, Hugh Robson Building, George Square, University of Edinburgh, Edinburgh, Scotland, UK.; ^3^ Simons Initiative for the Developing Brain, Hugh Robson Building, George Square, University of Edinburgh, Edinburgh, Scotland, UK.; ^4^ Department of Cell Biology, School of Medicine, Johns Hopkins University, Baltimore, MD, USA.; ^5^ Center for Cell Dynamics, School of Medicine, Johns Hopkins University, Baltimore, MD, USA.; ^6^ Charité-Universitätsmedizin Berlin, Corporate Member of Freie Universität Berlin and Humboldt-Universität zu Berlin, Institute of Neurophysiology, Charitéplatz 1, Berlin, Germany.; ^7^ The Roslin Institute 3D Volume Electron Microscopy Capability, Proteomics and Metabolomics Facility, The Roslin Institute, Royal (Dick) School of Veterinary Studies, College of Medicine and Veterinary Medicine, University of Edinburgh, Edinburgh, Scotland, UK.; ^8^ Johannes Gutenberg University Mainz, Institute of Developmental Biology and Neurobiology, AG Functional Neurobiology, Biocenter 1, Hanns-Dieter Hüsch Weg 15, Mainz, Germany.; ^9^ Kinases and Brain Development Lab, The Francis Crick Institute, London, UK.

## Abstract

Synaptic plasticity is the principal mechanism by which neural circuits compute and adapt. Presynaptic plasticity is traditionally regarded as a transient adaptation of the synaptic vesicle fusion and recycling machinery, whereas the role of structural remodeling of synaptic boutons remains unclear. Here, we demonstrate a mechanical coupling between presynaptic structural remodeling and synaptic efficacy. We find that dynamic presynaptic filopodia emerge during active synaptic transmission and deform synaptic boutons while forming transient contacts with postsynaptic structures, which we term neuronal kinapses. Filopodia formation is activity dependent and occurs in intact circuits and human brain. Mechanistically, filopodial growth induces convective in-plane membrane flow toward the filopodium, accelerating release site clearance, a process associated with sustained release probability during repeated synaptic vesicle fusion. As a result, filopodia formation favors an increase in synchronous over asynchronous neurotransmitter release. These results indicate that presynaptic structural remodeling can alter the spatial organization of neurotransmission and, through the membrane flow it generates, modulate its temporal precision.

## INTRODUCTION

Structural and functional synaptic plasticity are intertwined. At the postsynaptic side, structural changes in dendritic spines are both consequences of neuronal activity and instigators that influence how dendrites integrate excitatory input by compartmentalizing biochemical and electrochemical signals (*1*, *2*).

Neurons regularly face sustained synaptic activity lasting seconds to minutes during normal behavior (*3*, *4*), a challenge further amplified under pathological conditions (*5*). This activity induces rapid and persistent enlargement of dendritic spines on minute timescales, a hallmark of long-term potentiation and memory formation (*1*, *6*, *7*). In contrast, we still lack direct evidence that presynaptic terminals undergo comparable structural remodeling during sustained activity.

Sustained synaptic transmission depends on the continuous recycling of neurotransmitter-filled synaptic vesicles (SVs) (*8*), a process that involves rapid membrane turnover at presynaptic boutons. Despite this constant flux, boutons are thought to maintain stable architecture over days to months (*9*, *10*). The few recognized forms of presynaptic structural plasticity include bouton formation and elimination or modest size fluctuations (*9*), processes that are modulated by neuronal activity and, in turn, adjust synaptic strength. This limited repertoire of presynaptic structural plasticity contrasts with the established role of slow, large-scale axonal expansion in circuit refinement during brain development and following periods of excessive synaptic drive (*11*, *12*). Whether this remodeling occurs at the level of individual boutons and whether it modifies synaptic function remain open questions.

Structural changes redistribute forces across the plasma membrane and cortical actin cytoskeleton, generating membrane tension gradients that polarize the distribution of structural and signaling molecules (*13*–*16*). Unlike passive diffusion, convective membrane flow physically displaces membrane-associated proteins from specific zones, concentrating or depleting molecules in ways that directly alter the biochemical reactions occurring there (*17*, *18*). At presynaptic boutons, where molecular crowding and rapid membrane turnover place particular demands on spatial organization, the local mechanical environment influences both SV exocytosis and endocytosis (*19*–*21*). However, current models of presynaptic plasticity lack a physiological mechanism linking the mechanical forces generated by structural remodeling to changes in presynaptic output.

Here, we show that transient sprouting is a widespread feature of neurotransmitter-releasing presynaptic boutons. This initial phase of axonal remodeling involves dynamically protruding and contracting filopodia. Filopodia formation is induced by neuronal activity and enables boutons to form transient, activity-dependent contacts with postsynaptic targets, which we term kinapses. Functional analyses and mathematical modeling demonstrate that filopodia formation is associated with a redistribution of vesicular membrane components toward filopodia, consistent with convective in-plane membrane flow. This flow is predicted to facilitate clearance of release sites during repeated SV fusion. In agreement, boutons bearing filopodia display higher release probability and a shift in neurotransmitter release toward synchronous over asynchronous modes during sustained activity. Together, our results support a mechanically driven interplay between structural and functional presynaptic plasticity that contributes to the spatial and temporal organization of neurotransmission.

## RESULTS

### Presynaptic filopodia are a ubiquitous structural response to neuronal activity

Activity-induced presynaptic structural plasticity has been hypothesized (*22*), but whether this remodeling occurs on the timescale of active neurotransmission and thereby influences neurotransmitter release remains unknown. To address this, we labeled axonal membranes in primary hippocampal cultures with glycosylphosphatidylinositol–enhanced green fluorescent protein (GPI-EGFP) while coexpressing synaptophysin-mOrange2 (SypmOr2), a pH-sensitive reporter of SV exo-endocytosis in which SV fusion is reported as an increase in fluorescence. High-resolution time-lapse imaging revealed that 44% of presynapses that were responsive to electrical field stimulation [600 action potentials (APs) at 40 Hz] formed elongated filopodial protrusions (defined here as presynaptic membrane extensions exceeding 200 nm in length) during stimulation (Fig. 1, A and B, and movie S1).

**Fig. 1.**
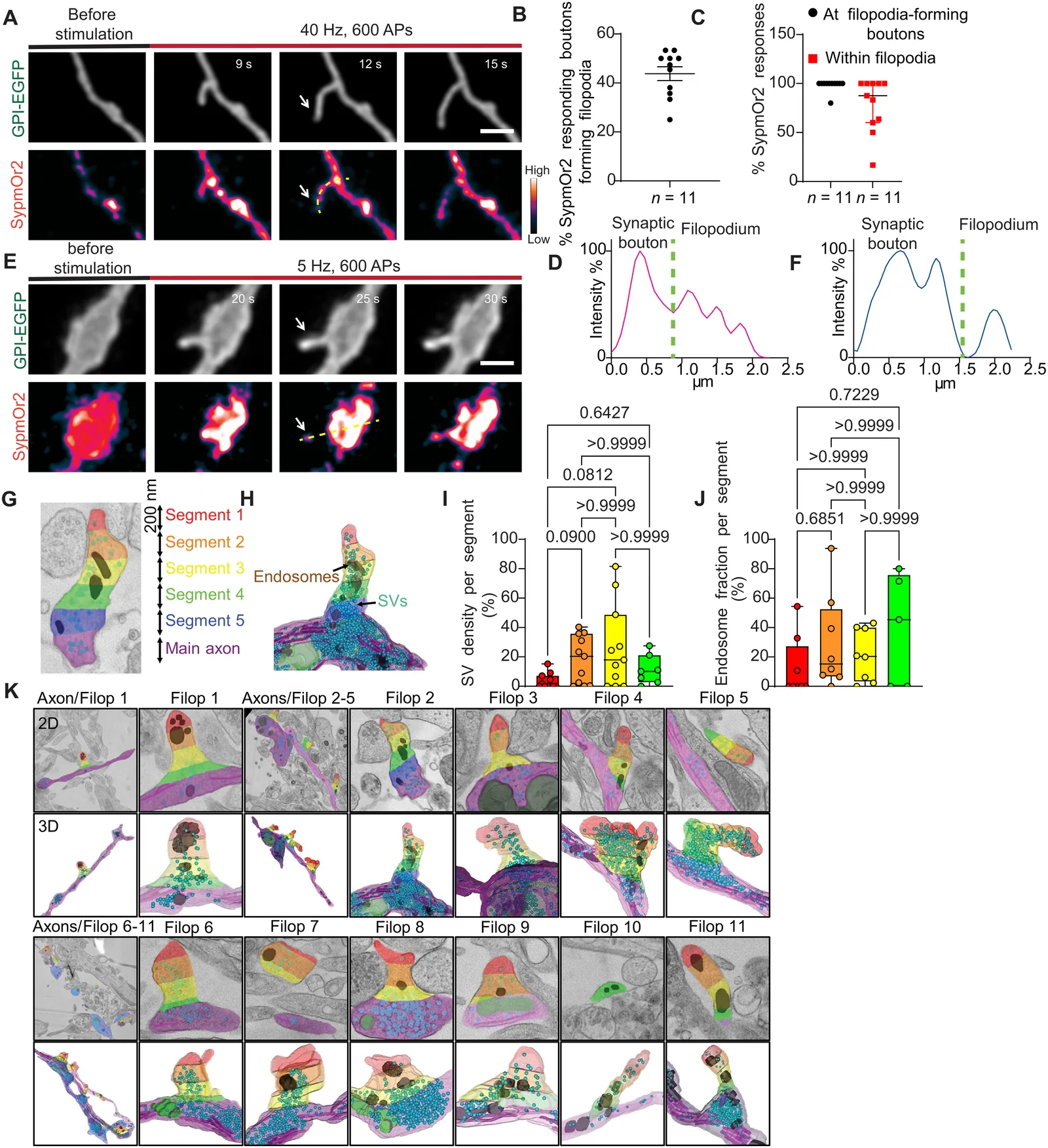
Filopodia form at presynaptic boutons during synaptic activity and show varied morphology and distributions of SVs and endosomes. (**A**) Time-lapse series of an axon coexpressing GPI-EGFP and SypmOr2 during stimulation with 600 APs (40 Hz). White arrow, filopodium. Scale bar, 1 μm. (**B**) Percentage of SypmOr2-responsive boutons forming filopodia. Line is the mean, and error bar is SEM. (**C**) Percentage of filopodia-forming boutons displaying SypmOr2 responses and percentage of filopodia displaying SypmOr2 responses. Lines are the median, and error bars are interquartile range (IQR). (**D**) Plot of normalized intensity values along the line shown in (A). (**E**) Time-lapse series of an axon coexpressing GPI-EGFP and SypmOr2 during stimulation with 600 APs (5 Hz). White arrow, filopodium. Scale bar, 1 μm. (**F**) Plot of normalized intensity values along the line shown in (E). (**G**) Primary hippocampal neurons were stimulated with 600 APs (40 Hz) and then subjected to high-pressure freezing (HPF) and freeze substitution (FS). 2D view of a filopodium split into 200-nm segments from the tip to the parent axon. (**H**) 3D reconstruction of a filopodium showing segments, endosomes (brown), SVs (cyan), main axon filaments (magenta), and mitochondria (dark green) from 2D tracings of electron micrographs. (**I**) Normalized SV densities within the first four segments from the tip. Boxes are IQR, and whiskers show minimum to maximum. *P* values, Kruskal-Wallis with Dunn’s test. (**J**) Normalized endosome fractions within the first four segments from the tip. Boxes are IQR, and whiskers show minimum to maximum. *P* values, Kruskal-Wallis with Dunn’s test. (**K**) Representative 2D and 3D images of each filopodium and parent axon from three image stacks. Segments are 200 nm long and provide the scale, except for filopodium 10 (2D), for which the scale matches filopodium 11. Sample sizes and exact *P* values are shown in the plots. Summary statistics are provided in table S3.

Filopodia formed exclusively at synapses displaying SypmOr2 responses to stimulation (active synapses), and 78% contained SypmOr2 signal within the protrusion itself (Fig. 1, A and C). In some instances, SypmOr2 fluorescence appeared to flow from the bouton into the filopodium, forming a concentration gradient toward the parent bouton (Fig. 1, A and D). In others, filopodia emerged first and the SypmOr2 signal appeared subsequently at their tip, spatially separated from the bouton (Fig. 1, E and F). This suggests that SV fusion can occur in a subset of filopodia, potentially enabling neurotransmitter release at sites spatially displaced from the parent bouton.

To determine whether filopodia did indeed contain SVs, we next performed an ultrastructural characterization. This combined zap-and-freeze (*23*) fixation of primary hippocampal neurons with serial-section transmission electron microscopy (TEM) and three-dimensional (3D) reconstructions of axonal segments. Neurons were electrically stimulated (600 APs at 40 Hz) and high-pressure frozen within 5 ms following stimulation to capture filopodial morphology at near-native state. We reconstructed 11 presynaptic boutons containing filopodia (Fig. 1, G to K). Filopodia appeared as elongated presynaptic extensions with heterogeneous morphologies and variable numbers of SVs and endosomes distributed evenly along their length (Fig. 1, I and J, and fig. S1, A to C). The presence of SVs corroborates our live imaging results, which showed fusion-competent SVs within filopodia (Fig. 1, A to F). Furthermore, the presence of endosomes suggests membrane retrieval or sorting accompanying filopodia formation/contraction.

We next determined how filopodia formation scales with stimulus load, using two additional stimulation protocols: 600 APs at 5 Hz (identical stimulus number at lower frequency) and 40 APs at 20 Hz [selective mobilization of the readily releasable pool (RRP)] (*24*). Both protocols generated filopodia, indicating that this response is conserved across a range of stimuli (fig. S2, A to G). At 600 APs at 40 Hz, 47% of filopodia formed and disappeared during or within 15 s of stimulation (“transient” filopodia; fig. S2, A and G). This proportion was similar at 600 APs at 5 Hz but increased markedly with 40 APs at 20 Hz (fig. S2, B, C, and G), indicating that sustained activity stabilizes presynaptic filopodia. Shorter trains (40 APs at 20 Hz) also evoked ∼50% fewer filopodia per axon length (fig. S2D), consistent with the reduced total stimulus load. Filopodial length was reduced at lower stimulation frequency (600 APs at 5 Hz) (fig. S2E), while overall morphology, assessed by roundness, was preserved across paradigms (fig. S2F). These results suggest that presynaptic filopodia form during sustained SV fusion and that their stability is correlated with the duration of neuronal activity.

### Neuronal activity promotes presynaptic filopodia formation independently of SV fusion

To further probe the relationship between filopodia formation and neuronal activity, we examined whether filopodia formed during spontaneous, network-driven activity by manipulating endogenous neuronal firing. We either suppressed spontaneous network activity with the ionotropic glutamate receptor antagonists d-2-amino-5-phosphonopentanoic acid (APV) and 6-cyano-7-nitroquinoxaline-2,3-dione (CNQX) or enhanced it with 4-aminopyridine (4-AP), a voltage-gated potassium channel blocker that elevates spontaneous firing rates in both excitatory and inhibitory neurons (*25*). We labeled axonal membranes with GPI-Apple and monitored neuronal activity with the calcium sensor GCaMP6f. Filopodia formation and GCaMP6f responses were abolished by application of APV/CNQX, indicating that filopodia do not arise in the absence of network-driven synaptic transmission (Fig. 2, A and B).

**Fig. 2.**
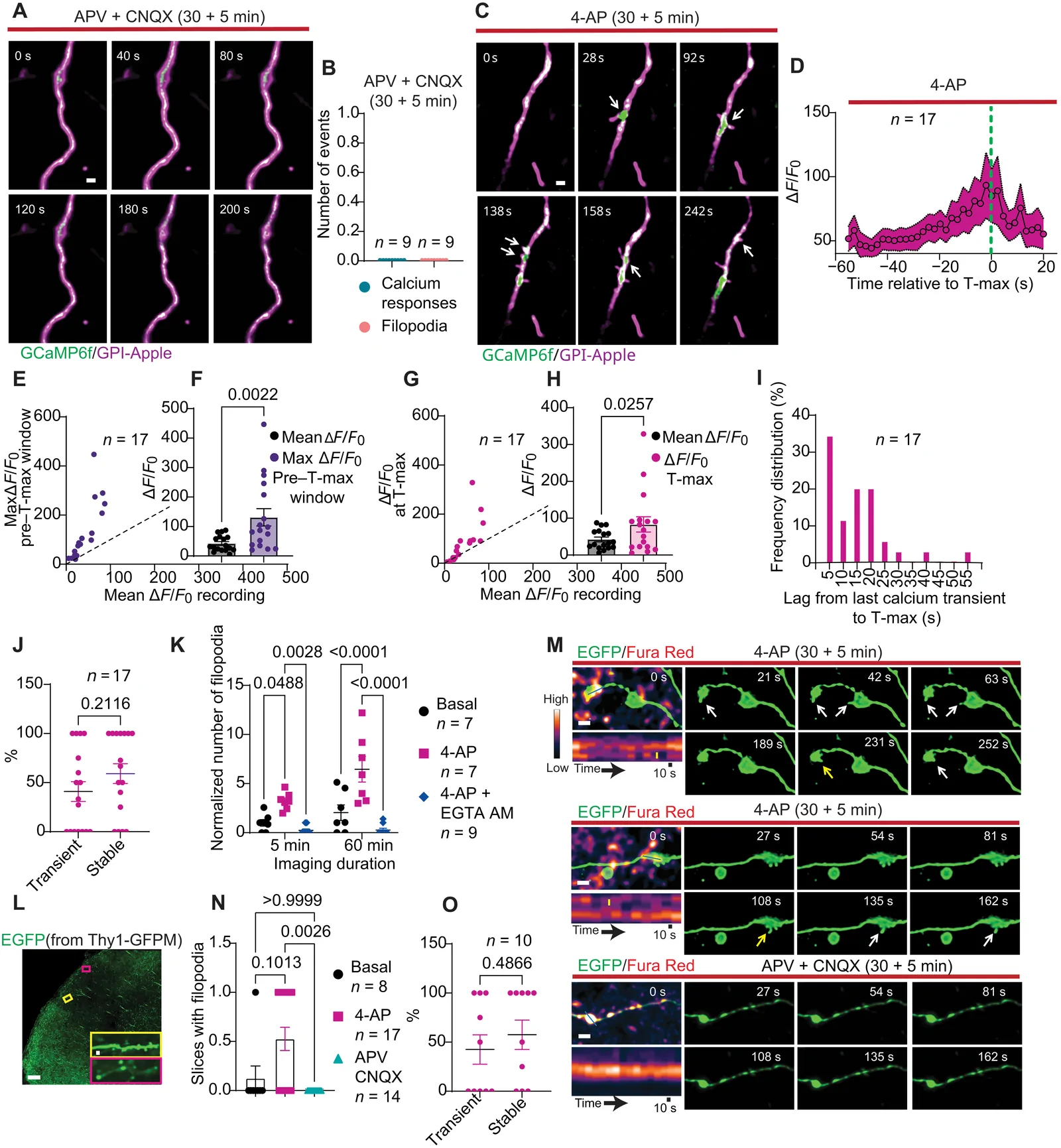
Presynaptic filopodia formation is coupled to neuronal activity in cultures and acute brain slices. (**A**) Axon coexpressing GPI-Apple and GCaMP6f after APV/CNQX treatment. Scale bar, 1 μm. (**B**) GCaMP6f responses and filopodia in APV/CNQX-treated cultures. Lines are the median*.* (**C**) Axon coexpressing GPI-Apple and GCaMP6f after 4-AP treatment. White arrows, filopodia and calcium responses. Scale bar, 1 μm. (**D**) Event-triggered GCaMP6f fluorescence aligned to T-max. Line is mean; shading is SEM. (**E** to **H**) GCaMP6f fluorescence during the 55-s pre–T-max window (E and F) and at T-max (G and H), relative to the recording mean. (E and G) Recording-mean Δ*F*/*F*_0_ versus maximum pre–T-max Δ*F*/*F*_0_ (E) or T-max Δ*F*/*F*_0_ (G). Points above the dashed line indicate above-mean calcium. (F and H) Maximum pre–T-max Δ*F*/*F*_0_ (F) or Δ*F*/*F*_0_ at T-max (H) relative to the recording-mean. Bars are the mean, and error bars are SEM. *P* values, paired *t* test. (**I**) Peak–to–T-max lag intervals in 4-AP–treated neurons. (**J**) Stable-to-transient filopodia ratio after 4-AP treatment. Bars are the mean, and error bars are SEM. *P* values, unpaired *t* test. (**K**) Number of filopodia at boutons during basal activity, 4-AP treatment, or 4-AP following EGTA AM preincubation. Bars are the mean, and error bars are SEM. *P* values, mixed-effects model with Sidak’s test. (**L**) Thy1-GFPM cortical region with sparse EGFP expression. Boxes, axons (pink) and dendrites (yellow). Scale bars, 100 μm (large image) and 2 μm (inset). (**M**) Time-lapse images of Fura Red-loaded neurons treated with 4-AP (top two) or APV/CNQX (bottom). White arrows, filopodia; yellow arrows, filopodia emerging during Fura Red fluorescence decreases depicted in kymographs. Scale bars, 1 μm. (**N**) Number of filopodia formed along axons in slices. Lines are the median, and error bars are IQR. *P* values, Kruskal-Wallis with Dunn’s test*.* (**O**) Stable-to-transient filopodia ratio in 4-AP–treated slices. Bars are the mean, and error bars are SEM. *P* values, unpaired *t* test.

In 4-AP–treated cultures, the proportion of transient versus stable filopodia was comparable to that observed with electrical field stimulation (Fig. 2J), indicating consistency across stimulation paradigms. Furthermore, filopodia formation was tightly preceded by elevated axonal calcium (Fig. 2C and movie S2). We aligned event-triggered averages to the time of maximum filopodial extension (T-max) and observed a marked increase in GCaMP6f fluorescence during the period leading up to T-max, with the mean signal peaking immediately before T-max and remaining elevated at T-max itself (Fig. 2D). In the large majority of cells, GCaMP6f fluorescence exceeded the recording-wide mean within the 55-s pre–T-max window (94% of cells) and at T-max itself (77% of cells) (Fig. 2, E to H), indicating that filopodia form preferentially during periods of increased activity. When we could identify discrete preceding calcium peaks within the 55-s pre–T-max window, the peak-to-T-max lag distribution showed a strong skew toward short periods (Fig. 2I), further suggesting that filopodial extension closely follows calcium transients.

To further test whether increased neuronal activity promotes presynaptic filopodia formation and whether intracellular calcium elevations contribute to this response, we compared cultures under basal conditions with cultures treated with 4-AP, either alone or following a 15-min preincubation with the cell-permeant intracellular calcium chelator EGTA AM (acetoxymethyl ester) (Fig. 2K). We quantified filopodial number, length, and morphology at 5 and 60 min after treatment. The number of filopodia was increased by 4-AP relative to basal conditions at both time points, whereas EGTA AM markedly reduced this increase (Fig. 2K). Filopodial length and morphology remained comparable between basal and 4-AP–treated cultures (fig. S3, A and B). After 60 min, however, 4-AP occasionally induced multiple filopodia from individual boutons and more complex structures, including branched and rebranched collateral-like protrusions (fig. S3C). These results indicate that increased neuronal activity promotes presynaptic filopodia formation and that elevations in intracellular calcium support this response.

To test whether filopodia formation requires neuronal activity per se or can be driven by SV fusion alone, we used two complementary pharmacological manipulations that decouple these events. First, we triggered activity- and calcium-independent SV fusion by applying a 5-s hypertonic sucrose pulse (500 mM) to preferentially mobilize the RRP (*26*). This maneuver failed to produce filopodia, generating only a minor enlargement of synaptic boutons (fig. S4, A and B). This contrasts to electrical field stimulation with 40 APs at 20 Hz, which mobilizes the same SV pool yet reliably produces filopodia (fig. S4B). Second, we blocked SV exocytosis with tetanus toxin (TeNT), which cleaves synaptobrevin 2, the vesicular soluble *N*-ethylmaleimide–sensitive factor attachment protein receptor protein essential for fusion (fig. S4, C to H) (*27*). TeNT leaves activity-dependent calcium influx intact (*28*) but, as anticipated, eliminated evoked SypmOr2 responses (fig. S4E). Under these conditions, filopodial morphology was preserved, but both number and length were reduced (fig. S4, F to H). Activity-induced filopodia formation persisted in some neurons despite complete blockade of SypmOr2 responses (fig. S4, E and F). These data indicate that neuronal activity is necessary for filopodia formation, whereas SV fusion alone is insufficient, although it may contribute to elongation by supplying additional membrane material.

### Presynaptic filopodia form in response to increased neuronal activity within intact brain tissue

We next investigated whether activity-dependent presynaptic filopodia form de novo within intact brain circuits. To achieve this, we performed high-resolution time-lapse imaging of EGFP-labeled excitatory boutons in the superficial cortical layers of acute coronal slices from Thy1-GFPM mice (Fig. 2, L and M). Sparse EGFP expression in these regions enabled clear visualization of individual en passant and terminal boutons. To monitor local neuronal activity, we preloaded slices with the calcium indicator Fura Red, permitting coarse measurement of calcium fluctuations within imaged boutons (Fig. 2M). We compared bouton dynamics under three conditions: basal network activity, treatment with 4-AP to elevate network activity, or treatment with APV/CNQX to silence it (Fig. 2, M to O).

We observed no structural remodeling or Fura Red signal fluctuations in APV/CNQX-treated slices (Fig. 2, M and N). Under basal activity conditions, boutons also showed little structural remodeling over the imaging period (Fig. 2N). In contrast, in 4-AP–treated slices, ∼53% of boutons transiently extended and retracted filopodia-like protrusions that were accompanied by transient decreases in Fura Red fluorescence, consistent with local calcium elevation (Fig. 2, M and N). The ratio of transient versus stable filopodia in situ was comparable to that in dissociated cultures (Fig. 2O). Filopodial length was less variable than in dissociated cultures (fig. S5), likely reflecting differences in the mechanical environment of intact tissue. These data demonstrate that presynaptic filopodia rapidly form and retract in an activity-dependent manner within the preserved circuit architecture of acute mammalian brain slices.

### Presynaptic filopodia form transient contacts (kinapses) with postsynaptic targets

Because SV fusion can occur within a subset of filopodia at sites displaced from the parent bouton (Fig. 1, C, E, and F), we next asked whether these protrusions contact postsynaptic structures to transiently expand synaptic connectivity. To confirm their synaptic origin and determine whether they form at both excitatory and inhibitory presynapses, we performed post hoc immunolabeling against Bassoon (Bsn), a core constituent of the cytomatrix at the active zone, and vGAT, a marker of inhibitory presynapses, with parallel labeling of postsynaptic AMPA receptors in GPI-EGFP–transfected neurons (GluA subunits; Fig. 3, A to F). We detected fewer filopodia in fixed cultures than during live imaging, but filopodial length and morphology remained comparable between preparations (fig. S6, A to C). Most filopodia-forming boutons contained a Bsn signal (81%), with 46% of filopodia showing Bsn immunoreactivity along their core (Fig. 3D). A substantial fraction of filopodia-forming synapses was vGAT positive (30%), with an equivalent proportion of filopodia containing vGAT signal along their central axis (Fig. 3E), indicating that filopodia formation is not restricted to excitatory synapses. Furthermore, 59% of filopodia-forming boutons were GluA positive, and 50% of filopodia overlapped with GluA puncta (Fig. 3F), suggesting that filopodia extend toward and along postsynaptic structures.

**Fig. 3.**
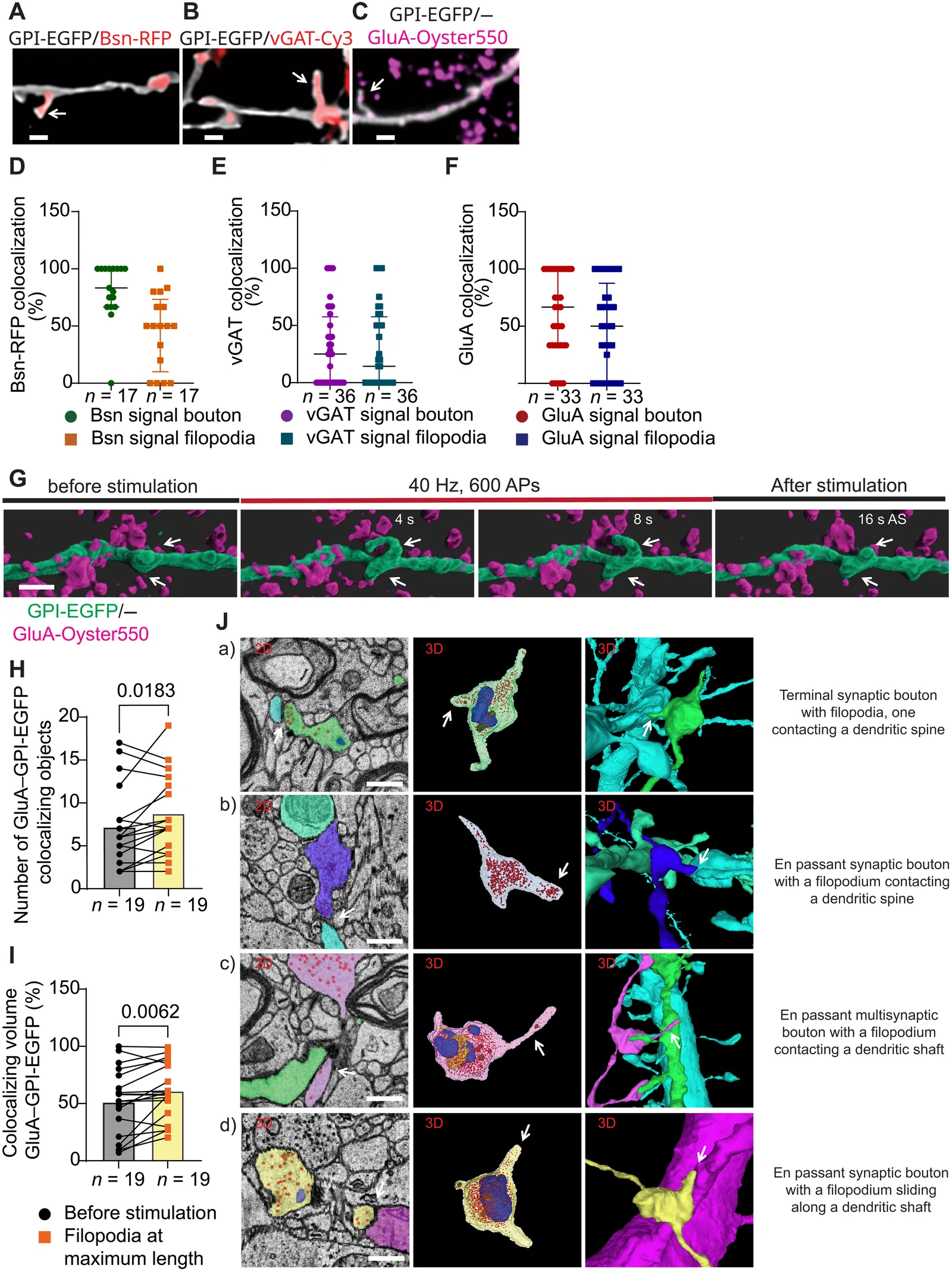
Presynaptic filopodia form kinapses with postsynaptic targets in cultured neurons and are present in human cortex. (**A**) Representative overlay of a hippocampal neuron cotransfected with GPI-EGFP and Bsn-RFP, stimulated with 600 APs (40 Hz), fixed, and imaged. White arrows, filopodia colocalizing with Bsn-RFP. Scale bar, 1 μm. (**B**) Overlay image of a neuron transfected with GPI-EGFP and live-labeled with Cy3-conjugated anti-vGAT antibody. White arrows, colocalizing filopodia. Scale bar, 1 μm. (**C**) Overlay image of a neuron transfected with GPI-EGFP and live-labeled with Oyster550-conjugated anti-GluA antibody. White arrows, colocalizing filopodia. Scale bar, 1 μm. (**D** to **F**) Percentages of filopodia-forming boutons and filopodia colocalizing with Bsn-RFP (D), vGAT-Cy3 (E), and GluA-Oyster550 (F). Lines are the median, and error bars are IQR. (**G**) Confocal time-lapse series showing kinapses between filopodia and GluA-labeled postsynapses during 600-AP (40-Hz) stimulation. White arrows indicate filopodia establishing kinapses. Scale bar, 1 μm. (**H**) Quantification of GluA–GPI-EGFP colocalizing objects at rest versus at maximum filopodial extension. Bars are the mean. *P* values, paired *t* test. (**I**) Quantification of GluA-GPI-EGFP overlap volume at rest and at maximum filopodial extension. Bars are the mean. *P* values, paired *t* test. (**J**) Representative 2D views (left) and 3D reconstructions (middle and right) of synaptic boutons with putative filopodia from human cerebral cortex. White arrows, filopodia. Middle: 3D reconstructions of synaptic boutons with filopodia showing SVs (red), endosomes (brown), mitochondria (blue), and endoplasmic reticulum (yellow) from 2D tracings of electron micrographs. Right: 3D reconstructions of the same filopodia forming contacts with dendritic spines (a and b) or dendritic shafts (c and d) and thereby enhancing connectivity to a single (a and d) or multiple neurons (b and c). Scale bars, 500 nm.

To determine whether this spatial proximity reflects dynamic contact, we examined filopodial colocalization with GluA-labeled postsynapses in real time during electrical field stimulation (600 APs at 40 Hz; Fig. 3, G to I). Both the number of colocalizing GPI-EGFP–GluA objects and their volumetric overlap increased significantly during filopodial extension (Fig. 3, H and I), showing that filopodia contact postsynaptic receptor clusters as they form. We term these dynamic contacts “neuronal kinapses” (from kine-, movement, and *-*apse, fastening) (*29*) to distinguish them from stable synaptic connections. Therefore, neuronal activity rapidly induces presynaptic filopodia that transiently expand potential synaptic connectivity.

The kinapse contacts observed in vitro prompted us to ask whether presynaptic filopodia represent a conserved structural feature of mammalian synapses. To test this, we examined their ultrastructural correlates in the human cerebral cortex using a recently released volumetric electron microscopy (EM) dataset of temporal cortex obtained intraoperatively from an epilepsy patient undergoing resection to access a hippocampal lesion (*30*). Within this dataset, which contains numerous automatically reconstructed neurons and their synaptic connections, we manually identified and reconstructed presynaptic boutons exhibiting filopodia-like extensions (Fig. 3J). These structures were readily identifiable, appearing either as short protrusions (∼500 nm) deforming the bouton membrane (Fig. 3J, a, b, and d) or as longer processes reminiscent of nascent collateral branches extending from the presynapse (Fig. 3J, c). As observed in dissociated neurons, these protrusions contained variable numbers of SVs and occasional endosomal structures (Fig. 3J, a to d). The identified filopodia frequently established atypical presynaptic contacts with both spiny and nonspiny postsynaptic targets, in some cases connecting multiple neurons simultaneously, closely resembling the kinapse-like appositions identified in vitro (Fig. 3J). Presynaptic filopodia therefore represent a conserved morphological feature of mouse and human cortex. Their presence in human cortex suggests that the transient multisynaptic connectivity supported by filopodia in vitro may also occur in intact human brain tissue.

### Presynaptic filopodia formation favors synchronous over asynchronous neurotransmitter release during sustained SV fusion

Filopodial extension deforms the membrane of the parent bouton and may therefore influence synaptic function, specifically neurotransmitter release. To determine this, we imaged individual boutons with or without filopodia on the same axon, under identical stimulation conditions, using iGluSnFR3.v857, a fast, nonsaturating glutamate sensor with improved synaptic specificity (*31*). High-speed total internal reflection fluorescence microscopy (TIRF) imaging during 300-AP, 20-Hz trains resolved both synchronous and asynchronous iGluSnFR3.v857 responses, with the membrane-anchored sensor enabling simultaneous tracking of filopodial extension due to its bright axonal labeling (Fig. 4, A and B). To ensure all detected events originated from stimulation-evoked release at the imaged boutons, we suppressed spontaneous network activity with APV/CNQX throughout, evidenced by a release-free prestimulus baseline (Fig. 4A). Synchronous release was quantified during the stimulation train and asynchronous release in a window occurring at least 10 ms after the final AP, consistent with the broad temporal range of delayed fusion events encompassed by this term in the literature (*32*, *33*). At 20 Hz, synchronous fusion tightly coupled to each AP dominates the iGluSnFR3.v857 signal during the train, masking the comparatively small and stochastic asynchronous component and precluding its reliable quantification. The poststimulus window reduces this ambiguity, as, with no further APs driving synchronous fusion, all detected iGluSnFR3.v857 events are by definition asynchronous.

**Fig. 4.**
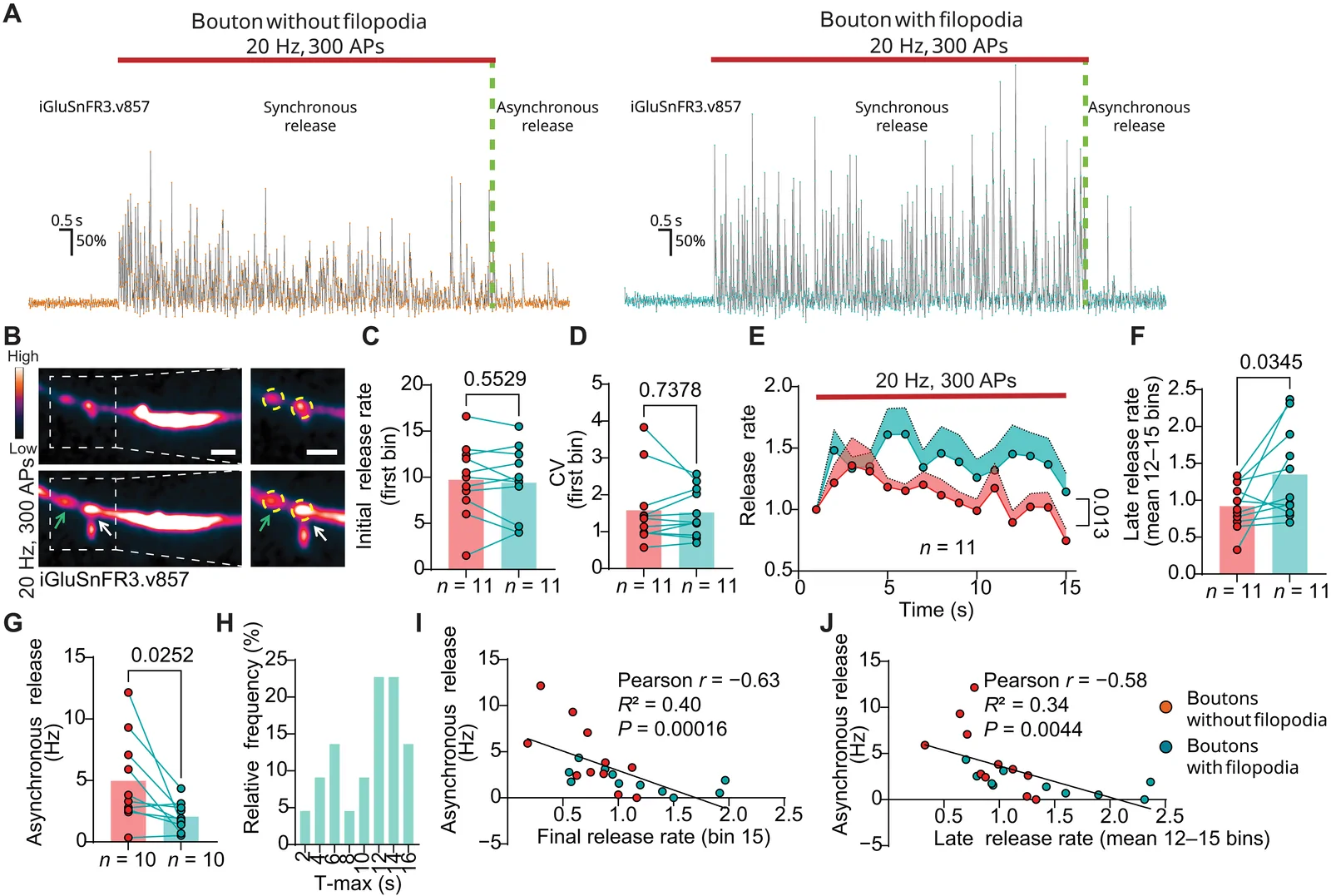
Presynaptic filopodia formation is associated with increased synchronous and reduced asynchronous neurotransmitter release. (**A**) iGluSnFR3.v857 responses at individual boutons without (left) and with filopodia (right) during a 300-AP (20-Hz) stimulation train, showing stimulation period and posttrain asynchronous window. (**B**) TIRF imaging of representative neurons expressing iGluSnFR3.v857 enabling simultaneous detection of glutamate release and filopodia formation. Insets (yellow dashed circles) show representative boutons without (green arrow) and with filopodia (white arrow). Scale bars, 1 μm. (**C**) Release rate in the first second of stimulation at boutons with and without filopodia. Bars are the mean. *P* values, paired *t* test. (**D**) CV of release rate in the first second of stimulation. Bars are the mean. *P* values, paired *t* test. (**E**) Synchronous release rate across successive 1-s bins of the stimulation train at boutons with and without filopodia. Lines are the mean, and error bars are SEM. *P* values, repeated-measures ANOVA with Sidak’s test. (**F**) Late release rate (mean across bins 12 to 15 s) at boutons with and without filopodia, compared within individual neurons. Bars are the mean. *P* values, paired *t* test. (**G**) Posttrain asynchronous iGluSnFR3.v857 event frequency at boutons with and without filopodia. Bars are the mean. *P* values, paired *t* test. (**H**) Histogram of the T-max relative to stimulus onset. (**I**) Correlation between release rate at the final second of the train and posttrain asynchronous event frequency across neurons. (**J**) Correlation between late release rate (bins 12 to 15 s) and posttrain asynchronous event frequency across neurons.

Release rate was calculated as the frequency of synchronous fusion events, binned in 1-s intervals to resolve short-term plasticity across the AP train. At train onset, filopodia-forming and nonforming boutons were functionally indistinguishable. Release rate in the first second of stimulation was equivalent between bouton types (Fig. 4C), as was the coefficient of variation (CV) (Fig. 4D), a measure of release variability. Furthermore, the parameters of individual initial fusion events including peak response amplitude, full width at half maximum (FWHM), and delay to first response were all comparable (fig. S7, A to C). This equivalence at train onset suggests that filopodia-forming boutons are unlikely to constitute a preexisting high release probability (*Pr*) subpopulation.

As stimulation continued, the two populations diverged (Fig. 4E). Filopodia-forming boutons showed progressive facilitation across successive seconds of the AP train, whereas nonforming boutons maintained initial release rates before depressing toward the end of the stimulation period (Fig. 4E). At the cell level, boutons that formed filopodia during the train showed a significantly higher late release rate, defined as the mean release rate across bins 12 to 15 s, than nonforming boutons from the same neuron (Fig. 4F). This divergence was temporally consistent with filopodia formation: Filopodia initiated gradually after stimulus onset and reached maximum length at various times across the train, on average ∼10 s after stimulus onset (Fig. 4H), spanning the period over which release rate diverged between bouton types. Filopodia-forming boutons also showed a concomitant reduction in posttrain asynchronous event frequency (Fig. 4G), while amplitudes of individual asynchronous events were identical between groups throughout (fig. S7D). This indicates that the shift likely reflects a change in *Pr* rather than SV content.

The reciprocal relationship between sustained synchronous release and posttrain asynchronous frequency was not simply a group difference but a continuous bouton-level relationship. Across all cells regardless of filopodia status, posttrain asynchronous release frequency was negatively correlated with late release rate (Fig. 4, I and J), with the strongest association at the final second of the AP train (Fig. 4I), which was confirmed against late release rate (Fig. 4J) and cumulative release across the full train (fig. S7E). In this continuum, boutons maintaining higher release late in the AP train exhibit lower posttrain asynchronous release, consistent with the principle that sustained synchronous release efficacy reduces the asynchronous release fraction at individual synapses. Filopodia-forming boutons thus occupied the high-synchronous, low-asynchronous release extreme of this distribution, implicating activity-dependent structural remodeling as a potential contributor to the synchronous-to-asynchronous release ratio at individual synapses.

Filopodia are canonically defined as membrane protrusions that elongate through actin polymerization at their tips. In agreement, LifeAct-Ruby expression showed that filamentous actin is present in virtually all stimulus-evoked presynaptic filopodia (fig. S8, A and B), with continuous and treadmilling filaments linking the filopodial core to the synaptic actin meshwork (fig. S8A). To identify tools for loss-of-function experiments, we screened compounds targeting distinct molecular pathways: dynamin-mediated membrane remodeling (dynasore) (*34*), formin-dependent actin polymerization (SMIFH2) (*35*), and NHE-coupled actin reorganization [EIPA, its analog amiloride, and short hairpin RNA (shRNA)–mediated NHE1 knockdown] (*36*–*39*). NHE inhibition was prioritized as a candidate based on its established role in actin-dependent membrane protrusion: rapidly retracting invasive epithelial cell protrusions (*38*) and impairing growth cone filopodia and neurite extension in developing neurons (*37*). Broad actin-modulating compounds were deliberately avoided given their pleiotropic effects.

We assessed the effects of these interventions by monitoring filopodia generation during electrical field stimulation with 600 APs at 40 Hz (Fig. 5, A to E). SMIFH2 produced a partial reduction in filopodial number and length without altering their shape, whereas dynasore had no significant effect on any parameter (Fig. 5, A to D). EIPA and amiloride were particularly effective, abolishing nearly all stimulus-evoked filopodia, without altering the morphology of the few filopodia that still emerged (Fig. 5, A to D). None of the interventions altered the stable-to-transient ratio (Fig. 5E), synaptic ultrastructure (fig. S9A), SV distribution (fig. S9B), docked SV number (fig. S9C), number of endosomes (fig. S9D), or SV size (fig. S9E), indicating that gross bouton morphology was unaffected. shRNA-mediated NHE1 knockdown also reduced filopodial number without altering filopodial length, shape, or stable-to-transient ratio (fig. S10, A to G), suggesting a specific contribution of NHE activity to filopodia formation. Therefore, filopodia formation appears to require activity-dependent NHE-coupled actin reorganization.

**Fig. 5.**
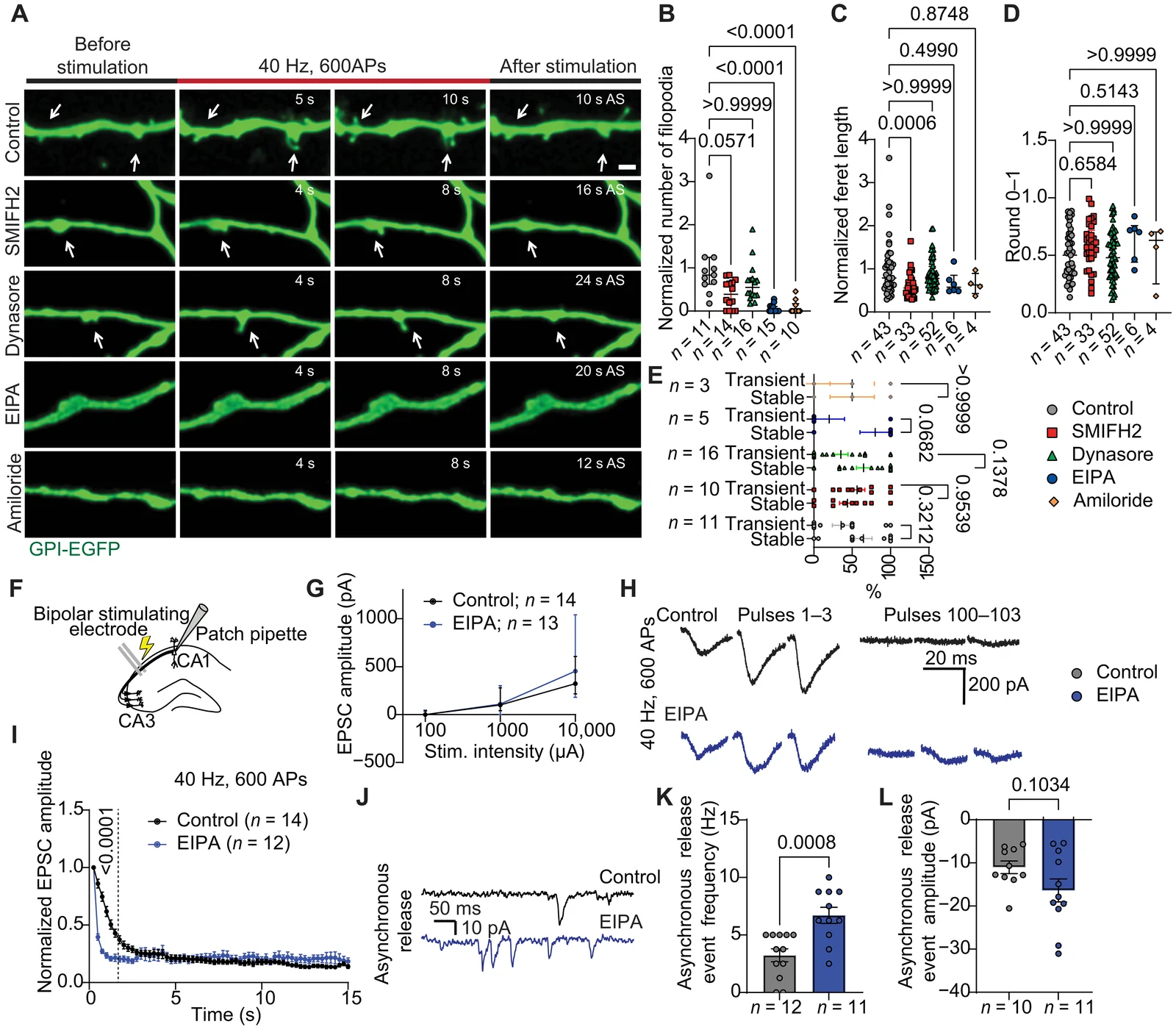
NHE inhibition suppresses presynaptic filopodia formation and promotes asynchronous neurotransmitter release. (**A**) Confocal time-lapse series showing filopodia formation (white arrows) in neurons transfected with GPI-EGFP and treated for 30 min with vehicle, SMIFH2, EIPA, and amiloride or acutely with dynasore for 2 to 4 min. Neurons were stimulated with 600 APs (40 Hz). Scale bar, 1 μm. (**B**) Normalized number of filopodia per 10-μm axon length. Lines are the median, and error bars are IQR. *P* values, Kruskal-Wallis with Dunn’s test. (**C**) Normalized filopodial length. Lines are the median, and error bars are IQR. *P* values, Kruskal-Wallis with Dunn’s test. (**D**) Quantification of filopodial shape. Lines are the median, and error bars are IQR. *P* values, Kruskal-Wallis with Dunn’s test. (**E**) Ratio of stable to transient filopodia. Lines are the mean, and error bars are SEM. *P* values, two-way ANOVA with Sidak’s test. (**F** to **L**) Synaptic transmission at CA3-CA1 synapses assessed by whole-cell patch-clamp recordings in wild-type mouse brain slices with (blue) or without (black) 50 μM EIPA for 30 min. (F) Schematic of recording configuration. (G) Input-output plot of stimulation intensity versus EPSC amplitude. Symbols are the mean, and error bars are SEM. *P* values, two-way ANOVA with Sidak’s test. (H) Representative EPSC traces during 600-AP, 40-Hz stimulation. (I) Synaptic rundown during 600-AP, 40-Hz stimulation. Circles are the mean; error bars are SEM. *P* values, mixed-effects model with Sidak’s test. (J) Representative traces of asynchronous release events occurring after high-frequency stimulation in control and EIPA-treated conditions. (K) Analysis of posttrain asynchronous release frequency. Bars are the mean, and error bars are SEM. *P* values, unpaired *t* test. (L) Amplitude of posttrain asynchronous events. Bars are the mean, and error bars are SEM. *P* values, unpaired *t* test.

To test whether blocking filopodia formation with EIPA or amiloride alters SV fusion, we monitored synaptophysin-pHluorin (SypHy; which operates in an identical manner to SypmOr2) fluorescence during consecutive 40- and 900-AP trains at 20 Hz, which mobilize the RRP and total releasable SV pool, respectively (fig. S11, A to C). We used bafilomycin A1 to prevent SV reacidification (*40*), which ensured that increases in SypHy fluorescence reported cumulative SV exocytosis (fig. S11A). In control neurons, SypHy fluorescence increased and reached a plateau during both stimuli. EIPA and amiloride delayed this plateau and prolonged the fluorescence increase after stimulation, particularly following the second train, which mobilized the total releasable SV pool (fig. S11, A to C). Thus, EIPA and amiloride slowed SV fusion during stimulation and increased asynchronous fusion after stimulation. Inhibiting filopodia formation with EIPA and amiloride did not significantly affect the amplitude of stimulus-evoked intracellular free calcium responses measured with GCaMP6f or desynchronize axonal calcium transients (fig. S11, D and E).

To more directly determine how inhibition of filopodia generation affects synchronous and asynchronous neurotransmission, we performed whole-cell patch-clamp recordings in the hippocampal CA3-CA1 pathway in the presence or absence of EIPA. In agreement with the bouton-level relationship between filopodia formation and sustained release rate, acute EIPA application shifted neurotransmitter release kinetics in the direction predicted by the loss of filopodia (Fig. 5, F to L). EIPA did not alter evoked excitatory postsynaptic current (EPSC) amplitudes to a range of stimulus intensities (Fig. 5G), indicating that basal synaptic transmission and postsynaptic responsiveness were preserved. During high-frequency stimulation (600 APs at 40 Hz), however, EPSC amplitudes in EIPA-treated slices decayed more rapidly than in controls (Fig. 5, H and I), suggesting that sustained synchronous transmission is compromised under conditions in which filopodia formation is suppressed. High-frequency stimulation also evoked asynchronous release, persisting after the cessation of presynaptic firing (Fig. 5J). Although these delayed events were present under both conditions, their frequency was significantly increased by EIPA, while event amplitude was unchanged (Fig. 5, K and L). Thus, suppression of filopodia formation is associated with reduced synchronous release and a shift toward asynchronous release during sustained activity.

### A convection-diffusion model for filopodial effects on synchronous versus asynchronous release

How might filopodia formation contribute to sustained synchronous release? During repetitive stimulation, facilitation or depression of neurotransmitter release arises from a combination of RRP replenishment/depletion and the clearance or accumulation of vesicular components at release sites. At high stimulus frequencies, SV proteins and lipids cannot be retrieved promptly, becoming stranded at the active zone, blocking release sites, and contributing to synaptic depression (*41*). Membrane protrusions and contractions are known to generate tension gradients capable of redistributing membrane-associated molecules over long distances (*13*), raising the possibility that filopodial extension from the bouton surface facilitates release-site clearance during sustained activity.

We hypothesize that filopodial extension promotes convective in-plane membrane flow, which facilitates release-site clearance by moving vesicular components from the AZ toward the periactive zone, alleviating depression during repeated SV fusion (Fig. 6A). Supporting this, live imaging demonstrated directed movement of SypmOr2 toward extending filopodia during synaptic activity (Fig. 1, A and D).

To test whether this flow can quantitatively account for the observed facilitation and shift toward synchronous release, we first created a mathematical model (Fig. 6, A to E). Assuming that an in-plane convection exists, we deduced a threshold speed for this flow to quantitatively reproduce the observed facilitation of synchronous release. To address this and to link the model to our experiments, we developed two coupled modules.

**Fig. 6.**
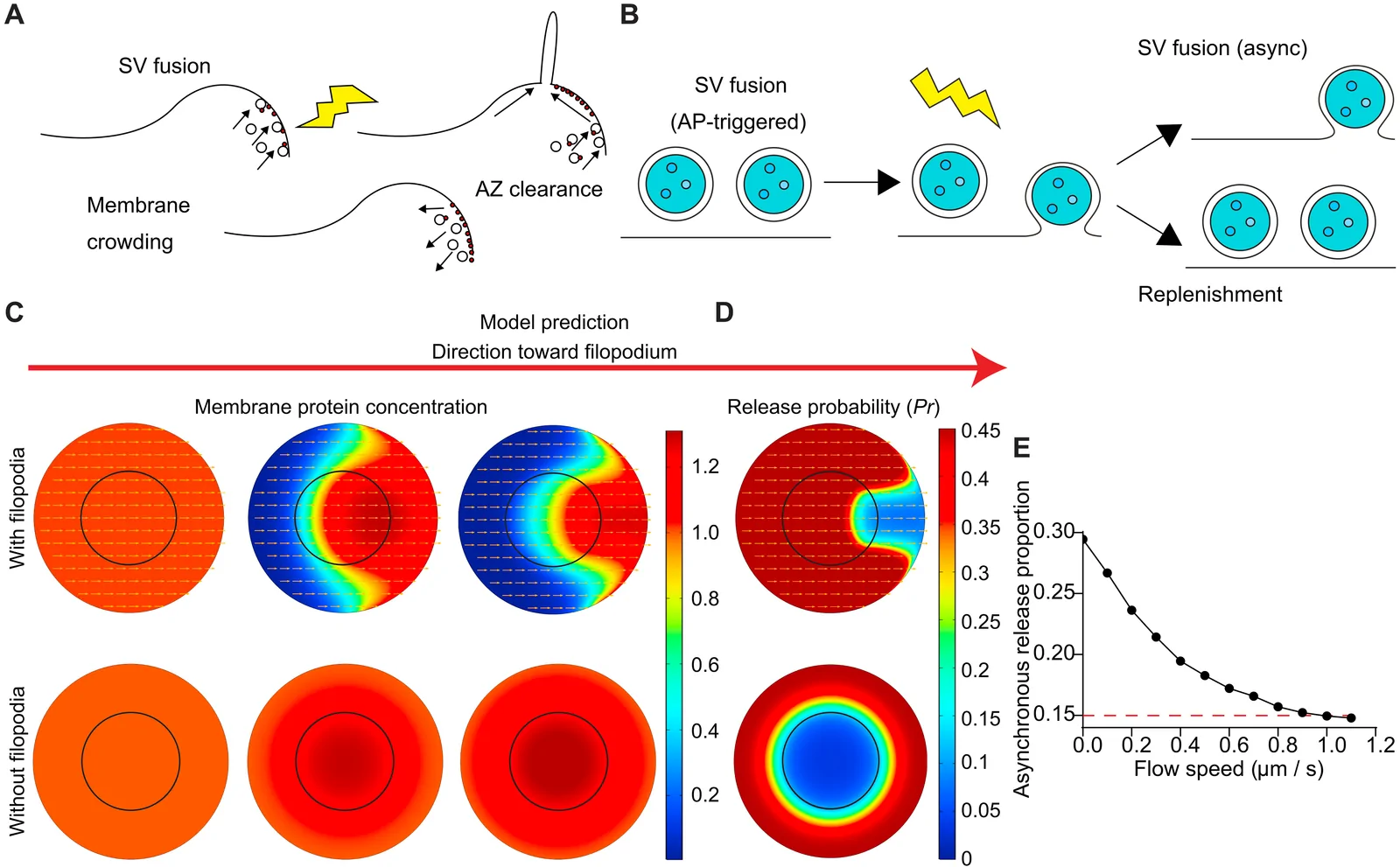
A convection-diffusion model for filopodial effects on synchronous versus asynchronous release. (**A**) Schematic: Filopodial extension generates in-plane membrane flow that clears vesicular components stranded at the AZ during repeated SV fusion, directing them toward the periactive zone. (**B**) Stochastic SV-fusion model: After an AP, the RRP undergoes probabilistic fusion and replenishment; release events occurring after 10 ms are classified as asynchronous. (**C** and **D**) Predicted spatiotemporal profiles of vesicular components (C) and corresponding release probabilities (D) at 400 ms in boutons with or without filopodia. (**E**) The model predicts that asynchronous release proportion decreases with increasing in-plane membrane flow rate. Assuming that convective speed without filopodia is 0 μm s^−1^, the model predicts that a speed of ∼1 μm s^−1^ is necessary to quantitatively capture the twofold decrease in asynchronous release upon filopodial extension (red dashed line).

#### Convection-diffusion module

Vesicular components were modeled as undergoing convection due to in-plane membrane flow while diffusing freely along the presynaptic terminal. A 2D presynaptic terminal was defined comprising an AZ with a diameter of 0.3 μm surrounded by a periactive zone of 0.15 μm in width. This geometry was used to simulate the dynamic spatiotemporal distribution of vesicular components during a 20-Hz AP train. Exocytosis was restricted to the AZ and promoted by increases in *Pr*; removal occurred at the periactive zone boundary until concentrations returned to baseline. Model parameters were constrained by existing measurements of protein diffusion (*42*) and the known AZ/periactive zone dimensions (*43*). We examined the effect of in-plane membrane flow by varying flow rates.

#### Stochastic release module

The average simulated steady-state *Pr* in the AZ was used as the representative *Pr* for boutons with or without filopodia. These representative release probabilities were input into a stochastic simulation of SV fusion to assess filopodial impact on synchronous versus asynchronous event frequency. In this simulation, the boutons randomly underwent SV fusion and RRP replenishment following a 1-ms AP at the start of the simulation. We assumed that this AP was the last in a train of APs, and we modeled the effect of extended stimulation on RRP depletion by initializing the RRP as half-full. Release events occurring within 10 ms after the AP were classified as synchronous, while those after 10 ms were considered asynchronous.

Our simulation indicated that the in-plane membrane flow substantially altered the spatiotemporal distribution of vesicular components, facilitating release-site clearance (Fig. 6C and movies S3 and S4). This increased *Pr* (Fig. 6D and movies S5 and S6) and reduced RRP depletion over hundreds of milliseconds, resulting in more synchronous and fewer asynchronous release events (Fig. 6, D and E). To quantitatively reproduce the experimentally observed increase in synchronous release and a reduction in asynchronous release, the model predicted that an in-plane membrane flow rate in the range of ∼1 μm s^−1^ was required (Fig. 6E).

### Activity-induced presynaptic filopodia generate convective membrane flow

We next tested the model prediction experimentally. Live imaging had already indicated redistribution of SypmOr2 toward extending filopodia during synaptic activity (Fig. 1, A and D), suggesting that filopodial extension drives directed redistribution of SV protein away from the site of release. To quantify this, we placed regions of interest (ROIs) on the filopodial and distal sides of boutons and tracked SypmOr2 fluorescence over time during electrical field stimulation (600 APs at 40 Hz; Fig. 7, A and B). We constructed two complementary measures: a normalized enrichment index (EI), capturing the fractional bias of SypmOr2 signal toward the filopodial compartment independent of absolute expression levels between boutons (Fig. 7C), and a directional flux, reflecting the rate at which vesicular membrane protein accumulates at the filopodial side at the expense of the distal side over the stimulation period (Fig. 7D). In filopodia-forming boutons, the EI rose progressively, and the flux increased significantly over the stimulation period, together indicating directional redistribution of SypmOr2 toward the filopodium (Fig. 7, C and D). This redistribution was absent in control boutons lacking filopodia, where ROIs placed symmetrically on opposite sides of the bouton showed neither a rising EI nor a significant flux (fig. S12, A to D), demonstrating that progressive accumulation of SypmOr2 toward the filopodium is likely a specific consequence of filopodial extension.

**Fig. 7.**
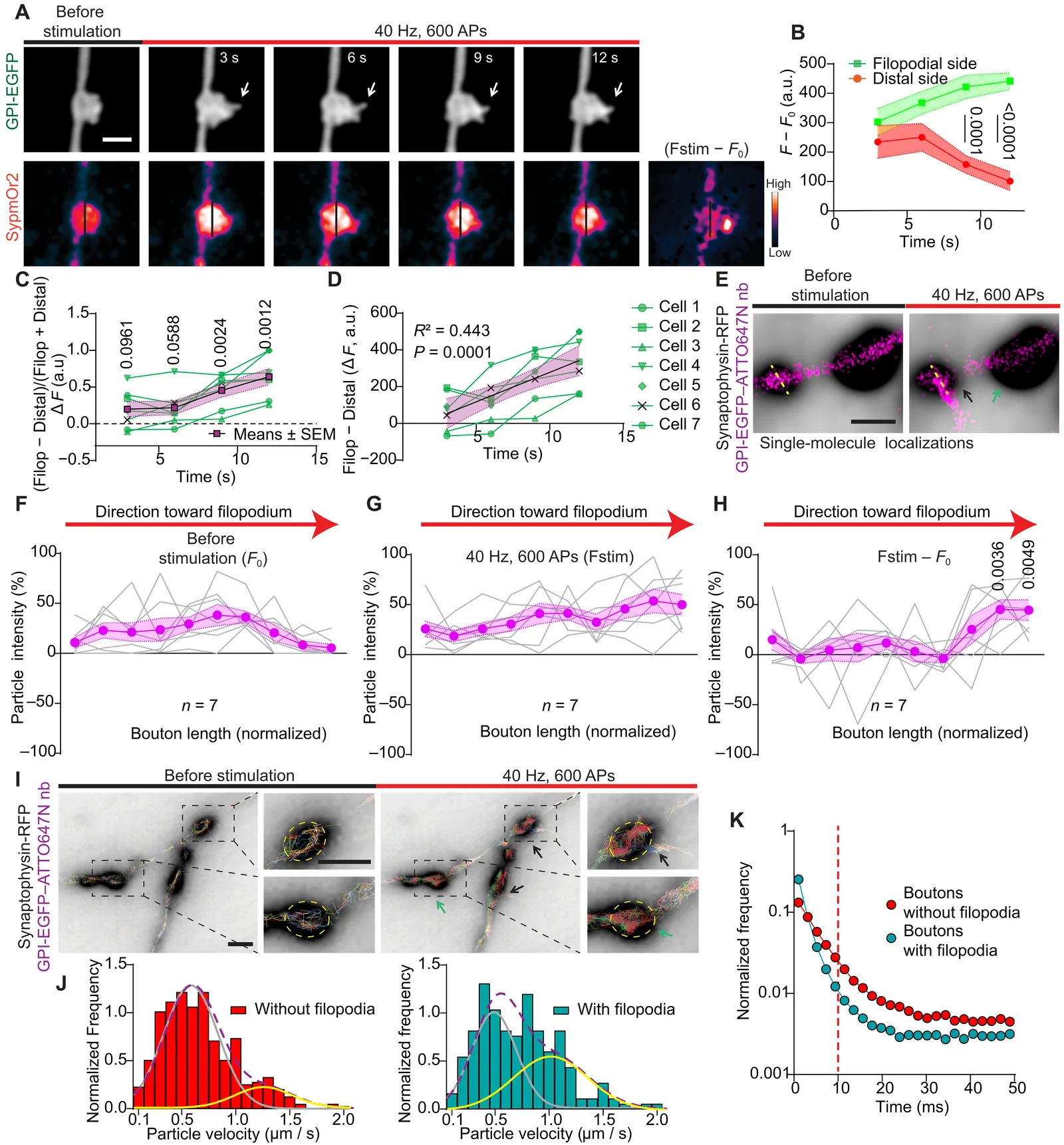
Activity-induced presynaptic filopodia generate convective membrane flow. (**A**) Hippocampal neuron coexpressing GPI-EGFP and SypmOr2 before and during electrical field stimulation (600 APs at 40 Hz). Black bars separate the filopodial (white arrow) and distal sides. Right: Difference image showing SypmOr2 accumulation toward the filopodium. Scale bar, 1 μm. (**B**) SypmOr2 fluorescence at filopodial and distal ROIs during stimulation (means ± SEM). *P* values, mixed-effects model with Sidak’s test. (**C**) Normalized EI for individual cells (green lines) and means ± SEM (filled symbols). Positive values denote accumulation toward filopodia. *P* values, one sample *t* test. (**D**) Pooled linear regression of flux over time. Cell traces, green; 95% confidence interval, shading. (**E**) Super-resolved uPAINT images of surface GPI-EGFP labeled with ATTO647N anti-GFP nanobody (magenta) over synaptophysin-RFP (gray), before and during stimulation. Black arrow, filopodium-forming bouton; green arrow, bouton without filopodia. Lines, intensity-profile axes. Scale bar, 1 μm. (**F** to **H**) GPI-EGFP–ATTO647N intensity profiles along the boutons oriented toward the filopodia before (F) and during (G) stimulation and their difference (H). Individual cells, gray; purple shaded band, means ± SEM. Intensity and length were normalized independently to 0 to 100%. *P* values, one-sample *t* test against zero. (**I**) uPAINT trajectories of GPI-EGFP–ATTO647N (rainbow) over synaptophysin-RFP–labeled boutons (gray). Yellow dashed circles, boutons; insets, boutons with (black arrows) or without (green arrows) filopodia. Scale bars, 1 μm. (**J**) Particle-velocity distributions at boutons without (left) and with (right) filopodia, fitted by two Gaussians (gray and yellow; combined fit, purple). Gaussian components (mean velocity in μm/s, weight): without filopodia (left), gray (0.55, 0.85) and yellow (1.27, 0.15); with filopodia (right), gray (0.48, 0.53), and yellow (0.98, 0.47). (**K**) Model-predicted release at measured flow speeds with or without filopodia. Release after 10 ms is asynchronous (orange dashed line). Asynchronous proportion: with filopodia, 0.15; without, 0.30.

Because SV components rapidly fuse and are retrieved during synaptic transmission, quantification of presynaptic membrane diffusion rates and potential convective flow at filopodia-forming boutons required an alternative method. We therefore used universal point accumulation for imaging in nanoscale topography (uPAINT), a live-cell single-molecule super-resolution method, with a GPI-anchored reporter (Fig. 7, E to J). We imaged hippocampal neurons coexpressing GPI-EGFP and synaptophysin–red fluorescent protein (RFP) before and during 600-AP, 40-Hz stimulation. During acquisition, we sparsely labeled surface GPI-EGFP with an extracellular anti-EGFP nanobody conjugated to ATTO647N and localized and tracked individual molecules frame by frame at synaptophysin-RFP–positive release sites. We accumulated these localizations to generate super-resolved images of filopodia-forming and control boutons (Fig. 7E) and analyzed individual trajectories to estimate membrane diffusion and directional displacement (Fig. 7, I and J). First, to determine whether lipid redistribution during filopodia formation exhibits the same directional bias as SypmOr2 redistribution measured above, we performed line profile analysis on super-resolved single-molecule localization maps acquired before and during filopodial extension (Fig. 7, E to H). We drew line profiles along the bouton axis from the distal synapse toward the filopodial base, normalized each profile within its respective condition, and subtracted baseline values to calculate a spatial lipid accumulation index (Fig. 7H). At baseline, GPI-EGFP distribution was broadly uniform along the bouton axis (Fig. 7, E and F). Upon stimulation, accumulation rose systematically toward the filopodial edge, peaking in the bins immediately adjacent to the filopodial base, while distal bins remained unchanged (Fig. 7, E, G, and H). The spatial gradient of GPI-EGFP accumulation thus mirrored the directed redistribution of SypmOr2, suggesting that stimulation-induced membrane flow toward the filopodium is not restricted to SV protein but extends to the bulk lipid bilayer.

To determine the velocity of this apparent directed displacement, we performed single-particle tracking (SPT) on GPI-EGFP trajectories at boutons with and without filopodia during high-frequency stimulation (Fig. 7, I and J). The time dependence of squared displacement distinguishes diffusive from convective motion: Pure diffusion produces a linear increase, whereas convective transport produces a quadratic increase. Our SPT analysis revealed that the squared displacement of a substantial proportion of particles increased quadratically with time (>80% of synaptic boutons), indicating directional movement consistent with membrane convection. We then fitted the squared displacement curves to estimate particle velocities at boutons with and without filopodia. The resulting distributions contained two kinetically distinct populations. All boutons contained a slow component (∼0.5 μm s^−1^), which likely reflects SV exocytosis and endocytosis, whereas a fast component (1.0 to 1.3 μm s^−1^) matched our model predictions (Fig. 6E). Notably, the fast convection component was substantially higher at filopodia-forming boutons (47%) than at those without (15%) (Fig. 7J). When we incorporated these measured flow parameters into the model, the simulations reproduced the increase in synchronous release at the expense of asynchronous release associated with filopodia formation (Fig. 7K). The experimentally measured fast component fell within the range of 1 μm s^−1^ that the model had independently predicted was necessary to reproduce the observed shift toward synchronous release. This agreement between theory and experiment supports filopodia-driven convective membrane flow as a candidate mechanism by which presynaptic terminals clear release sites during sustained activity, linking structural dynamics at the bouton surface to short-term plasticity of neurotransmitter release.

## DISCUSSION

Here, we report that presynaptic filopodia couple structural plasticity to neurotransmitter release efficacy through changes in membrane mechanics. Filopodia are essential during neuronal development (*44*), where their exploratory behavior guides synaptic partner selection and their stabilization defines the rate-limiting step of synaptogenesis (*45*, *46*). Here, we show that presynaptic filopodia also constitute a substrate for plasticity in mature neurons and circuits. By transiently extending during synaptic activity, they enable boutons to contact multiple postsynaptic targets, forming short-lived neuronal kinapses reminiscent of the brief interactions between T cells and antigen-presenting cells in the immune system (*29*). Some filopodia persist after stimulation, potentially maturing into stable synapses or collateral branches. In the future, it will be important to determine how specific molecular pathways and patterns of activity instruct filopodial contraction, stabilization, and branch maturation and to understand how nascent axonal branches integrate into established networks to influence circuit function and behavior.

In addition to enhancing structural connectivity, filopodia formation modifies the mechanical properties of the presynaptic membrane, suggesting a biophysical mechanism through which structural remodeling influences neurotransmitter release kinetics. Reciprocal interactions between local biochemical signaling and long-range mechanical forces transmitted through the actin-membrane interface coordinate cellular behavior across diverse biological systems (*47*–*49*). In model membranes, increased bilayer tension alters lipid order and fluidity, enhancing lateral diffusion by up to 50% (*50*). Membrane protrusion and contraction can each generate directed membrane flow toward the site of deformation (*13*), redistributing membrane molecules and establishing local polarity (*15*). At the presynapse, clearance of SV components after exocytosis, a process normally limited by diffusion (*42*), is essential to relieve short-term synaptic depression (*41*). Our data, together with mathematical modeling, support a model in which filopodia-driven membrane flow substantially accelerates this clearance to sustain *Pr*.

Maintaining *Pr* during sustained activity is critical because, as *Pr* declines, a greater fraction of SVs bypass rapid synchronous fusion within milliseconds of an AP and instead fuse after a delay (*51*, *52*). We find that sustained synchronous release rate inversely correlates with posttrain asynchronous release frequency at individual synapses, supporting the conclusion that synchronous and asynchronous release draw SVs from the same pool (*33*). Therefore, our experimental and modeling data implicate filopodia formation in regulating synaptic release mode, coupling release kinetics to the presynaptic mechanical state.

Although factors additional to filopodia formation could, in principle, contribute to the observed shift in release kinetics, several converging observations argue against the possibility that filopodia are merely a correlate of the functional change. First, filopodia-forming and nonforming boutons on the same axon were functionally indistinguishable at stimulus onset. Their initial release rate, variability, and individual fusion event parameters were equivalent, arguing against a preexisting high-*Pr* subpopulation. Release kinetics instead diverged progressively as filopodia formed, consistent with structural remodeling contributing to the functional change. Second, perturbations of NHE activity, including NHE1 knockdown, EIPA, and amiloride, each reduced activity-dependent filopodia. NHE proteins regulate actin-dependent membrane protrusions in epithelial cells and neuronal growth cones (*36*–*38*), providing a mechanistic basis for this effect. The effects of EIPA on release kinetics were restricted to sustained high-frequency stimulation, when filopodia form, without changes in basal synaptic transmission, presynaptic calcium, bouton ultrastructure, or SV pool architecture. Last, our mathematical model independently predicted that an in-plane convective flow rate of ∼1 μm s^−1^ is required to reproduce the observed shift from asynchronous to synchronous release, and SPT at filopodia-forming boutons revealed a fast membrane displacement component within this range. Therefore, the quantitative agreement between prediction and measurement supports a model in which filopodia-associated membrane remodeling promotes release-site clearance and maintains synchronous neurotransmission during sustained activity. Nevertheless, current tools act at the whole-neuron level. Future studies using approaches that control filopodia formation at individual boutons with the temporal precision of synaptic transmission would strengthen our conclusions.

By forming kinapses and altering neurotransmitter release kinetics, filopodia may contribute to the long-term rewiring of synaptic circuits. Spontaneous neuronal activity before critical periods and sensory-driven activity during and after them shape neural circuit refinement (*11*, *53*, *54*). This process depends on competition among neighboring neurons and synchronization among afferents or between afferents and postsynaptic targets (*55*). At the single-cell level, the selection and stabilization of individual connections follow Hebbian plasticity rules (cells that fire together, wire together) (*56*–*58*). Our findings raise the possibility that filopodia participate in a presynaptic mechanism compatible with Hebbian plasticity. By forming kinapses with the same or neighboring neurons, a single bouton expands the pool of candidate contacts available for activity-dependent selection. SVs redistribute into, and undergo fusion within, a subset of filopodia, indicating that these protrusions can support neurotransmitter release at sites displaced from the original postsynaptic target. Contacts with the same neuron could strengthen an existing connection, whereas contacts with another neuron could transiently broaden presynaptic output and recruit additional cells into the active network. The enhanced synchronous release observed at filopodia-bearing boutons could also promote temporally precise postsynaptic activation and thereby facilitate the stabilization of nascent contacts during sustained or correlated activity. Presynaptic filopodia may therefore function as dynamic structural intermediates through which neurons sample their local environment and, when activity is sufficiently correlated, seed stable new connections. Although this interpretation remains to be tested directly, it provides a parsimonious link between presynaptic structural remodeling, release kinetics, and established principles of activity-dependent synaptic refinement.

In summary, our results reveal that neuronal activity dynamically remodels synaptic connectivity and that the accompanying changes in membrane mechanics actively shape presynaptic function and plasticity. Determining whether activity-induced presynaptic filopodia support adaptive circuit plasticity or contribute to pathological circuit reorganization will be an important direction for future work.

## MATERIALS AND METHODS

### Animal use

Neuronal cells and brain tissue were obtained from Thy1-GFPM and wild-type C57BL/6J mice. C57BL/6J mice were sourced from Charles River Laboratories, while Thy1-GFPM mice were obtained from the Jackson Laboratory (strain no. 007788, RRID: IMSR_JAX:007788). Homozygous and hemizygous Thy1-GFPM mice show EGFP expression in sparse subsets of neurons (10% of cortical neurons). Both homo- and hemizygous animals were bred to maintain the mouse colony. Mouse colonies were housed in individually ventilated cages, all maintained on a 14-hour light/dark cycle (light from 7:00 to 21:00). Breeding animals were fed RM1 chow, and stock animals were maintained on RM3 chow.

All animal procedures adhered to the UK Animal (Scientific Procedures) Act 1986, under Project and Personal License authorization, and received approval from the Animal Welfare and Ethical Review Body at the University of Edinburgh (Home Office project licenses to M.A.C., PP5745138, and A.G.-S., PP1538548). All euthanasia procedures were performed using schedule 1 protocols in accordance with UK Home Office Guidelines. Euthanasia of adult animals was performed through either cervical dislocation or exposure to rising CO_2_ levels with death confirmed by decapitation, while embryos were euthanized via decapitation with death confirmed by brain destruction. All procedures for the use and care of mice for EM experiments were authorized by the Animal Welfare Committee of Charité Medical University and the Berlin State government.

### Materials

In this study, the following materials were used: APV (ab120271), CNQX (ab120044), 4-AP (ab120122), amiloride hydrochloride (ab120281), and SMIFH2 (ab218296) from Abcam; bafilomycin A1 (A8627) and EGTA AM [10 mM dimethyl sulfoxide (DMSO) solution; 21006] from Stratech; papain (LK003176) from Worthington; trypsin-EDTA from Thermo Fisher Scientific (15400-054); EIPA (B7389) from ApexBio; dynasore (2897) from Tocris; TeNT from Calbiochem (582243-25UG); Neurobasal (21103-049), l-glutamine (25030-024), B27 supplement (17504-044), penicillin-streptomycin (15140-122), Fura Red AM cell permeant (F3021), Pluronic F-127 (P6867), and Lipofectamine 2000 (11668-019) from Thermo Fisher Scientific; fetal bovine serum (FBS; S1810-500) from Biosera; FluorSave reagent (345789) from Merck Millipore; poly-d-lysine hydrobromide (P7886), poly-l-lysine (P1149), Laminin (L2020), 3,3′-diaminobenzidine (D8001), glutaraldehyde grade I (G5882), Hanks’ balanced salt solution (HBSS; H9394), and protease inhibitor cocktail (P8849) from Sigma-Aldrich; and osmium tetroxide (RT19140), crystalline (E19132), and acetone (RT100016) from Electron Microscopy Services.

### Oligonucleotides

The following oligonucleotides were used to genotype Thy1-GFPM mice: ACA GAC ACA CAC CCA GGA CA (transgene forward), CGG TGG TGC AGA TGA ACT T (transgene reverse), CTA GGC CAC AGA ATT GAA AGA TCT (positive control forward), and GTA GGT GGA AAT TCT AGC ATC ATC C (positive control reverse).

### DNA constructs

The following expression vectors were used in this study and were obtained from the indicated sources: SypHy, L. Lagnado (University of Sussex); Syp-mOr2, S. Takamori (Doshisha University, Kyoto, Japan); RFP-Bsn, C. Garner (German Centre for Neurodegenerative Diseases, Berlin); pAAV.hSyn-synaptophysin-miRFP670 (short synaptophysin-RFP) and pLenti_GPI-6His-GFP were cloned in the laboratory of M. Heine (Johannes Gutenberg University, Mainz); pCAG:GPI-GFP (Addgene plasmid # 32601, http://n2t.net/addgene:32601, RRID: Addgene_32601) (*59*); GPI-mApple (Addgene plasmid # 182868, http://n2t.net/addgene:182868, RRID: Addgene_182868) (*60*); mRuby-Lifeact-7 (Addgene plasmid # 54560, http://n2t.net/addgene:54560, RRID: Addgene_54560); pAAV.hSyn.iGluSnFR3.v857.SGZ (Addgene plasmid # 178330, http://n2t.net/addgene:178330, RRID: Addgene_178330) (*31*); GCaMP6f (Addgene plasmid # 40755; RRID: Addgene_40755) (*61*); and an shRNA-targeting mouse NHE1 (*mSlc9a1*), target sequence CCACAATTTGACCAACTTAAT, was cloned into a lentiviral vector, pLV[shRNA]-EGFP-U6 > mSlc9a1[shRNA#2], VB900142-8600taz, by VectorBuilder. The scrambled control plasmid (sequence GACTTTACTGCCCCTTACT) was from (*62*).

### Antibodies

The following primary mouse antibodies were used in this study: synaptobrevin 2 [Western blotting (WB); 1:2000; Synaptic Systems, 104211, RRID:AB_2619757], GluA Oyster550 [immunocytochemistry (ICC); 1:200; Synaptic Systems, 182411C3, RRID: AB_2619877], and GFP-Booster_ATTO647N (uPAINT; 1:50,000; ChromoTek, gba647n-100, RRID: AB_2629215). The following rabbit antibodies were used: synaptophysin 1 (WB; 1:1000; Synaptic Systems, 101 002, RRID: AB_1961591), SV2A (ICC; 1:1000; Abcam, ab32942, RRID: AB_778192), vGAT sulfocyanine 3 (vGAT-Cy3) (ICC; 1:100; Synaptic Systems, 131103C3, RRID: AB_2189809), and NHE1 (ICC; 1:200; Thermo Fisher Scientific, PA520444, RRID: AB_11153959).

The following secondary antibodies were used in this study: donkey anti-rabbit Alexa Fluor 568 (ICC; 1:1000; Thermo Fisher Scientific, A10042, RRID: AB_2534017), goat anti-rabbit Alexa Fluor 488 (ICC; 1:1000; Thermo Fisher Scientific, A11008, RRID: AB_143165), goat anti-rabbit Alexa Fluor 647 (ICC; 1:1000; Thermo Fisher Scientific, A21244, RRID: AB_2535812), donkey anti-rabbit immunoglobulin G (IgG) IRDye 800 CW (WB; 1:10,000; LI-COR, 92532213, RRID: AB_2715510), and goat anti-mouse IgG IRDye 800 CW (WB; 1:10,000; LI-COR, 92632210, RRID: AB_621842).

### Tissue culture and transfection

Primary dissociated hippocampal neurons were prepared from mouse embryos [C57BL/6J; embryonic day 17.5 (E17.5)] or neonates [postnatal day 0 (P0) to P2]. Hippocampi were dissected in Dulbecco’s phosphate-buffered saline (PBS) and enzymatically dissociated with papain (10 U ml^−1^) at 37°C. Following quenching in minimum essential medium (MEM) containing 10% FBS, tissue was gently triturated to obtain a single-cell suspension. For iGluSnFR3.v857 (Fig. 4) and uPAINT experiments (Fig. 7), tissue was rinsed three times in cold HBSS and incubated in 1× trypsin-EDTA for 15 min before trituration.

Cells were plated on 25- or 18-mm glass coverslips precoated with either poly-d-lysine plus laminin or with poly-l-lysine alone (for the conditions used for Fig. 4). Neurons were maintained in Neurobasal medium supplemented with 2% B-27, 0.5 mM l-glutamine, and 1% (v/v) penicillin-streptomycin. To suppress glial proliferation, 1 μM cytosine β-d-arabinofuranoside was added at 3 days in vitro (DIV).

#### Transfection (lipid-based)

At DIV 7 to 8, neurons were transfected or cotransfected with the indicated plasmids using Lipofectamine 2000 (Thermo Fisher Scientific, 11668-019) according to the manufacturer’s instructions.

#### Transfection for iGluSnFR3.v857 and uPAINT experiments (calcium phosphate)

For experiments in Fig. 7, neurons were transfected at DIV 4 using calcium phosphate precipitation [as described previously (*63*)]. For each coverslip, 30 μl of sterile double-distilled H_2_O (ddH_2_O) was mixed with 3.8 μl of 2 M CaCl_2_ and 1.0 μg of each plasmid DNA. While gently vortexing, 30 μl of 2× Hepes-buffered saline was added dropwise, and the mixture was incubated for 15 min at room temperature to allow precipitate formation. Culture medium was replaced with prewarmed (37°C) Opti-MEM immediately before transfection. A total of 60 μl of the DNA–calcium phosphate suspension was applied per coverslip and incubated at 37°C for 30 min, after which cells were washed twice with Neurobasal medium and returned to their original conditioned medium for continued culture. Imaging of neurons occurred from DIV14 to 21.

For EM, primary hippocampal neurons from P0 to P2 C57BL/6J mice of either sex were cultured on astrocyte feeding layers aged 1 to 3 weeks, which were grown on sapphire discs. To inhibit glial proliferation, 8 μM 5-fluoro-2-deoxyuridine and 20 μM uridine were added before neuron culturing. Neurons were maintained in Neurobasal-A medium supplemented with B-27 and GlutaMAX (Thermo Fisher Scientific, 35050061) at 37°C for 14 to 21 days before analysis.

### Live imaging of filopodia formation and colocalization analysis with SypmOr2, LifeAct, Bsn, and GluA

High-resolution live imaging of synaptic membrane architecture during SV fusion was performed using a Nikon A1R or Nikon AXR-NSPARC (Fig. 2K and fig. S3) confocal microscopes, controlled by NIS Elements software. The microscopes were equipped with a resonant scanner for high-speed imaging and a live-cell imaging chamber with an environmental control unit, ensuring precise regulation of temperature and CO_2_ levels.

Primary hippocampal neurons were cotransfected with GPI-EGFP to label the plasma membrane and either SypmOr2, a reporter of SV recycling, or synaptophysin-RFP, a general presynaptic marker. Sparse transfection enabled imaging of individual presynaptic boutons. To monitor actin dynamics, neurons were cotransfected with GPI-EGFP and LifeAct-Ruby. For colocalization analysis with Bsn, neurons were cotransfected with GPI-EGFP and Bsn-RFP. For colocalization analysis with postsynaptic structures and inhibitory presynapses, GPI-EGFP–transfected hippocampal neurons were live labeled with either GluA-Oyster550 or vGAT-Cy3 antibodies for 20 to 30 min before imaging.

All live imaging was performed at 37°C in a buffer containing 136 mM NaCl, 2.5 mM KCl, 2 mM CaCl_2_, 1.3 mM MgCl_2_, 10 mM glucose, and 10 mM Hepes (pH 7.4), 50 μM APV, and 10 μM CNQX. For electrical field stimulation, cells were mounted in an imaging chamber (Warner Instruments, USA, RC-21BRFS) equipped with parallel platinum wires spaced 6 mm apart. The neurons were subjected to several stimulation protocols, including 600 APs (100 mA and 1-ms pulse width) at 40 Hz; 400 APs at 40 Hz; 600 APs at 5 Hz; 40 APs at 20 Hz; and 300 APs at 20 Hz. Z-stacks with a Z-step of 0.125 μm were acquired every 3 to 4 s for 1.5 or 3.5 min. Typically, imaging began with a baseline period, followed by either 15 s (600 APs at 40 Hz) or 120 s (600 APs at 5 Hz) of stimulation and then an additional 1 min of imaging poststimulation. To assess the calcium and activity dependence of filopodia formation, neurons were imaged before, during, and after a short pulse of 500 mM sucrose (5 s). To assess the contribution of intracellular calcium to activity-dependent filopodia formation, cultures maintained under basal conditions were compared with cultures treated with 4-AP (100 μM), either alone or following a 15-min preincubation with EGTA AM (100 μM; Stratech) (Fig. 2K and fig. S3). EGTA AM remained present throughout 4-AP exposure. Images were acquired from all conditions at 0, 5, and 60 min. In 4-AP–treated cultures, 0 min corresponded to the addition of 4-AP.

A 60× Plan Apo VC/numerical aperture (NA) 1.4 oil objective was used for imaging. Confocal images were acquired at 512 pixels by 512 pixels with a 12-bit depth and an optical zoom of 8.0×, resulting in a pixel size of 0.05 μm. Images in Fig. 2K and fig. S3 were acquired at 7.0× optical zoom with a pixel size of 0.082 μm. Excitation of EGFP was achieved at 488 nm, while emission light was collected at 525/50 nm. Excitation for SypmOr2, synaptophysin-RFP, LifeAct-Ruby, and GluA-Oyster550 was performed at 561 nm, with fluorescence signal detection at 595/50 nm. All time-lapse series of image stacks were deconvolved with Huygens Professional version 23.04 (Scientific Volume Imaging, The Netherlands, https://svi.nl/), using the CMLE algorithm, with signal-to-noise ratio of 15, 100 iterations, and automatically estimated background value.

Imaging of filopodia formation during SV fusion triggered by short stimulation (40 APs at 20 Hz) was performed on an inverted Nikon Ti2 microscope. This microscope was equipped with Photometrics Kinetix back-illuminated scientific complementary metal-oxide semiconductor (sCMOS) camera and “Nikon D-LEDI” 4 light-emitting diode illumination and controlled by NIS Elements software. The integrated live-cell imaging chamber and a control unit allowed maintenance of a constant 37°C environment.

Primary hippocampal neurons were cotransfected with GPI-EGFP for membrane labeling and SypmOr2 to report SV recycling. Imaging duration was 1.5 min, including a baseline measurement, a 2-s stimulation period (40 APs at 20 Hz), and ∼75 s of poststimulation observation. A 60× Plan Apo/NA 1.42 oil objective was used for imaging. Images were acquired at a frequency of 0.5 Hz with a 12-bit depth. EGFP was excited at 475 nm and its emission detected at 520/30 nm, while SypmOr2 excitation occurred at 550 nm with emission detection at 641/75 nm. Automatic 2D deconvolution was performed on all time-lapse series using the built-in wizard in NIS Elements software.

The morphometric analysis of filopodia was performed on maximum intensity Z-projections obtained from time-lapse series of image stacks, with the exception of the dynamic 3D colocalization analysis of GluA and filopodia. To sharpen the cell boundaries, an unsharp mask filter (with a radius σ = 1 pixel and a mask weight of 0.6) was applied in ImageJ (*64*), before segmentation using the Otsu thresholding algorithm.

Morphological changes over time were extracted using the Image Calculator tool in ImageJ. This involved subtracting the binary image of a prestimulation time point from subsequent time points to highlight regions of change and generate filopodial masks. For each filopodium, the frame of maximal extension was identified, and shape metrics (Feret length and roundness) were computed from the corresponding masks.

To quantify the ratio of transient versus stable filopodia, we defined condition-specific transient criteria. For filopodia induced by electrical field stimulation, transient filopodia were those that emerged and fully retracted during the stimulation period or within 15 s after stimulation ended. For filopodia induced by 4-AP (in primary cultures and acute slices), transient filopodia were those that persisted for ≤40 s from their initial emergence at maximal extension. Filopodia not meeting the transient criteria were classified as stable.

For the colocalization analysis with synaptic markers, the segmentation of presynaptic filopodia at their maximum extension was performed as described above, with the segmented structures then being converted into binary masks. These masks were subsequently applied to the fluorescence channel associated with synaptic markers to evaluate their colocalization with filopodia. Following background subtraction, the fluorescence intensity of each synaptic marker (Bsn, vGAT, GluA, and LifeAct) was quantified within the filopodial masks. Colocalization was determined after binarization, using predefined intensity thresholds. The presence of synaptic markers at the boutons from which the filopodia branched was also assessed. This was done by measuring the marker intensity within a circular ROI (∼1 μm by 1 μm) centered on the axonal shaft from which the filopodia extended. The intensity threshold was selected to discriminate true signals from background noise, thereby reducing the incidence of false-positive results.

DiAna ImageJ plugin was used to perform dynamic 3D colocalization analysis of GluA and GPI-EGFP–labeled axonal segments during filopodia formation (*65*). Rectangular ROIs measuring 6.1 μm by 6.1 μm were cropped and centered around axonal segments where filopodia were observed. On deconvolved image stacks, 3D segmentation was performed using DiAna’s built-in segmentation algorithm with a uniform global intensity threshold applied to the prestimulation image stack (a single time point) and the stack at maximum filopodial length. This segmentation allowed assessment of the 3D spatial volume occupied by GluA and GPI-EGFP–labeled objects. The computation of the number of colocalized GluA-labeled objects and GPI-EGFP, along with the calculation of colocalized voxels between the two, was achieved through postthresholding analysis of the respective labeled images. The examples of 3D time-lapse series in Fig. 3G were generated using the shadow projection function in Imaris 10.1.0.

### Live imaging and analysis of SypHy

Imaging of SypHy was performed using an inverted Zeiss Axio Observer Z1 microscope, with an EC Plan-Neofluar 40× oil immersion objective (NA 1.3), and a Colibri 7 light-emitting diode light source (Zeiss). The microscope was equipped with a Zeiss AxioCam 506 camera and was controlled by Zeiss ZEN 2 software. Transfected neurons were imaged using a GFP filter (exciter range, 450 to 490 nm; beam splitter at 495 nm; emitter range, 500 to 550 nm), and images were acquired at 2 Hz.

Quantitative analysis of SV fusion kinetics and SypHy response amplitudes was performed in the presence of 1 μM bafilomycin A1 (Stratech). Data from the SypHy time-lapse series were analyzed using the Time Series Analyzer plugin for ImageJ. ROIs were set at active SypHy synaptic terminals, and the average change in fluorescence intensity was tracked over time. The threshold for responsive terminals was set at an average fluorescence intensity increase of ≥2% above baseline upon AP stimulation. Any necessary adjustments for photobleaching were made using a correction factor obtained from inactive terminals on the same coverslip. The fluorescence response amplitudes elicited by field stimulation were normalized to the baseline (0) and the second response amplitude (1). Rate of exocytosis was measured from a linear fit.

### High-pressure freezing and freeze substitution

High-pressure freezing (HPF) and freeze substitution (FS) were performed as described in (*66*). For HPF, neurons (DIV 14 to 21) grown on 6-mm carbon-coated sapphire glass disks were treated with specified compounds (DMSO as a vehicle, 50 μM EIPA, 1 mM amiloride, 30 μM SMIFH2 for 30 min, and 80 μM dynasore for 3 to 5 min) in an extracellular solution containing 140 mM NaCl, 2.4 mM KCl, 10 mM Hepes, 2 mM CaCl_2_, 4 mM MgCl_2_, and 10 mM glucose (pH adjusted to 7.3 with NaOH, 300 mOsm) in an incubator at 37°C. For cryofixation, sandwiches consisting of the cultured sapphire glass disk, a 100-nm spacer ring, another sapphire glass disk, and a 400-nm spacer ring and filled up with extracellular solution and the respective compound were assembled. They were electrically stimulated with 600 APs at 40 Hz at 37°C and cryofixed within 5 ms following stimulation using a Leica ICE high-pressure freezer.

The sapphire disks were then placed in cryovials with an FS solution consisting of 1% (w/v) osmium tetroxide, 1% (v/v) glutaraldehyde, and 1% ddH_2_O in anhydrous acetone, precooled to −90°C. FS was performed using a Leica Microsystems AFS2 device with the following temperature protocol: −90°C for 5 hours, ramping from −90° to −20°C over 14 hours (5°C hour^−1^), holding at −20°C for 12 hours, and then ramping from −20° to 20°C over 4 hours (10°C hour^−1^).

The samples were then washed four times for 15 min each with anhydrous acetone, stained with 0.1% (w/v) uranyl acetate in acetone for 1 hour, and washed again four times for 15 min each with anhydrous acetone, all at room temperature. Next, the samples were infiltrated with epoxy resin [42.8% (w/w) Epon, 31.2% (w/w) DDSA, and 26% (w/w) MNA; Fluka] by soaking for 2 hours in 30% Epon/acetone, 2 hours in 70% Epon/acetone, and overnight in 90% Epon/acetone. After a day, the sapphire disks were placed in embedding molds with a mixture of Epon and Araldite [26.3% (w/w) Epon, 18.7% (w/w) Araldite, 51.9% (w/w) DDSA, and 2.98% (w/w) BDMA; EMS] for polymerization at 60°C for 48 hours.

### TEM and analysis of 2D and 3D EM

For 2D EM, ultrathin sections (50 nm in thickness) were cut using an Ultracut UCT ultramicrotome (Leica) and collected on formvar-coated 200-mesh nickel or copper grids. Before imaging, the samples were poststained with 2% (w/v) uranyl acetate in ddH_2_O and Reynold’s lead citrate for enhanced contrast. The sections were then imaged using a JEOL JEM 1400Plus transmission electron microscope equipped with a OneView camera. Images were captured at 60 kV with a pixel size 0.28 nm by 0.28 nm. All imaging was performed blind to the experimental conditions to prevent bias.

Images from all groups per culture were pooled and randomized into stacks using a custom ImageJ macro. Between 70 and 100 images were acquired per group.

Structural features were labeled using line and selection tools in ImageJ. Data analysis was performed using custom ImageJ macros and Python scripts (*66*). The AZ was defined as the presynaptic membrane facing postsynaptic density. Docked SVs were those whose outer membrane contacted the AZ membrane. The distribution of cytosolic SVs was measured by calculating the shortest distance to the AZ membrane, and SV diameters were determined by fitting a circle to the outer membrane. Endosome-like structures were identified as spherical (diameter > 60 nm) or tubular structures with a single outer membrane and a clear lumen.

For 3D EM, bright-field images were captured of stimulated (600 APs at 40 Hz) HPF/FS samples and candidate regions of fine axons selected for TEM (Zeiss AxioImager Z2). Samples were then glued to a resin block, trimmed with blades and glass knives, and ribbons of serial 70-nm-thick ultrathin sections were cut (Leica Ultracut 7 ultramicrotome) and collected onto formvar/carbon-coated single slot copper grids. Grids were contrasted with Uranyless and 3% lead citrate before imaging. TEM images were acquired using a 12-sample carousel and annular scanning TEM detector housed inside a Zeiss Gemini 360 field emission scanning electron microscope. Serial images from three samples were used to find 11 filopodia on five axons and then to trace filopodia (split into 200-nm segments from the tip), parent axons, endosomes, vesicles, mitochondria, and filaments using ImageJ (TrakEM2 plugin). These tracings were used to create 2D images and 3D reconstructions and to measure segment volumes of filopodia and endosome volumes to calculate endosome fractions (percentage of segment volume). SVs were counted for each segment then expressed as a density of SVs per cubic micrometer volume of each segment.

The raw EM data presented in Fig. 3J were obtained from the publicly available EM dataset: H01 (*30*). The present study involved secondary analysis of the dataset and did not involve the acquisition of new human tissue. Ethical approval for tissue collection and informed consent from the participant were obtained as described in the original study. The Neuroglancer platform was used for visual exploration of the dataset, the EM ultrastructure, and segmentations. For 3D visualization (right in Fig. 3J), only axons containing synaptic boutons with putative filopodia, and their postsynaptic targets were rendered. Serial EM images from the same sample were analyzed to trace filopodia and synaptic organelles (SVs, endosomes, mitochondria, and endoplasmic reticulum) using the ImageJ TrakEM2 plugin, as described above. These tracings were then used to generate the 2D images and 3D reconstructions (middle) displayed in Fig. 3J.

### Imaging and analysis of filopodia formation and axonal calcium spikes

Imaging spontaneous filopodia formation and axonal calcium dynamics was performed with Nikon Ti2 microscope using the same settings as described above. Primary hippocampal neurons were cotransfected with GPI-Apple to label the membrane and GCaMP6f as a calcium sensor. Before imaging, neurons were treated with either 100 μM 4-AP or a combination of 50 μM APV and 10 μM CNQX and incubated for 30 min. Time-lapse imaging was performed for 5 min (0.5 Hz) in the presence of these inhibitors.

Analysis was performed in ImageJ. Circular ROIs (∼1 μm by 1 μm) were set around putative synapses showing filopodia formation. Within these ROIs calcium responses, reported by GCaMP6f fluorescence, were monitored over the 5-min period, concurrently with filopodia dynamics. Baseline fluorescence (*F*_0_) was defined as the 10th percentile of the raw fluorescence trace across the entire recording. This approach was chosen over first frames or prestimulus averaging because 4-AP induces near-continuous spontaneous firing, rendering early time points unreliable as a true resting baseline. Fluorescence signals were expressed as Δ*F*/*F*_0_ = (*F* − *F*_0_)/*F*_0_. GCaMP6f Δ*F*/*F*_0_ traces were aligned to the time of maximal filopodial extension (T-max) using a window spanning −55 to +25 s. For each cell, traces from multiple events were averaged to generate a single per-cell time course. To quantify calcium activity relative to filopodial dynamics, we computed three per-cell measures: (i) the mean Δ*F*/*F*_0_ over the full recording, capturing baseline activity under sustained firing conditions; (ii) the maximum Δ*F*/*F*_0_ within the −55- to 0-s pre–T-max window; and (iii) the Δ*F*/*F*_0_ at T-max. These measures were compared at the cell level.

For the histogram in Fig. 2I, calcium transients were analyzed at the level of individual events. To improve signal-to-noise ratio, Δ*F*/*F*_0_ traces were smoothed using a second-order filter (GraphPad Prism 10). Peaks were detected automatically in Origin (v10.1.0.178) using a median-based baseline, local maximum detection, and a predefined amplitude threshold. For each filopodium event with at least one detected calcium transient in the −55- to 0-s window, the lag was defined as the time difference between T-max and the last preceding calcium peak.

### Acute slice preparation, Fura Red AM dye loading and live imaging of EGFP-expressing presynaptic boutons in coronal brain sections from Thy1-GFPM mice

Coronal brain slices (350 μm) were prepared from Thy1-GFPM mice (8 to 10 weeks old of either sex). The brains were quickly transferred to chilled (2° to 5°C) carbogenated sucrose-modified artificial cerebrospinal fluid (ACSF) (86 mM NaCl, 1.2 mM sodium phosphate buffer, 2.5 mM KCl, 25 mM NaHCO_3_, 25 mM glucose, 50 mM sucrose, 0.5 mM CaCl_2_, and 7 mM MgCl_2_) for 2 min. Brains were subsequently sectioned in the same solution using a vibrating microtome (Leica, VT1200S). The brain sections were left to recover for 30 min at 37°C in carbogenated standard ACSF, containing 126 mM NaCl, 3 mM KCl, 1.2 mM sodium phosphate buffer, 25 mM NaHCO_3_, 15 mM glucose, 2 mM CaCl_2_, and 2 mM MgCl_2_.

Neuron loading with Fura Red AM was performed as previously described (*67*). A fresh stock solution of 20% Pluronic F-127 in DMSO was prepared using sonication. The calcium-loading solution was prepared by mixing Fura Red AM (30 μM) and Pluronic F-127 (0.065%) in carbogenated standard ACSF. The brain slices were covered with the calcium-loading solution and incubated at 37°C/5% CO_2_ for 45 to 60 min in a cell culture incubator. After incubation, the dye solution was replaced with fresh ACSF to prevent dye overload. The slices were then incubated with either 100 μM 4-AP or a combination of 50 μM APV and 10 μM CNQX for additional 30 min before live imaging for 5 min in the presence of the inhibitors.

Brain slices loaded with Fura Red AM were placed in an imaging chamber mounted on a Nikon A1R confocal microscope. Superficial cortical layers with sparsely EGFP-labeled axons and putative synaptic boutons were selected for imaging. Time-lapse imaging was performed at 35° to 37°C with 5% CO_2_, using a resonant scanner over a 5-min period. A 40× Plan Fluor/NA 1.3 oil objective was used for imaging. Image stacks (typically, 50 to 70 sections, spaced 0.125 μm apart) were acquired at a speed of ∼0.07 Hz. Confocal settings were adjusted to a 1024 pixel–by–1024 pixel resolution, 12-bit dynamic range, and 4.0 optical zoom, achieving a final pixel size of 0.04 μm. Dual excitation of EGFP and Fura Red at 488 nm was implemented, with emissions collected at 525/50 nm for EGFP and 595/50 nm for Fura Red. The interaction of Fura Red with cytoplasmic calcium reduced its excitation efficiency at 488 nm, decreasing its emission at ∼650 nm.

All time-lapse series of image stacks were deconvolved with Huygens Professional version 23.04 (Scientific Volume Imaging, The Netherlands, http://svi.nl). A Gaussian filter with (σ = 4.0) was applied to the Fura Red channel in ImageJ to enhance the signal and minimize background noise. Synaptic boutons marked by Fura Red were inspected for fluorescence decreases, serving as a proxy for calcium responses through kymographs in ImageJ. The data used to generate kymographs were scaled multiple times to enhance visual clarity.

### iGluSnFR3.v857 and uPAINT SPT acquisition

Imaging for iGluSnFR3.v857 and uPAINT was performed on a Nikon Eclipse Ti 2 E inverted microscope equipped with an oil-immersion TIRF objective (60×, NA 1.45) expanded by a ×1.5 magnification lens, with the Perfect Focus System for *z*-axis stabilization. Samples were illuminated in TIRF mode. Image sequences were acquired either at 100 Hz (10-ms exposure, iGluSnFR3.v857; 3000 frames total) or at 20 Hz (50-ms exposure, uPAINT; 1000 frames total) using an sCMOS camera (Orca Flash 4.0, Hamamatsu Photonics; pixel size, 6.5 μm by 6.5 μm; sensor, 13.3 mm by 13.3 mm). Acquisition was controlled with NIS-Elements Advanced Research (Nikon).

For the uPAINT experiment, presynaptic boutons were identified by capturing synaptophysin-RFP–labeled release sites in a brief 50-frame time-lapse imaging sequence. Excitation was performed at 561 nm using a fiber-coupled Omicron laser combiner (Coherent; MPB Communications Inc.). After bouton detection, synaptophysin-RFP was photobleached by increasing the laser power shortly (1 min) to 100%. Subsequently, anti-EGFP ATTO647N nb (1:50,000) was added to the imaging solution to enable single-molecule tracking of membrane-bound GPI-EGFP ligands and extraction of particle trajectories.

During acquisition, fluorophores (iGluSnFR3.v857 and ATTO647N) were excited with 488- and 640-nm lasers operated at nonsaturating power (maximum 25% of initial laser power). A 1.5× lens in the TIRF coupling was used to reduce the pixel size to 0.072 μm by 0.072 μm, which optimizes the localization of single molecules. All imaging was performed at 37°C under continuous perfusion with imaging solution containing 145 mM NaCl, 2.5 mM KCl, 10 mM Hepes, 10 mM d-glucose, 2 mM MgCl_2_, 2 mM CaCl_2_, 10 μM APV, and 10 μM CNQX.

Neuronal stimulation was achieved via electrical field stimulation using two platinum electrodes placed on the coverslip, 15 mm apart. Pulses were generated by an isolated pulse stimulator, model 2100 (A&M Systems) and delivered through a stimuli isolator unit (WPI, A385). Individual pulses had a duration and current of 0.5 ms and 50 mA, respectively.

### Analysis of iGluSnFR3.v857 synchronous and asynchronous release responses

To resolve synchronous and asynchronous glutamate release events at individual presynaptic boutons, hippocampal neurons were sparsely transfected with iGluSnFR3.v857 (*31*), a genetically encoded glutamate sensor exhibiting rapid, nonsaturating activation kinetics. This sensor enables detection of synaptic glutamate release events with reduced signal saturation and enhanced specificity relative to extrasynaptic fluorescence in cultured neurons.

Neurons were stimulated with 300 APs at 20 Hz, a frequency selected to still resolve individual release events associated with each AP. Imaging was performed for 30 s total (4-s baseline, 15-s stimulation, and 11-s poststimulation). Synchronous release was quantified during the stimulation train, whereas asynchronous release was quantified after the last AP within the poststimulation window, where events are temporally uncoupled from action potentials.

To identify individual glutamate release sites along axons, fluorescence images were preprocessed in ImageJ using a Gaussian filter (σ = 2.0) to enhance the iGluSnFR3.v857 signal and reduce background noise. This preprocessing step improved the reliability of automated release site detection. Release sites at boutons, with or without filopodia, were automatically detected using a custom ImageJ macro based on the Find Maxima algorithm, which set circular ROIs (∼1 μm by 1 μm) centered on local fluorescence maxima. The detection threshold was optimized to minimize false-positive sites arising from background fluorescence.

Subsequent detection and classification of synchronous and asynchronous release events were performed in OriginPro (version 10.2.5.212) using the Peak Analyzer module. Baseline drifts induced by stimulation were corrected via the Asymmetric Least Squares Smoothing algorithm, followed by peak detection using the local maximum method. Peaks were filtered according to a uniform amplitude threshold applied across all experiments to ensure consistent detection sensitivity for both synchronous and asynchronous events.

The temporal dynamics of synchronous release were assessed by averaging the number of detected peaks per second, providing a measure of short-term plasticity. For quantitative analysis, the mean amplitude and mean frequency of asynchronous events were calculated.

Initial release rate was defined as the mean frequency of synchronous events detected within the first second of stimulation, calculated per bouton and averaged per cell. The properties of individual fusion events at train onset were characterized by extracting three parameters from the first detected peak at each bouton: peak response amplitude (Δ*F*/*F*_0_); FWHM, derived from the fitted peak profile as a measure of event duration; and delay to first response, defined as the latency from stimulus onset to the first threshold-crossing peak. Release variability at train onset was quantified using the CV of synchronous event frequency in the first second of stimulation, calculated per bouton as the standard deviation divided by the mean of AP-evoked event counts across that window. CV serves as an inverse proxy for initial *Pr*, with lower values indicating higher *Pr*.

To examine the relationship between sustained synchronous release and posttrain asynchronous release at the level of individual boutons, Pearson correlation coefficients were computed per cell between posttrain asynchronous event frequency and (i) synchronous release rate at the final second of the stimulation train; (ii) late-train release rate, defined as the mean event frequency across bins 12 to 15 s; and (iii) cumulative synchronous release summed across the full stimulation train. Correlations were performed per cell across all boutons on the same neuron regardless of filopodia classification.

### Analysis of membrane flow and uPAINT SPT analysis

To quantify directed redistribution of SV membrane protein during filopodia formation, two ROIs with identical size were placed on each bouton: one at the filopodial insertion point and one on the diametrically opposite, distal face. Raw SypmOr2 fluorescence was extracted from each ROI at 3-s intervals during electrical field stimulation (600 APs at 40 Hz; *t* = 3, 6, 9, and 12 s), and values were averaged per cell. Mean Δ*F* (*F* − *F*_0_) at the filopodial and distal ROIs was plotted across the stimulation time course as means ± SEM across boutons to visualize the divergence in raw fluorescence between the two compartments over time.

A normalized EI was computed at each time point as EI = (*F*_filo_ − *F*_distal_) / (*F*_filo_ + *F*_distal_), where positive values indicate progressive SypmOr2 accumulation toward the filopodial compartment, independent of absolute expression level. Net flux was quantified as the difference signal (*F*_filo_ − *F*_distal_) over time, with a linear regression used to extract the slope (Δ*F*/*s*) as the flux rate. As a control, the same analysis was applied to symmetrically placed ROIs on boutons that did not form filopodia.

To assess stimulation-induced redistribution of GPI-EGFP across the bouton surface, super-resolved single-molecule localization maps were generated using the ThunderSTORM plugin in ImageJ. Line profiles were drawn along the bouton axis from the distal part toward the filopodial insertion point on these maps. Bouton length was normalized by dividing each profile into 10 equal bins, allowing comparison across boutons of different sizes. The baseline profile (*F*_0_) was subtracted from the stimulation profile (*F*_stim_) at each bin to yield an intensity difference (%), isolating stimulation-driven changes in GPI-EGFP localization density from the baseline distribution.

For extracting particle velocities, image stacks for synaptophysin-RFP and GPI-EGFP–ATTO647N were processed in PALMTracer (*68*) for MetaMorph (v7.10.3.297). Presynaptic ROIs of identical size were defined for boutons with and without filopodia by briefly aligning the average synaptophysin-RFP frames to the maximum projection of the GPI-EGFP–ATTO647N frames. For both baseline (100 frames) and stimulation (300 frames) acquisitions, subpixel localization and trajectory reconstruction within each ROI were performed in PALMTracer. Spots were detected by image thresholding and localized by fitting a 2D Gaussian (9-pixel radius approximating the microscope point spread function; initial σ = 1.6), resulting in ∼40-nm localization precision in both *x* and *y*.

To calculate the membrane flow at synapses with and without filopodia, particle velocities were estimated from SPT squared displacement curves by fitting to the equation $\text{SD}={\left(\mathrm{vt}\right)}^{2}+4\mathrm{Dt}+c$, where SD is squared displacement in square micrometers, *D* is the diffusion coefficient in square micrometers per second, *v* is the particle velocity in micrometers per second, and *c* is a parameter to compensate for alignment error. To focus on particles undergoing appreciable flow and remove stubs/tracking errors, we only analyzed trajectories with an *r*^2^ fit greater than 0.7, trajectory length longer than six frames, and particle speed greater than 10^−5^ μm/s. Some particle trajectories showed a downward parabolic SD over time, suggesting reverse flow. To quantify these trajectories, we fit them to the equation $\text{SD}=-{\left(\mathrm{vt}\right)}^{2}+4\mathrm{Dt}+c$, with the same filtering steps. A total of 185 filopodia and 380 nonfilopodia trajectories were analyzed. This revealed two subpopulations of particles, one traveling at ∼0.5 μm/s present in all synapses and another traveling at ∼1 μm/s enriched in filopodia-containing synapses. The second subpopulation’s speed was attributed to filopodia formation and used in the membrane flow simulation to mimic the effect of filopodia.

### Protein biochemistry

For quantifying protein expression via WB, cell cultures treated with 2 nM TeNT or a vehicle for 24 hours were lysed in 2× SDS sample buffer. The buffer contained 67 mM tris (pH 7.4), 2 mM EGTA, 9.3% glycerol, 12% β-mercaptoethanol, bromophenol blue, and 67 mM SDS. The samples were boiled for 10 min before being separated using SDS–polyacrylamide gel electrophoresis and transferred to nitrocellulose membranes. The membranes were incubated with primary antibodies overnight at 4°C, followed by secondary antibodies for 1 hour at room temperature. Imaging was performed using an Odyssey 9120 Infrared Imaging System (LI-COR Biosciences) with LI-COR Image Studio Lite software. Analysis was done using ImageJ, measuring the integrated density of signals within identical rectangular ROIs set around the protein bands.

### Immunocytochemistry

For ICC, neurons were fixed in 4% PFA in PBS for 4 to 5 min at room temperature. Samples were blocked and permeabilized with 10% FBS, 0.1% glycine, and 0.3% Triton X-100 in PBS for 20 min and then incubated with primary antibodies overnight at 4°C. Secondary antibodies were applied for 1 hour at room temperature. Coverslips were mounted on microscope slides using FluorSave reagent (Merck Millipore). Immunofluorescence intensity was quantified in circular ROIs with a diameter of 1 μm, placed over nerve terminals in ImageJ.

Live labeling with GluA-Oyster550 or vGAT-Cy3 antibodies was performed by incubating neurons with fluorescently labeled antibodies dissolved in culture medium for 20 to 30 min at 37°C. For quantitative analyses, the same antibody solutions were applied to all coverslips in the corresponding experiments.

### Acute brain slice preparation for in vitro electrophysiology

Four- to 8-week-old C57BL/6J mice were used for all in vitro electrophysiology experiments. Brains were rapidly dissected and transferred to ice-cold sucrose-based ACSF bubbled with carbogen (95% O_2_ and 5% CO_2_). This consisted of 75 mM sucrose, 87 mM NaCl, 2.5 mM KCl, 25 mM NaHCO_3_, 1.25 mM NaH_2_PO_4_, 25 mM glucose, 7 mM MgCl_2_, and 0.5 mM CaCl_2_. Acute horizontal brain sections were then cut at 350 μm on a Leica VT1200S vibrating microtome. Slices were then allowed to recover at 37°C in a chamber filled with sucrose-based ACSF bubbled with carbogen for at least 30 min, before being transferred to a chamber containing ACSF consisting of 125 mM NaCl, 2.5 mM KCl, 25 mM NaHCO_3_, 1.25 mM NaH_2_PO_4_, 25 mM glucose, 1 mM MgCl_2_, and 2 mM CaCl_2_ at room temperature where they were maintained until required for experimentation.

### Electrophysiological recordings and data analysis

Before recording, slices were preincubated for 30 min with either 0.2% DMSO (used as a vehicle) or either 50 μM EIPA dissolved in 0.2% DMSO in ACSF. Slices were then transferred to a recording chamber and continually perfused with carbogenated ACSF at a flow rate of 2 to 3 min.

For whole-cell experiments, a bipolar stimulating electrode made from twisted nickel-chromium wire (80:20 ratio; 0.066 mm in width) was implanted into stratum radiatum at the boundary between CA1 and CA2. A glass micropipette (3 to 6 MΩ) was then filled with an internal recording solution consisting of 142 mM K-gluconate, 4 mM KCl, 0.5 mM EGTA, 10 mM Hepes, 2 mM MgCl_2_, 2 mM Na_2_ATP, 0.3 mM Na_2_GTP, 1 mM Na_2_-phosphocreatine, and 2.7 mM biocytin. Whole-cell configuration was achieved within a CA1 pyramidal neuron by use of negative pressure to rupture the cell membrane. Synaptic properties were tested in voltage clamp. Briefly, the amplitude of EPSCs was first tested in response to a series of single 50-μs stimulation pulses (100 to 10,000 μA). The stimulation intensity was then set to give an EPSC of ∼200 pA. Presynaptic properties were then examined through a tetanic stimulation protocol of 600 pulses at 40 Hz. This protocol was used to assess the plasticity of synchronous neurotransmitter release during repeated stimulation. All recordings were sampled at 20 kHz and acquired using Clampex v10.7 with an Axon Instruments Multiclamp 700B amplifier and Digidata 1550B digitizer.

All data were analyzed offline using Axograph X v1.8.0 or Clampfit v10.7. For synaptic properties, peak EPSC amplitude was measured for each stimulation pulse and normalized to the first pulse in the train. Asynchronous release frequency values were calculated by utilizing the template matching feature in Clampfit to detect all spontaneous excitatory currents in the first 0.8 s after the termination of the 40-Hz train.

### Statistical analysis

Statistical analysis was performed using GraphPad Prism 10. Before selecting statistical tests, all datasets were assessed for normality using the D’Agostino-Pearson omnibus test. Each data point in scatter plots represents the mean value from a single independent experiment (coverslip or slice), with “*n*” denoting the number of experiments and “*N*” denoting the number of tissue culture preparations or animals. Where relevant, paired analyses were used to account for within-neuron comparisons (e.g., within-neuron comparisons of boutons with versus without filopodia). For normally distributed data, results are expressed as means ± SEM, and group comparisons were made using two-tailed Student’s *t* tests (paired or unpaired as appropriate) or one-way analysis of variance (ANOVA), followed by Tukey’s or Dunnett’s post hoc correction for multiple comparisons. For non-normally distributed data, results are expressed as median ± interquartile range (IQR), and comparisons were made using the Mann-Whitney *U* test (two-tailed, unpaired) or Kruskal-Wallis test with Dunn’s post hoc correction for multiple comparisons. For experiments involving repeated measures across time, a repeated-measures ANOVA or a mixed-effects model with Sidak’s post hoc test was applied. For multigroup comparisons involving two independent variables, two-way ANOVA with Sidak’s or Dunnett’s post hoc test was applied to assess main effects and interactions. Correlations were assessed using Pearson’s correlation coefficient, with significance determined by a two-tailed test. Linear regression was used to quantify the net flux of SypmOr2 signal over the stimulation period, with the 95% confidence interval of the regression line reported. For one-sample tests assessing whether a measured variable differed significantly from a hypothesized value (Fig. 7, C and H, and fig. S12C), a one-sample *t* test was used. All tests were two-tailed unless otherwise stated. A significance threshold of *P* < 0.05 was applied throughout. Exact *P* values, test statistics, sample sizes, and summary statistics for all figures are provided in the figures, figure legends, and tables S3 and S4.

## Supplementary Materials

### The PDF file includes:

Supplementary Text Tables S1 to S4 Figs. S1 to S12 Legends for movies S1 to S6 References
