# Supplementary material for: Presynaptic filopodia form kinapses and promote convective membrane flow to sustain synchronous neurotransmitter release
Source: Sci Adv. 2026 Oct 28;12(44):eaei2661. doi: 10.1126/sciadv.aei2661 (PMC13715708; doi:10.1126/sciadv.aei2661)
Supplement: Supplementary file 1 [file sciadv.aei2661_sm.pdf]

Science Advances
AAAS

Supplementary Materials for

Presynaptic filopodia form kinapses and promote convective membrane flow to sustain synchronous neurotransmitter release

Akanksha Jain *et al.*

Corresponding author: Daniela Ivanova, d.ivanova@ed.ac.uk

Sci. Adv. **12**, eaei2661 (2026)
DOI: 10.1126/sciadv.aei2661

The PDF file includes:

Supplementary Text
Tables S1 to S4
Figs. S1 to S12
Legends for movies S1 to S6
References

Other Supplementary Material for this manuscript includes the following:

Movies S1 to S6

Supplementary Text

Model description

The model hypothesizes that filopodia generate a convective in-plane membrane flow that carries vesicular "debris" toward the peri-active zone. This clears out release sites in the AZ and enhances P_r, which promotes synchronous vesicle release.

To mathematically capture the above effect, the model consists of two parts. First, the model uses a 2D convection-diffusion equation on a simulated synapse to calculate the spatiotemporal evolution of vesicular debris at the AZ and peri-active zone. Second, based on the resulting spatial pattern at steady state, the model calculates P_r following an AP train, and how it impacts the associated synchronous and asynchronous releases (see below).

Convection-diffusion model

The simulation domain was subdivided into concentric circles representing the active and peri-active zones with radii of 0.15 μm and 0.3 μm, respectively. Exocytosis was assumed to be restricted to the AZ and dependent on the predicted P_r. Endocytosis was assumed to occur on the boundary of the peri-active zone, removing vesicular debris until the concentration returns to equilibrium. Membrane flow rates were assumed to be constant throughout the simulation. Our model parameters are constrained by 1) our measured membrane flow rate from the SPT experiment and 2) the existing measurements on protein diffusion and dimensions of the active zone and peri-active zone. The mathematical formulation is as follows:

$$\frac{\partial P}{\partial t} + \nabla \cdot (-D\nabla P + vP) = k_1 P_r(P) \mathbb{1}_{AZ} \quad (1)$$

$$-\mathbf{n} \cdot (-D\nabla P + vP)|_{\partial\Omega} = -k_2\left(max(P - P_0, 0)\right) \quad (2)$$

$$P_r(P) = \frac{P_{r(max)}}{1 + \left((max(P - P_0, 0)/C_0)\right)^{n_P}} \quad (3)$$

Below, the physical meaning of model variables and the nominal values of the model parameters are introduced. Importantly, the key model predictions are robust within physically realistic parameter ranges (see the Parameter Table S1). P is the dimensionless concentration of vesicular debris, P_0 is the equilibrium concentration of vesicular debris (equal to 1 by definition), $P_r(P)$ is the release probability with respect to vesicular debris concentration, $P_{r(max)}$ is the maximum release probability set to be 0.45, k_1 is the maximum rate of vesicular debris deposition due to exocytosis (10 $1/s$), k_2 is the turnover rate due to endocytosis (5 $\mu m/s$), n_p is the Hill Coefficient of particle-dependent inhibition (3), C_0 is the change in the concentration threshold before vesicle release is inhibited (0.15), v is the flow speed in μm/s, set to be 1 $\mu m/s$ with filopodia and 0 $\mu m/s$ without filopodia in the nominal case; D denotes the diffusion coefficient (0.02 $\mu m^2/s$), **n** denotes the positively oriented normal vector at the boundary, and $\mathbb{1}_{AZ}$ denotes the indicator function equal to 1 in the AZ and 0 elsewhere.

To solve the above equations (Eq. 1-3), COMSOL Version 6.3 was used with the MUMPS solver and a relative tolerance of 5×10^{-3}. The domain was meshed using a free triangular mesh with maximum mesh size 5×10^{-3} μm, minimum size 4.38×10^{-4} μm, maximum element growth rate 1.3, curvature factor 0.3, and resolution of narrow regions 1.

P_r calculation

The average simulated P_r in the AZ at steady state was then used as the representative P_r of synapses with or without filopodia. To calculate the effect of filopodia on synchronous and asynchronous vesicle release following an AP train, the representative release probabilities were input into a stochastic simulation of vesicle fusion. In the stochastic simulation, the synapse randomly underwent vesicle fusion or replenishment following a 1 ms AP at the start of the simulation. Release events occurring 10 ms after an AP were considered asynchronous releases, while those before were considered synchronous. This was mathematically formulated as:

$$\frac{dR}{dt} = -RP_rA(t) + k(R_0 - R) \quad (4)$$

$$A(t) = \begin{cases} 1 & t < t_{AP} \\ A_{sync}exp\left(-(t - t_{AP})/\tau_{sync}\right) + A_{async}exp\left(-(t - t_{AP})/\tau_{async}\right) & t \geq t_{AP} \end{cases} \quad (5)$$

With the stochastic formulation being:

$$\frac{dP(R,t)}{dt} = P(R-1,t)k(R_0 - R + 1) + P(R+1,t)(R+1)P_rA(t) - P(R,t)k(R_0 - R) - P(R,t)RP_rA(t) \quad (6)$$

Where R is the number of vesicles in the RRP, P_r is the maximum probability of vesicle release (0.44 1/ms with filopodia, 0.048 1/ms without filopodia), k is the rate of replenishment in the RRP (0.004 1/ms), R_0 is the maximum number of vesicles in the RRP (6 vesicles), A(t) is a function modeling the decay in P_r that occurs immediately after an AP, A_{sync}, A_{async} are the relative weights of synchronous and asynchronous release pathways respectively (0.95, 0.05), τ_{sync}, τ_{async} are the time constants for decay of synchronous and asynchronous release probabilities following an AP (5 ms, 100 ms), and t_{AP} is the length of an AP pulse in milliseconds (1 ms). To simulate RRP depletion after an extended AP train, the simulation was initialized with a half-full RRP. For references, see the Parameter Table S2. Again, key predictions are robust within realistic parameter ranges and initial conditions.

The master equation (Eq. 6) was sampled for 30000 trials using the inhomogeneous Gillespie Algorithm (*69*). Briefly, to calculate the next timestep, the time-dependent propensities of the replenishment and vesicle release reactions were first numerically integrated from the current timestep to the next timestep:

$$a_0(\tau) = \int_{t_0}^{t_0+\tau} \left(a_1 + a_2(t)\right)dt \quad (7)$$

$$a_1 = k(R_0 - R) \quad (8)$$

$$a_2(t) = -RP_rA(t) \quad (9)$$

The waiting time X_1 between the current time t_0 and the next reaction follows:

$$P(X_1 > \tau) = e^{-a_0(\tau)} \quad (10)$$

Implying:

$$P(X_1 \leq \tau) = 1 - e^{-a_0(\tau)} \quad (11)$$

Then to sample from this distribution, inverse transform sampling was used. Briefly, a random number U was drawn from the uniform distribution, and the value of τ was then solved from the equation $\int_{t_0}^{t_0+\tau}\left(a_1 +\right.$

$a_2(t)\big)dt = -ln(1-U)$ numerically. The time was updated using the calculated timestep, and the next reaction was chosen with probability $P(rxn_i) = a_i(t+\tau)/\big(a_1 + a_2(t+\tau)\big)$.

This algorithm was repeated until the time exceeded 50 ms, after which the next trial would start. After 30000 trials, the predicted proportion of synchronous and asynchronous fusion events was calculated. Vesicle fusion events that occurred within 10 ms of an AP were considered synchronous, while fusion events after 10 ms were considered asynchronous.

Parameter Table S1: Membrane flow model

Parameter	***Parameter Description***	***Measured values***	***Values used in Model***
k_1	Maximum rate of particle deposition	4.8-13 1/s [(*70*)]	10 $1/s$
k_2	Rate of particle sequestration	0.5-5 $\mu m/s$ [(*71*)]	5 $\mu m/s$
C_0	Exocytosis-inhibiting particle threshold	<0.6 [(*72-74*)]	0.15
v	Flow Speed	From data	1.0 $\mu m/s$ w/ filopodia, 0 $\mu m/s$ w/o filopodia
D	Diffusion Constant	0.01-0.1 $\mu m^2/s$ [(*75*)]	0.02 $\mu m^2/s$
n_p	Inhibition Hill Coefficient	>1 [(*73, 74, 76*)]	3
$P_{r(max)}$	Max Probability of vesicle release	0-0.87 [(*77, 78*)]	0.45

Parameter Table S2: Stochastic vesicle release model

Parameter	***Parameter Description***	***Measured values***	***Values used in Model***
P_r	Probability of vesicle release during AP	Calculated from membrane flow model	0.44 1/ms w/ filopodia, 0.048 1/ms w/o filopodia
R_0	Maximum capacity of RRP	5-12 [(*26, 78, 79*)]	6
k	RRP replenishment rate	0.001-0.004 1/ms [(*76, 79, 80*)]	0.004 1/ms
A_{sync}	Synchronous pathway weight	>0.9 [(*32*)]	0.95
A_{async}	Asynchronous pathway weight	<0.1 [(*32*)]	0.05
τ_{sync}	Synchronous pathway time constant	1-5 ms [(*32*)]	5 ms
τ_{async}	Asynchronous pathway time constant	10-500 ms [(*32*)]	100 ms
t_{AP}	Length of AP	1 ms stimulation used in exp.	1 ms

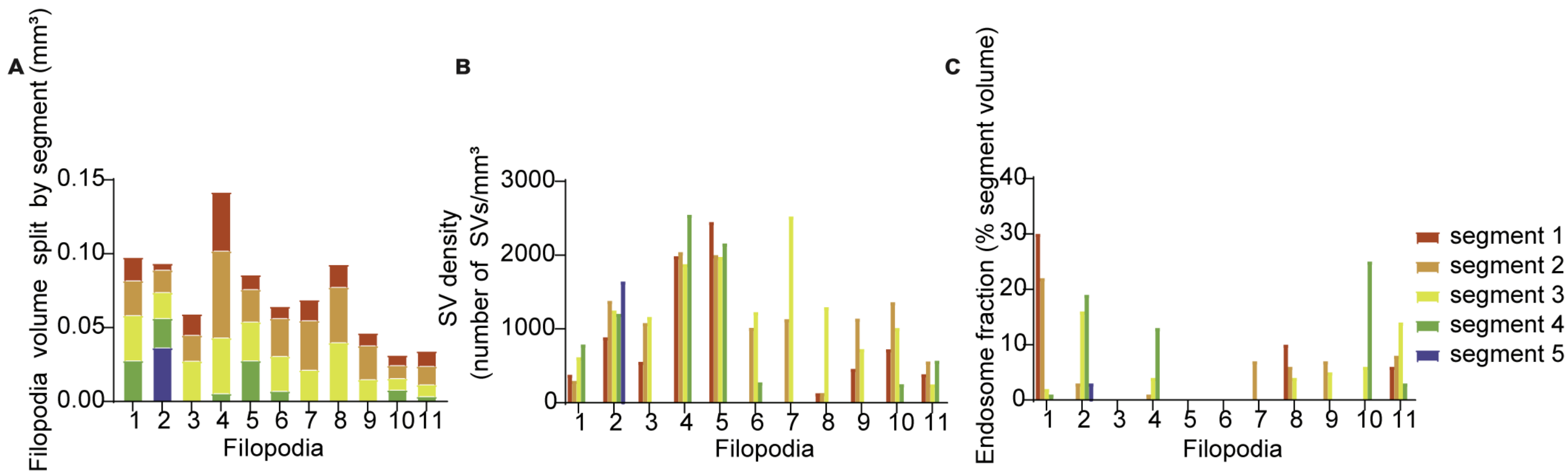

Fig. S1. Ultrastructural volumetric analysis of presynaptic filopodia. (A) Segment volumes of eleven filopodia, color-coded as in Fig. 1G. Filopodia under 800 nm lack segment 5; under 600 nm lack segments 4 and 5. **(B)** Segment SV densities (number of SVs per µm³). **(C)** Endosome fraction of filopodia segments (percentage of segment volume).

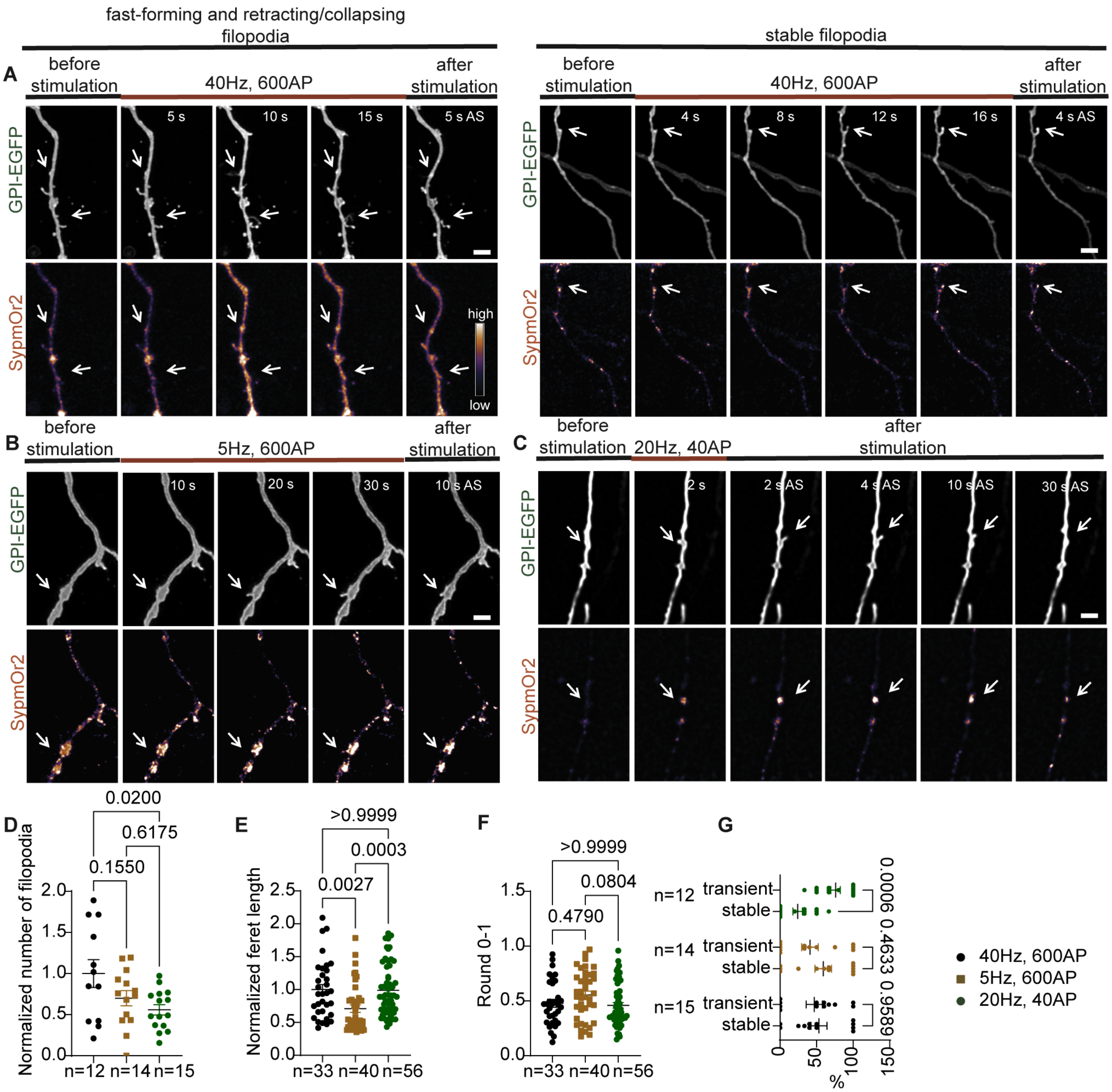

Fig. S2. Presynaptic filopodia form in response to diverse stimulation paradigms. (A) Confocal time-lapse series of boutons displaying fast-forming and contracting filopodia (left) or stable filopodia (right) in response to 600 AP at 40 Hz. **(B)** Confocal time-lapse series of boutons displaying filopodia formation in response to 600 AP at 5 Hz. **(C)** 40 AP at 20 Hz. **(A to C)** White arrows, filopodia-forming boutons. AS, after stimulation. Scale bars, 1 μm. **(D)** Normalized filopodia number per 10 μm of axon length for 600 AP at 40 Hz, 600 AP at 5 Hz, and 40 AP at 20 Hz. Lines are the mean, error bars are SEM. P values, one-way ANOVA with Tukey's test. **(E)** Normalized filopodia length. Lines are the median, error bars are IQR. P values, Kruskal-Wallis with Dunn's test. **(F)** Quantification of filopodial shape based on roundness. Lines are the median, error bars are IQR. P values, Kruskal-Wallis with Dunn's test. **(G)** Ratio of stable vs transient filopodia. Lines are the mean, error bars are SEM. P values, two-way ANOVA with Sidak's test.

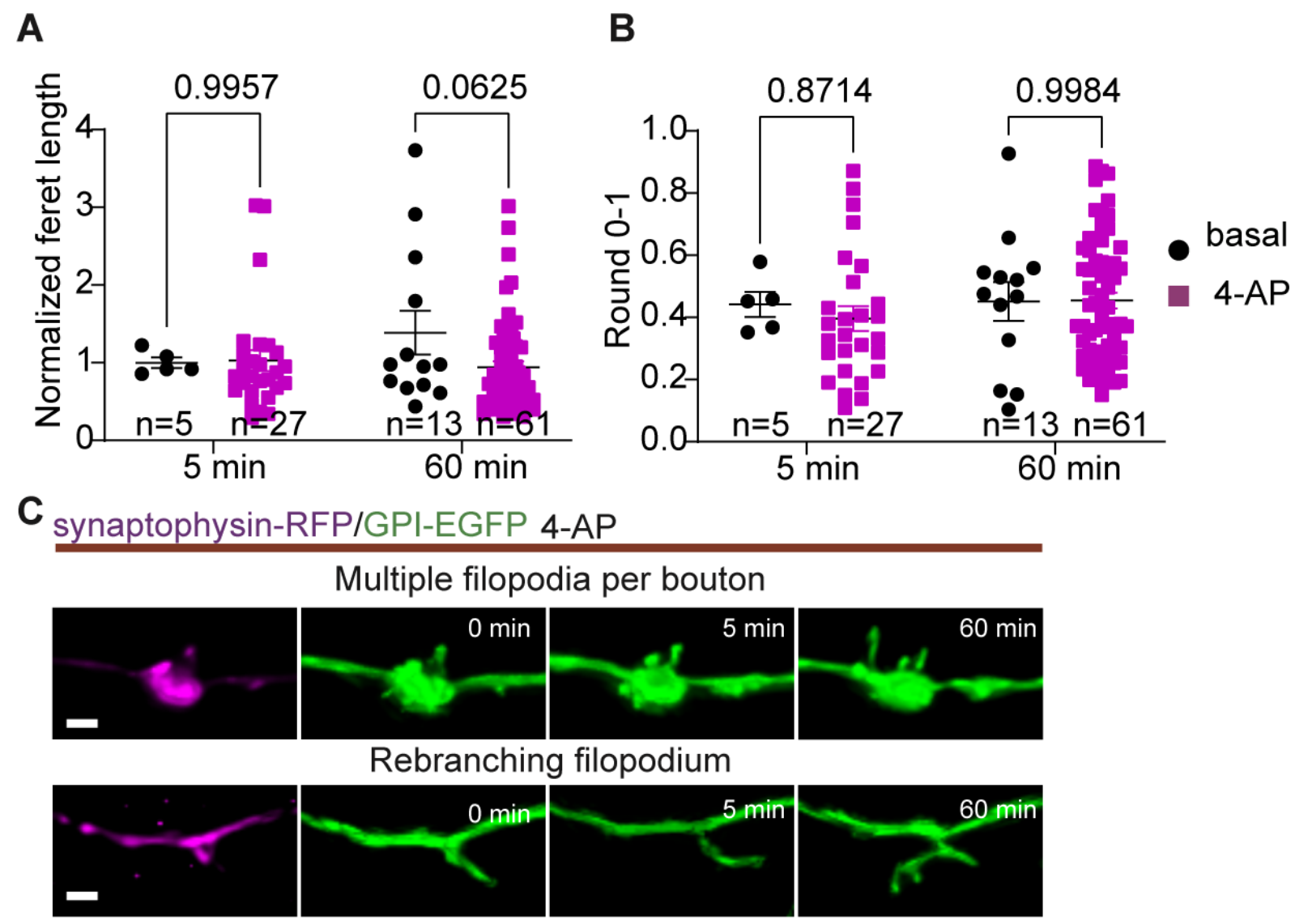

Fig. S3. Presynaptic filopodial length and morphology during basal and 4-AP-enhanced activity. (A, B) Normalized filopodial length **(A)** and shape **(B)** under basal conditions or following 4-AP treatment, measured at 5 and 60 min. Lines are the mean, error bars are SEM. P values, two-way ANOVA with Sidak's test. **(C)** Representative images showing multiple filopodia arising from a single synaptic bouton (upper row) and a rebranched collateral-like protrusion (lower row) following 4-AP treatment. Scale bars, 1 µm.

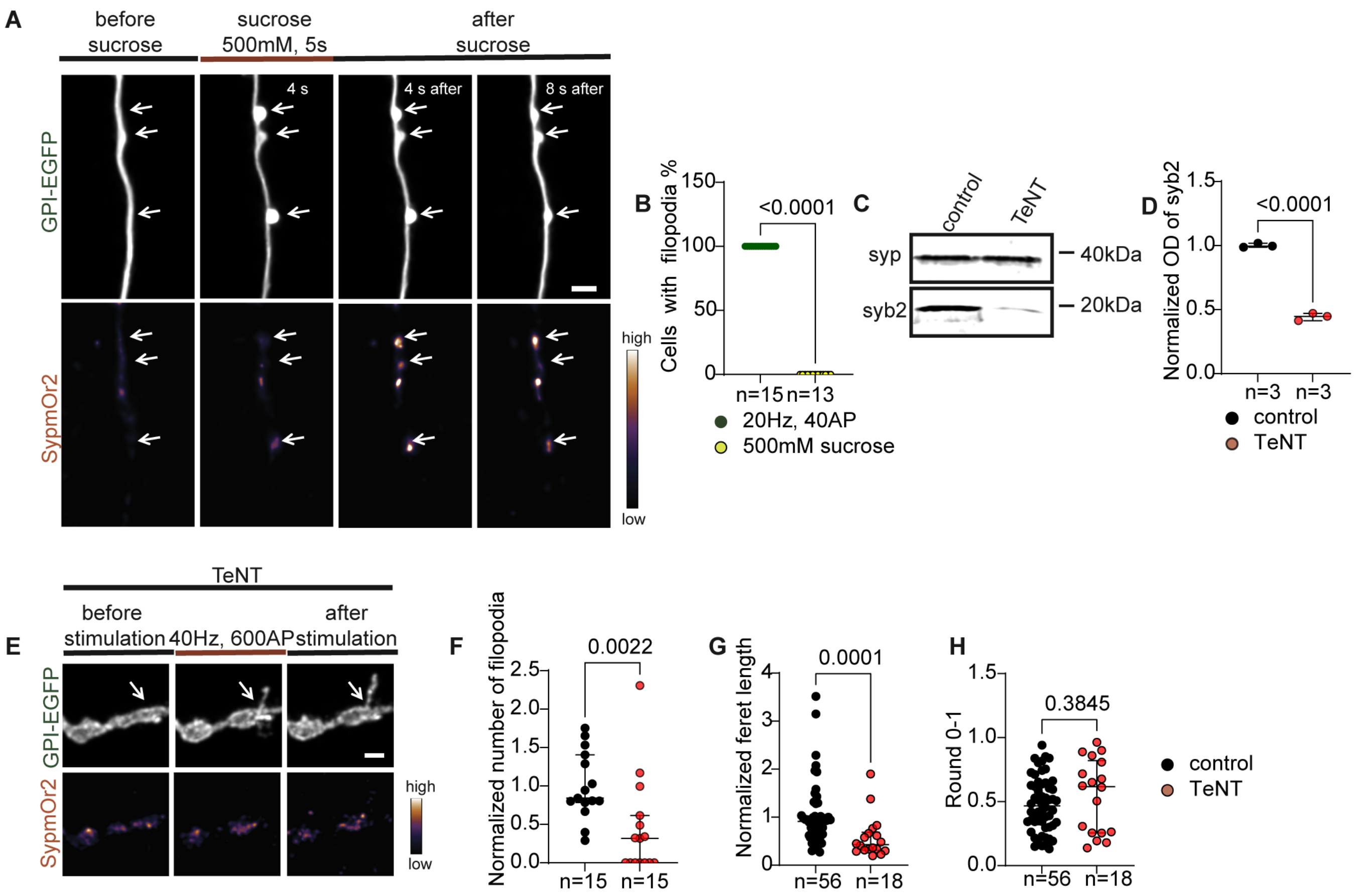

Fig. S4. Activity-induced presynaptic filopodia formation does not require SV fusion. (A) Primary hippocampal neurons were co-transfected with GPI-EGFP and SypmOr2. Neurons received a 5-s hypertonic sucrose pulse (500 mM). A representative time-lapse series shows sucrose-triggered SypmOr2 fluorescence responses and structural changes at synapses (white arrows indicate presynaptic enlargements and SypmOr2 responses). Scale bar, 1 µm. **(B)** Quantification of number of filopodia after 40 AP at 20 Hz or a sucrose pulse, both mobilizing RRP. Lines are the median, error bars are IQR. P values, Mann-Whitney U test. **(C)** Neurons treated with 2 nM tetanus toxin (TeNT) or vehicle control for 24 h, then lysed. Representative western blots (WBs) with synaptobrevin2 (syb2) and synaptophysin (syp) antibodies. **(D)** WB analysis of syb2 expression levels normalized to syp. Lines are the mean, error bars are SEM. P values, unpaired t test. **(E)** Neurons treated with 2 nM TeNT for 24 h. Time-lapse series shows filopodia formation despite lack of SypmOr2 responses (white arrows, filopodia). Scale bar, 1 µm. **(F)** Normalized number of filopodia per 10 µm axon length in cultures with and without 2 nM TeNT for 24 h (stimulation: 600 AP, 40 Hz). Lines are the median, error bars are IQR. P values, Mann-Whitney U test. **(G)** Normalized filopodia length in cultures with and without 2 nM TeNT for 24 h (stimulation: 600 AP, 40 Hz). Lines are the median, error bars are IQR. P values, Mann-Whitney U test. **(H)** Quantification of filopodia shape (roundness) in cultures with and without 2 nM TeNT for 24 h (stimulation: 600 AP, 40 Hz). Lines are the median, error bars are IQR. p values, Mann-Whitney U test.

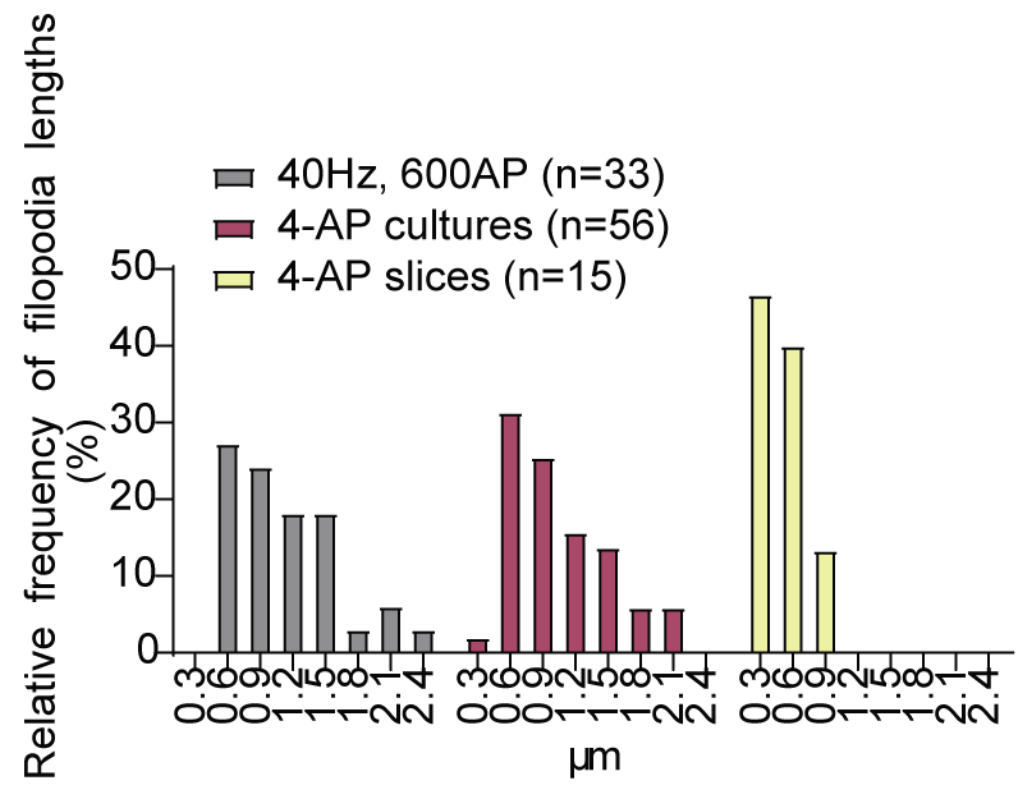

Fig. S5. Length of presynaptic filopodia in different neuronal preparations. Frequency histogram showing the length (µm) of presynaptic filopodia induced by electrical field stimulation (600 AP, 40 Hz) or 4-AP in dissociated neuronal cultures, and by 4-AP in acute brain slices.

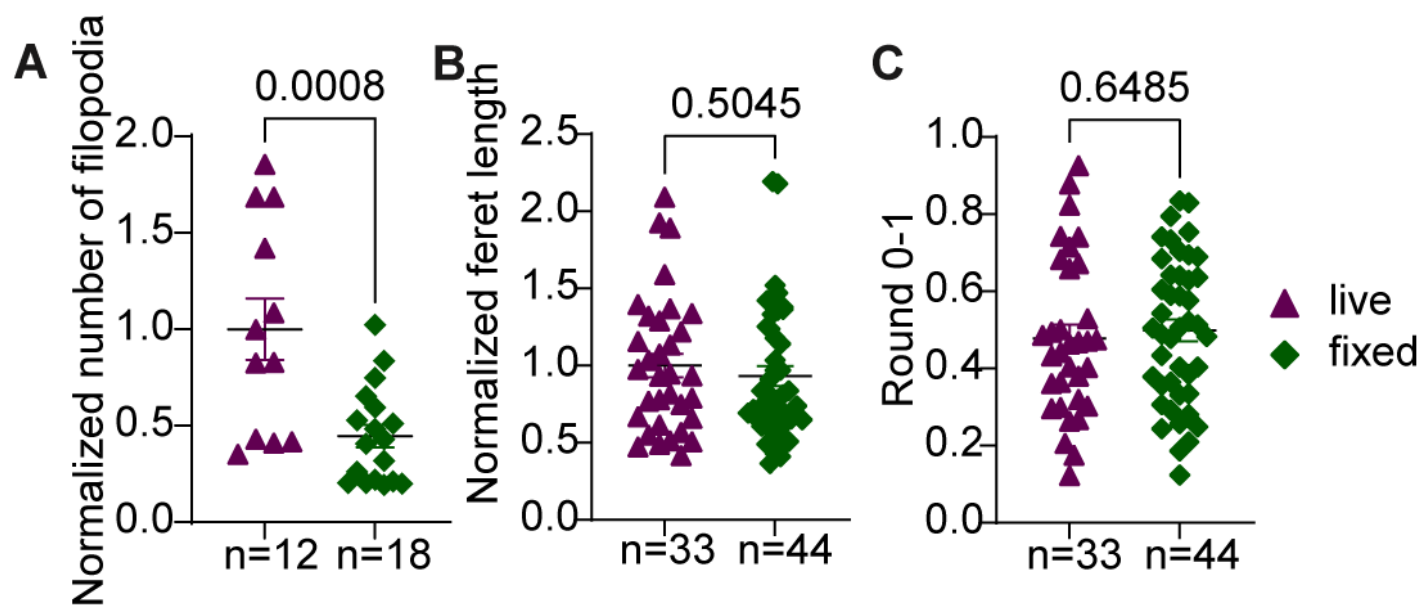

Fig. S6. Presynaptic filopodia number, length, and morphology in live and fixed neuronal cultures. (A to C) Comparison of normalized filopodia number per 10 µm of axon length **(A)**, normalized filopodial length **(B)**, and filopodial roundness **(C)** between live and fixed cultures. Lines are the mean, error bars are SEM. P values, unpaired t tests.

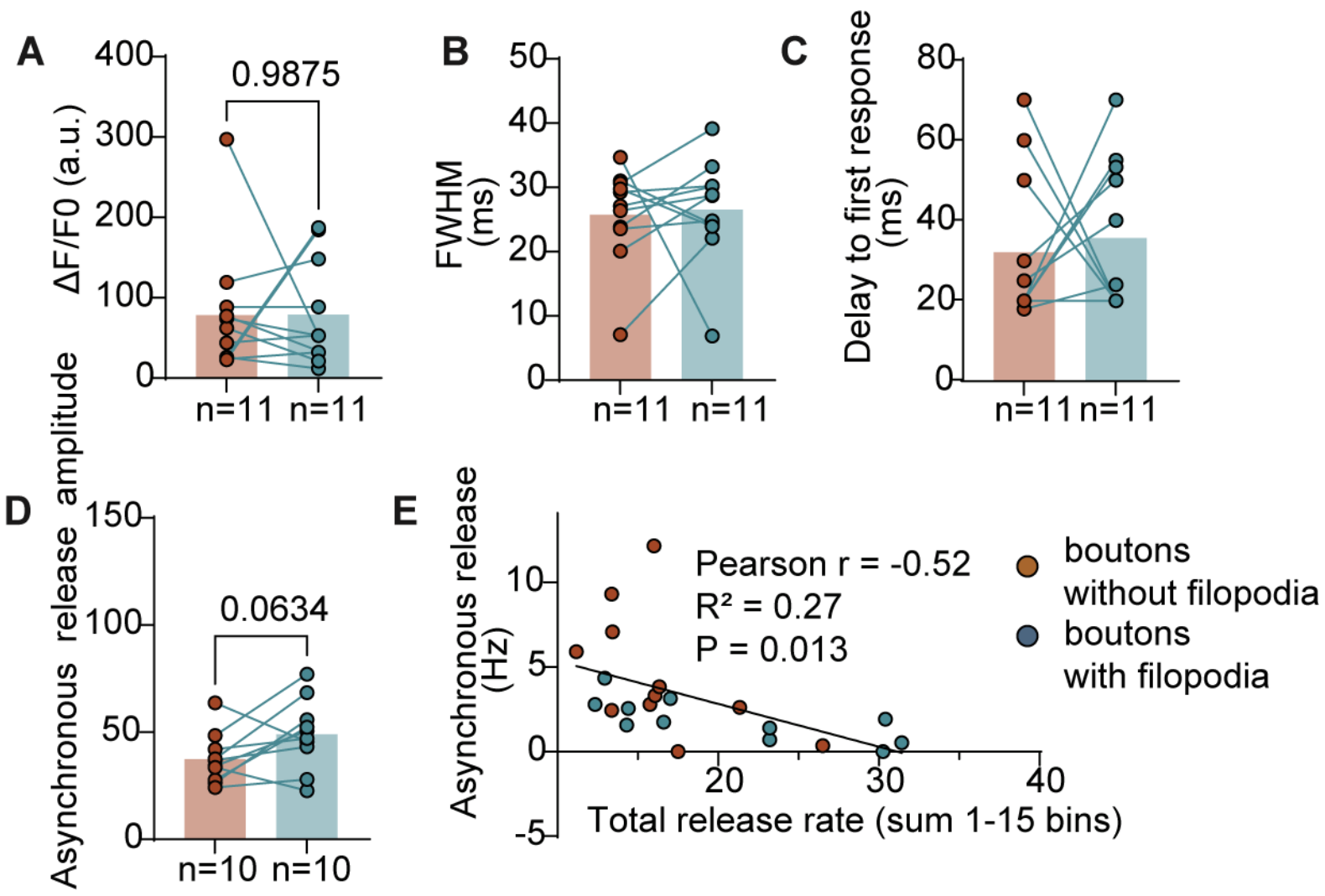

Fig. S7. Filopodia-forming and non-forming boutons show similar initial release properties and bouton-level covariation between synchronous and asynchronous release. (A to C) Parameters of initial iGluSnFR3.v857 release events at the onset of a 300-AP (20 Hz) stimulation train, compared between boutons with and without filopodia. **(A)** Peak response amplitude ($\Delta F/F_0$), **(B)** full width at half-maximum (FWHM, ms), and **(C)** delay to first response (ms). Bars are the mean. P values, paired t test. **(D)** Mean amplitude per cell of post-train asynchronous iGluSnFR3.v857 events at boutons with and without filopodia. Bars are the mean. P values, paired t test. **(E)** Correlation between post-train asynchronous release frequency (Hz) and cumulative synchronous release rate across the full stimulation train (sum of bins 1-15 s).

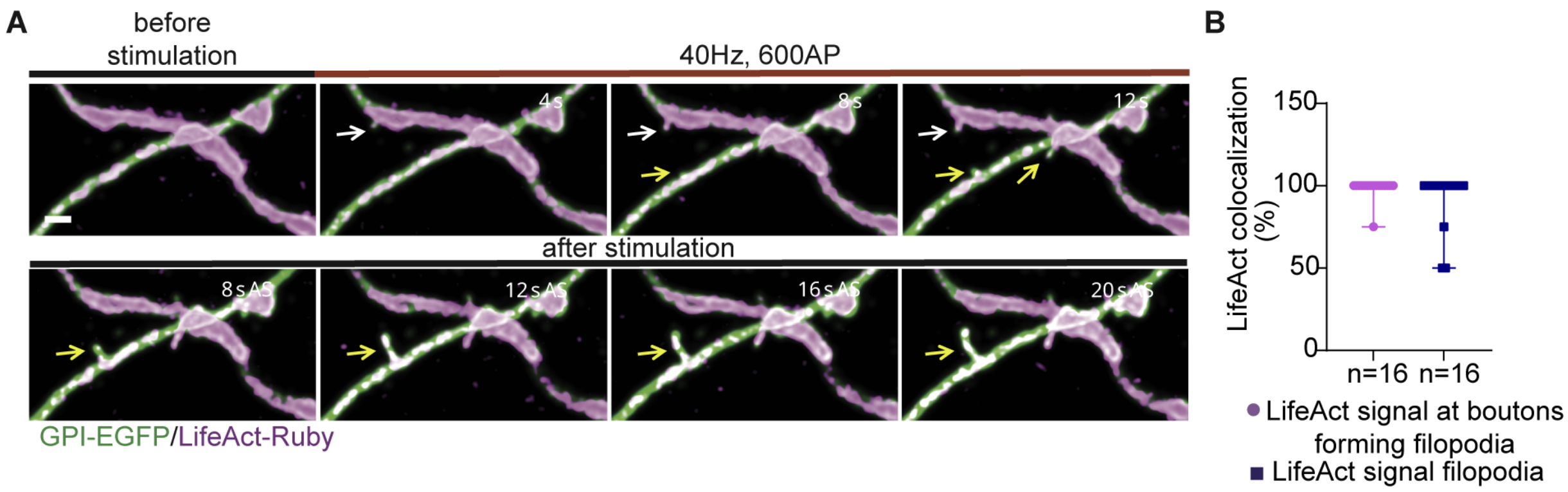

Fig. S8. Presynaptic filopodia contain filamentous actin. (A) Time-lapse series shows actin polymerization in emerging filopodia during 600 AP (40 Hz) stimulation in neurons co-transfected with GPI-EGFP and LifeAct-Ruby. White arrows, filopodia containing continuous actin filaments; yellow arrow, filopodia containing treadmilling actin filaments. Scale bar, 1 µm. **(B)** Percentages of filopodia-forming boutons and their filopodia displaying LifeAct-Ruby signal. Boxes are IQR, whiskers show Min to Max.

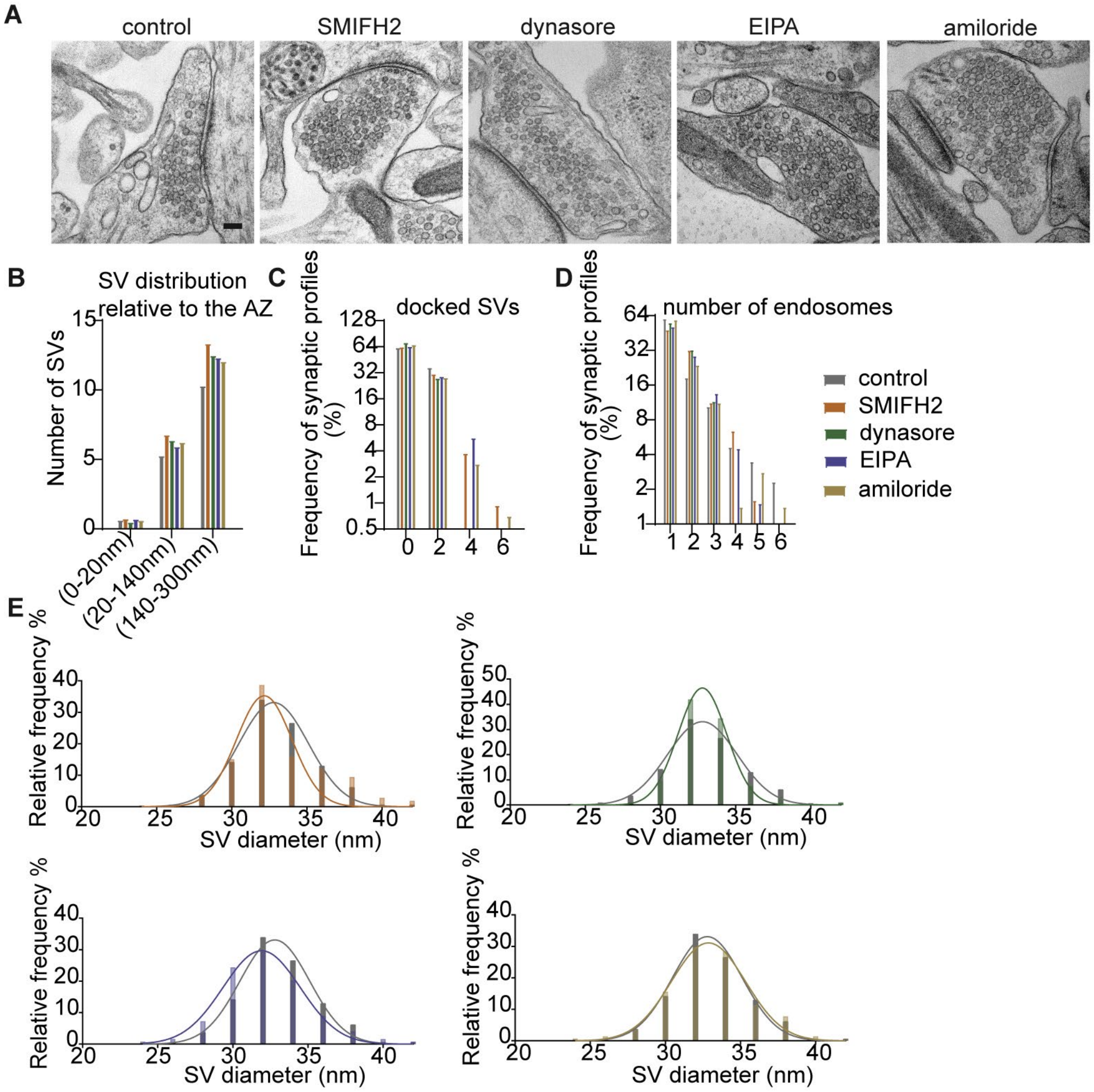

Fig. S9. Presynaptic ultrastructure following SMIFH2, EIPA, amiloride, or dynasore treatment. (A) Electron micrographs of synaptic boutons treated with SMIFH2, EIPA, amiloride, or dynasore and stimulated with 600 AP (40 Hz). Scale bar, 100 nm. **(B)** Number of SVs at increasing distances from the active zone (AZ). **(C)** Percentage of synaptic profiles containing different numbers of docked SVs. **(D)** Percentage of synaptic profiles containing different numbers of endosomes. **(E)** SV size distributions across treatment groups, with the control shown alongside each treatment for comparison.

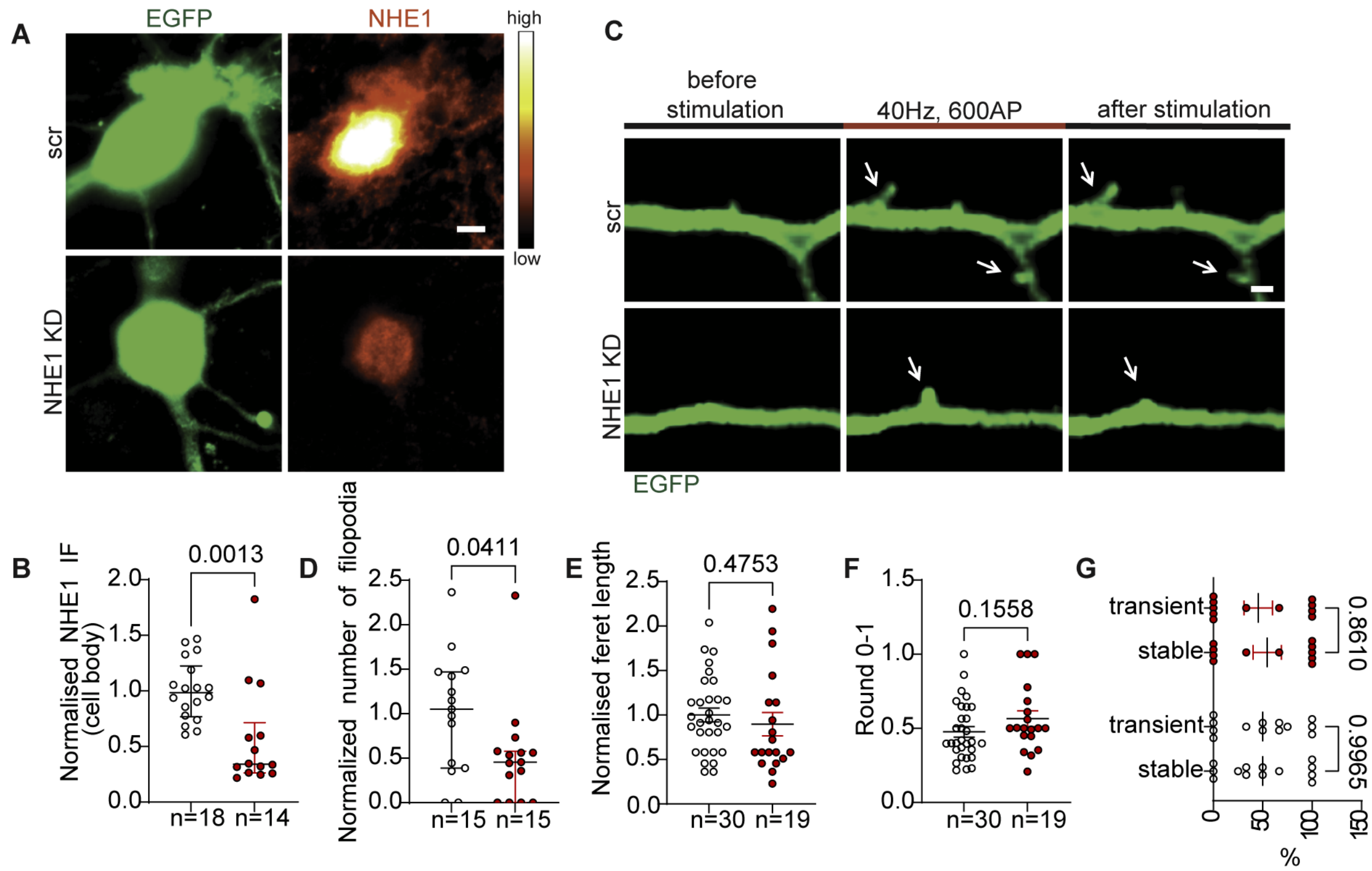

Fig. S10. NHE1 knockdown (KD) reduces presynaptic filopodia formation without affecting morphology or stability. (A to G) Neurons were transfected with NHE1-targeting or scrambled control shRNA, with EGFP as a reporter. **(A)** Images showing EGFP and NHE1 expression in neurons transfected with scrambled and NHE1KD plasmids. Scale bar, 5 µm. **(B)** Quantification of NHE1 expression in cell bodies. Lines are the median, error bars are IQR. P values, Mann-Whitney U test. **(C)** Time-lapse series showing filopodia formation in neurons transfected with scrambled or NHE1KD plasmids (white arrows, filopodia). Neurons were stimulated with 600 AP (40 Hz). Scale bar, 1 µm. **(D)** Normalized number of filopodia per 10 µm of axon length. Lines are the median, error bars are IQR. P values, Mann-Whitney U test. **(E)** Normalized filopodial length. Lines are the mean, error bars are SEM. P values, unpaired t test. **(F)** Quantification of filopodial shape. Lines are the mean, error bars are SEM. P values, unpaired t test. **(G)** Stable-to-transient filopodia ratio. Lines are the mean, error bars are SEM. P values, two-way ANOVA with Sidak's test.

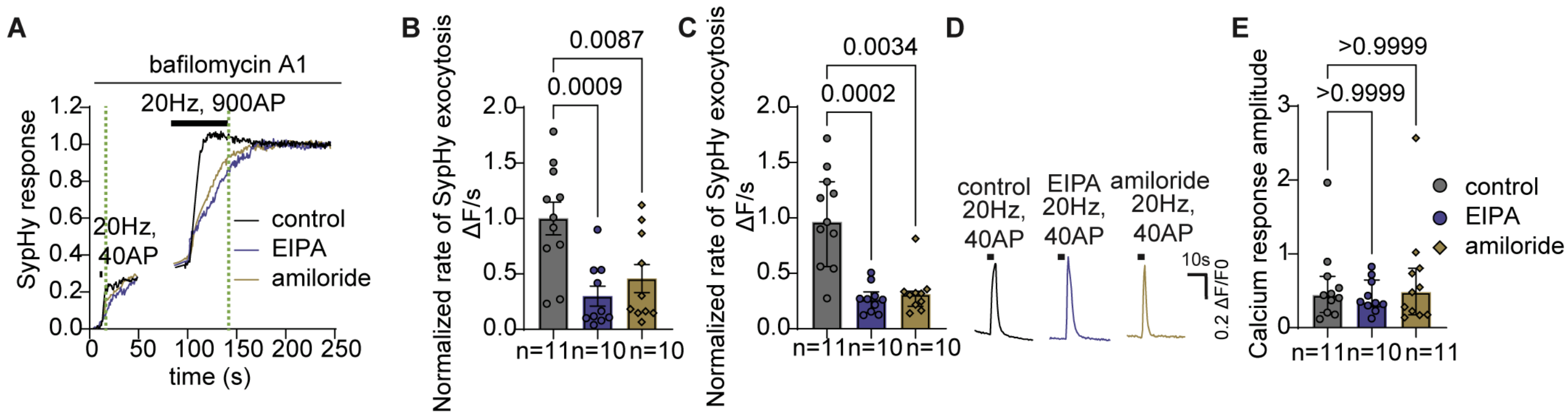

Fig. S11. NHE inhibitors delay SV exocytosis without altering stimulus-evoked calcium responses. (A to C) Hippocampal neurons expressing SypHy were treated with vehicle, EIPA, or amiloride for 30 min before live imaging and stimulated sequentially with 40- and 900-AP trains at 20 Hz in the presence of the respective treatments and 1 μM bafilomycin A1. **(A)** Mean SypHy response traces normalized to the amplitude of the second response. The dotted green line indicates the end of stimulation. **(B, C)** Normalized SypHy exocytosis rates following the first **(B)** and second **(C)** stimuli. **(B)** Bars are the mean, error bars are SEM. P values, one-way ANOVA with Dunnett's test; **(C)** Bars are the median, error bars are IQR. P values, Kruskal-Wallis with Dunn's test. **(D, E)** Neurons expressing GCaMP6f were treated with vehicle, EIPA, or amiloride for 30 min and stimulated with 40 AP at 20 Hz. **(D)** Representative GCaMP6f responses ($\Delta F/F_0$). **(E)** GCaMP6f response amplitudes. Bars are the median, error bars are IQR. P values, Kruskal-Wallis with Dunn's test.

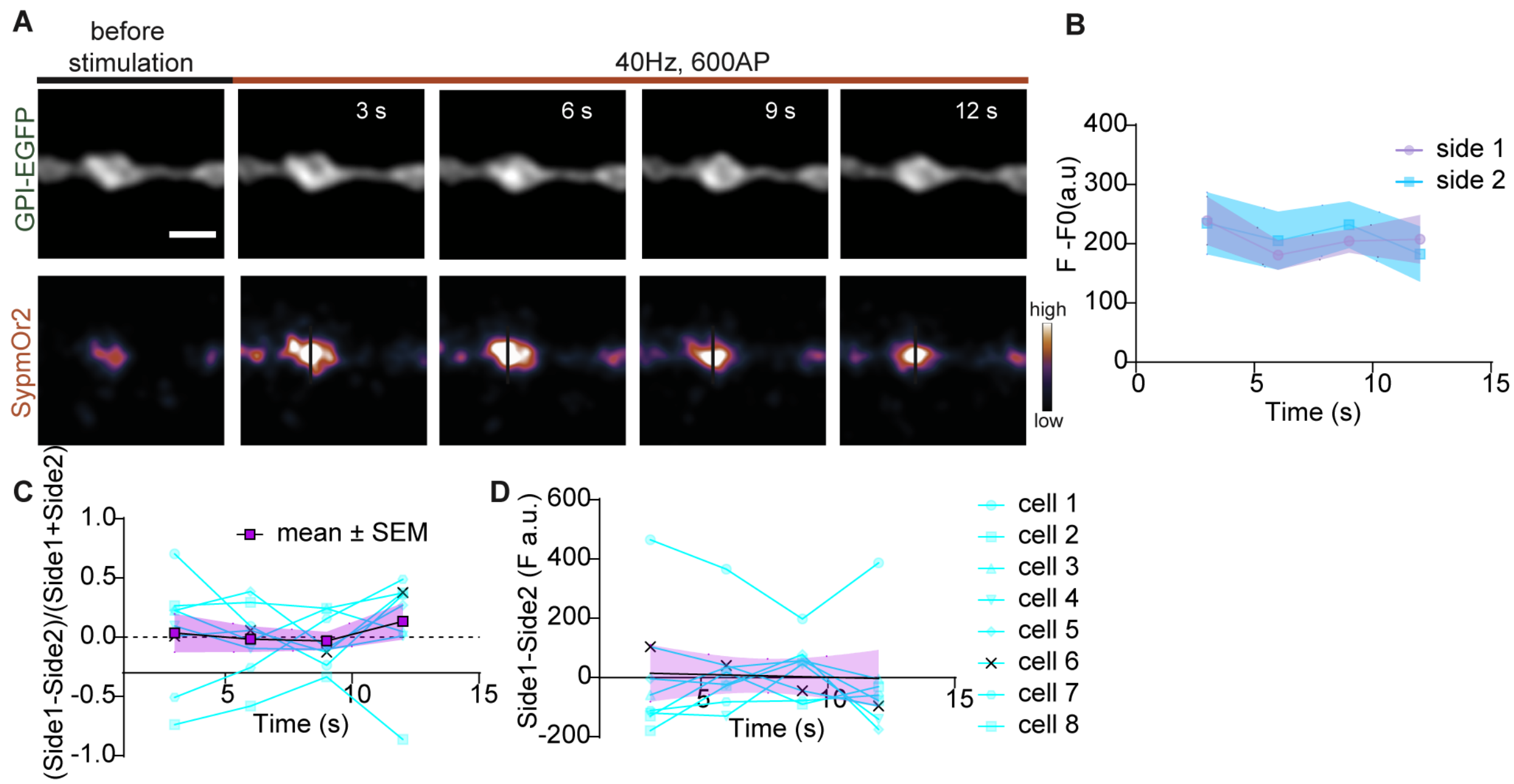

Fig. S12. Stimulus-induced directional redistribution of SypmOr2 is not detected in boutons without filopodia. **(A)** Time-lapse images of a hippocampal neuron co-expressing GPI-EGFP and SypmOr2 before and during field stimulation (600 AP at 40 Hz). Black bars separate sides 1 and 2 of the bouton. Scale bar, 1 µm. **(B)** SypmOr2 fluorescence at side 1 and side 2 ROIs during stimulation (mean ± SEM). P values, mixed-effects model with Sidak's test. **(C)** Normalized enrichment index over time for individual cells (blue lines) and mean ± SEM (filled symbols). P values, one-sample t test. **(D)** Pooled linear regression of flux over time. Cell traces, blue; 95% confidence interval, shading.

Table S3. Numerical values, sample sizes and statistical analysis for main figures.

Figure	mean ± SEM		median ± IQR	n – number of experiments (equivalent to coverslips or slices)	N – number of tissue culture preparations or animals	Statistical test used to calculate p values
Fig. 1B		mean ± SEM<br>43.78 ± 2.82		n =11	N = 4 cultures	N/A
Fig. 1C						
SypmOr2 responses at synapses forming filopodia		median<br>100		n =11	N = 4 cultures	N/A
		25th percentile<br>100				
		75th percentile<br>100				
SypmOr2 responses within filopodia		median<br>87.50				
		25th percentile<br>60.00				
		75th percentile<br>100				
Fig. 1I						
Segment 1 (0-200 nm)		median<br>0.00		n = 11	N = 3 samples from 3 cultures	Kruskal-Wallis with Dunn's multiple comparisons test
		25th percentile<br>0.00				
		75th percentile<br>7.14				
Segment 2 (200-400 nm)		median<br>20.40		n = 11 filopodia	N = 3 samples from 3 cultures	
		25th percentile<br>0.35				
		75th percentile<br>35.81				
Segment 3 (400-600 nm)		median<br>17.93		n = 11 filopodia	N = 3 samples from 3 cultures	
		25th percentile<br>0.00				
		75th percentile<br>48.72				
Segment 4 (600-800 nm)		median<br>10.15		n = 11 filopodia	N = 3 samples from 3 cultures	
		25th percentile<br>2.74				
		75th percentile<br>21.15				
Fig. 1J						

Segment 1 (0-200 nm)		median<br>0.00	n = 8 filopodia	N = 3 samples from 3 cultures	Kruskal-Wallis with Dunn's multiple comparisons test
		25th percentile<br>0.00			
		75th percentile<br>27.29			
Segment 2 (200-400 nm)		median<br>15.22	n = 8 filopodia	N = 3 samples from 3 cultures	
		25th percentile<br>6.73			
		75th percentile<br>52.56			
Segment 3 (400-600 nm)		median<br>20.41	n = 8 filopodia	N = 3 samples from 3 cultures	
		25th percentile<br>3.27			
		75th percentile<br>40.02			
Segment 4 (600-800 nm)		median<br>45.36	n = 5 filopodia	N = 3 samples from 3 cultures	
		25th percentile<br>0.00			
		75th percentile<br>75.77			
Fig. 2B					
Calcium responses		mean ± SEM<br>0.00 ± 0.00	n = 9	N = 4 cultures	N/A
Filopodia		mean ± SEM<br>0.00 ± 0.00	n = 9	N = 4 cultures	
Fig. 2F					
Mean DF/F0		mean ± SEM<br>42.33 ± 6.42	n = 17	N = 4 cultures	Paired t test
Max DF/F0 pre-T-max window		mean ± SEM<br>131.2 ± 29.29	n = 17	N = 4 cultures	
Fig. 2H					
Mean DF/F0		mean ± SEM<br>42.33 ± 6.42	n = 17	N = 4 cultures	Paired t test
DF/F0 at T-max		mean ± SEM<br>83.13 ± 20.70	n = 17	N = 4 cultures	
Fig. 2J					
Transient		mean ± SEM<br>40.92 ± 10.08	n = 17	N = 4 cultures	Unpaired t test
Stable		mean ± SEM<br>59.08 ± 10.08	n = 17	N = 4 cultures	
Fig. 2K					
5 min basal		mean ± SEM<br>1.00 ± 0.34	n = 7	N = 3 cultures	Mixed-effects model with Sidak's multiple
5 min 4-AP		mean ± SEM<br>3.22 ± 0.33	n = 7	N = 3 cultures	

5 min 4-AP + EGTA AM		mean ± SEM 0.23 ± 0.15	n = 9	N = 3 cultures	comparisons test
60 min basal		mean ± SEM 2.04 ± 0.79	n = 7	N = 3 cultures	Mixed-effects model with Sidak's multiple comparisons test
60 min 4-AP		mean ± SEM 6.46 ± 1.30	n = 7	N = 3 cultures	
60 min 4-AP + EGTA AM		mean ± SEM 0.27 ± 0.18	n = 9	N = 3 cultures	
Fig. 2N					
basal		median 0.00	n = 8 slices	N = 5 mice	Kruskal-Wallis with Dunn's multiple comparisons test
		25th percentile 0.00			
		75th percentile 0.00			
4-AP		median 1.00	n = 19 slices	N = 7 mice	
		25th percentile 0.00			
		75th percentile 1.00			
APV/CNQX		median 0.00	n = 14 slices	N = 7 mice	
		25th percentile 0.00			
		75th percentile 0.00			
Fig. 2O					
Transient		mean ± SEM 42.50 ± 14.93	n = 10 slices with filopodia (from 19 imaged)	N = 7 mice	Unpaired t test
Stable		mean ± SEM 57.50 ± 14.93	n = 10 slices with filopodia (from 19 imaged)	N = 7 mice	
Fig. 3D					
Bsn signal synapse		mean ± SEM 80.78 ± 6.24	n = 17	N = 3 cultures	N/A
Bsn signal filopodia		mean ± SEM 45.88 ± 7.87	n = 17	N = 3 cultures	
Fig. 3E					
vGAT signal synapse		mean ± SEM 30.26 ± 5.77	n = 36	N = 3 cultures	N/A
vGAT signal filopodia		mean ± SEM 29.50 ± 5.90	n = 36	N = 3 cultures	
Fig. 3F					
GluA signal synapse		mean ± SEM 59.34 ± 6.29	n = 33	N = 3 cultures	N/A

GluA signal filopodia		mean ± SEM<br>49.75 ± 6.66	n = 33	N = 3 cultures	
Fig. 3H					
before stimulation		mean ± SEM<br>7.16 ± 1.04	n = 19	N = 4 cultures	Paired t test
filopodia at maximum length		mean ± SEM<br>8.74 ± 1.04	n = 19	N = 4 cultures	
Fig. 3I					
before stimulation		mean ± SEM<br>51.09 ± 6.70	n = 19	N = 4 cultures	Paired t test
filopodia at maximum length		mean ± SEM<br>60.88 ± 5.64	n = 19	N = 4 cultures	
Fig. 4C					
Without filopodia		mean ± SEM<br>9.73 ± 1.20	n = 11	N = 3 cultures	Paired t test
With filopodia		mean ± SEM<br>9.42 ± 1.16	n = 11	N = 3 cultures	
Fig. 4D					
Without filopodia		mean ± SEM<br>1.58 ± 0.30	n = 11	N = 3 cultures	Paired t test
With filopodia		mean ± SEM<br>1.53 ± 0.20	n = 11	N = 3 cultures	
Fig. 4E					
Without filopodia	1	mean ± SEM<br>1.00 ± 0.00	n = 11	N = 3 cultures	Repeated measures ANOVA with Sidak's multiple comparisons test
	2	mean ± SEM<br>1.22 ± 0.14			
	3	mean ± SEM<br>1.36 ± 0.23			
	4	mean ± SEM<br>1.31 ± 0.18			
	5	mean ± SEM<br>1.18 ± 0.07			
	6	mean ± SEM<br>1.15 ± 0.06			
	7	mean ± SEM<br>1.20 ± 0.11			
	8	mean ± SEM<br>1.12 ± 0.13			
	9	mean ± SEM<br>1.05 ± 0.09			
	10	mean ± SEM<br>0.99 ± 0.09			
	11	mean ± SEM<br>1.17 ± 0.20			
	12	mean ± SEM<br>0.90 ± 0.10			
	13	mean ± SEM			

			1.02 ± 0.11			
	14		mean ± SEM<br>1.02 ± 0.12			
	15		mean ± SEM<br>0.75 ± 0.09			
With filopodia	1		mean ± SEM<br>1.00 ± 0.00	n = 11	N = 3 cultures	
	2		mean ± SEM<br>1.48 ± 0.17			
	3		mean ± SEM<br>1.33 ± 0.09			
	4		mean ± SEM<br>1.35 ± 0.11			
	5		mean ± SEM<br>1.61 ± 0.21			
	6		mean ± SEM<br>1.61 ± 0.22			
	7		mean ± SEM<br>1.34 ± 0.14			
	8		mean ± SEM<br>1.46 ± 0.22			
	9		mean ± SEM<br>1.39 ± 0.22			
	10		mean ± SEM<br>1.26 ± 0.15			
	11		mean ± SEM<br>1.32 ± 0.18			
	12		mean ± SEM<br>1.45 ± 0.24			
	13		mean ± SEM<br>1.44 ± 0.19			
	14		mean ± SEM<br>1.37 ± 0.21			
	15		mean ± SEM<br>1.15 ± 0.15			
Fig. 4F						
Without filopodia			mean ± SEM<br>0.92 ± 0.09	n = 11	N = 3 cultures	Paired t test
With filopodia			mean ± SEM<br>1.35 ± 0.19	n = 11	N = 3 cultures	
Fig. 4G						
Without filopodia			mean ± SEM<br>4.97 ± 1.15	n = 10	N = 3 cultures	Paired t test
With filopodia			mean ± SEM<br>2.06 ± 0.37	n = 10	N = 3 cultures	
Fig. 5B						
control			median<br>0.84	n = 11	N = 3 cultures	Kruskal-Wallis with Dunn's
			25th percentile			

		0.62			multiple comparisons test
		75th percentile 1.25			
SMIFH2		median 0.39	n = 14		
		25th percentile 0.00			
		75th percentile 0.68			
dynasore		median 0.55	n = 16		
		25th percentile 0.37			
		75th percentile 0.75			
EIPA		median 0.00	n = 15		
		25th percentile 0.00			
		75th percentile 0.10			
amiloride		median 0.00	n = 10		
		25th percentile 0.00			
		75th percentile 0.17			
Fig. 5C					
control		median 0.79	n = 43 filopodia	n = 11 cells (3 cultures)	Kruskal-Wallis with Dunn's multiple comparisons test
		25th percentile 0.60			
		75th percentile 1.20			
SMIFH2		median 0.56	n = 33 filopodia	n = 14 cells (3 cultures)	
		25th percentile 0.43			
		75th percentile 0.70			
dynasore		median 0.78	n = 52 filopodia	n = 16 cells (3 cultures)	
		25th percentile 0.65			
		75th percentile 1.06			
EIPA		median 0.58	n = 6 filopodia	n = 15 cells (3 cultures)	
		25th percentile			

		0.49			
		75th percentile 0.85			
amiloride		median 0.63	n = 4 filopodia	n = 10 cells (3 cultures)	
		25th percentile 0.43			
		75th percentile 0.90			
Fig. 5D					
control		median 0.50	n = 43 filopodia	n = 11 cells (3 cultures)	Kruskal-Wallis with Dunn's multiple comparisons test
		25th percentile 0.34			
		75th percentile 0.69			
SMIFH2		median 0.60	n = 33 filopodia	n = 14 cells (3 cultures)	
		25th percentile 0.47			
		75th percentile 0.72			
dynasore		median 0.49	n = 52 filopodia	n = 16 cells (3 cultures)	
		25th percentile 0.33			
		75th percentile 0.66			
EIPA		median 0.71	n = 6 filopodia	n = 15 cells (3 cultures)	
		25th percentile 0.44			
		75th percentile 0.76			
amiloride		median 0.63	n = 4 filopodia	n = 10 cells (3 cultures)	
		25th percentile 0.25			
		75th percentile 0.70			
Fig. 5E					
control	transient	mean ± SEM 35.4 ± 11.29	n = 11	N = 3 cultures	2way ANOVA with Sidak's multiple comparisons test
	stable	mean ± SEM 64.5 ± 11.29			
SMIFH2	transient	mean ± SEM 56.28 ± 10.21	n = 10		

	stable		mean ± SEM<br>43.72 ± 10.21			
dynasore	transient		mean ± SEM<br>35.17 ± 9.28	n = 16		
	stable		mean ± SEM<br>64.83 ± 9.28			
EIPA	transient		mean ± SEM<br>20.00 ± 20.20	n = 5		
	stable		mean ± SEM<br>80.00 ± 20.00			
amiloride	transient		mean ± SEM<br>50.00 ± 28.87	n = 3		
	stable		mean ± SEM<br>50.00 ± 28.87			
Fig. 5G						
			EPSC amplitude (pA)			
Control	100 µA		median<br>0.00	n = 14	N = 9 mice	2way ANOVA with Dunnett's multiple comparisons test
			25th percentile<br>0.00			
			75th percentile<br>38.48			
	1000 µA		median<br>100.7			
			25th percentile<br>40.34			
			75th percentile<br>279.5			
	10000 µA		median<br>322.7			
			25th percentile<br>216.8			
			75th percentile<br>605.7			
EIPA	100 µA		median<br>0.00	n = 13	N = 8 mice	
			25th percentile<br>0.00			
			75th percentile<br>46.71			
	1000 µA		median<br>109.5			
			25th percentile<br>0.00			
			75th percentile<br>301.3			
	10000 µA		median<br>452.7			
			25th percentile			

		180.2<br>75th percentile<br>1041			
Fig. 5K					
Control		mean ± SEM<br>3.23 ± 0.57	n = 12	N = 9 mice	Unpaired t test
EIPA		mean ± SEM<br>6.71 ± 0.70	n = 11	N = 8 mice	
Fig. 5L					
Control		mean ± SEM<br>-11.02 ± 1.46	n = 12	N = 9 mice	Unpaired t test
EIPA		mean ± SEM<br>-16.39 ± 2.67	n = 11	N = 8 mice	
Fig. 7B					
3 s		Distal_ mean ± SEM<br>234.3 ± 55.57	n = 7	N = 2 cultures	Mixed-effects model with Sidak's multiple comparisons test
		Filopodial_ mean ± SEM<br>302.9 ± 45.30	n = 7	N = 2 cultures	
6 s		Distal_ mean ± SEM<br>250.3 ± 47.02	n = 7	N = 2 cultures	
		Filopodial_ mean ± SEM<br>367.6 ± 38.34	n = 7	N = 2 cultures	
9 s		Distal_ mean ± SEM<br>157.5 ± 30.20	n = 7	N = 2 cultures	
		Filopodial_ mean ± SEM<br>421.6 ± 39.50	n = 7	N = 2 cultures	
12 s		Distal_ mean ± SEM<br>100.9 ± 32.04	n = 7	N = 2 cultures	
		Filopodial_ mean ± SEM<br>441.5 ± 28.19	n = 7	N = 2 cultures	
Fig. 7C					
3 s		mean ± SEM<br>0.20 ± 0.10	n = 7	N = 2 cultures	One sample t test
6 s		mean ± SEM<br>0.22 ± 0.10	n = 7	N = 2 cultures	
9 s		mean ± SEM<br>0.45 ± 0.09	n = 7	N = 2 cultures	
12 s		mean ± SEM<br>0.64 ± 0.11	n = 7	N = 2 cultures	
Fig. 7F					
Bin 1		mean ± SEM<br>10.83 ± 5.28	n = 7	N = 3 cultures	One sample t test
Bin 2		mean ± SEM<br>22.90 ± 7.63	n = 7	N = 3 cultures	
Bin 3		mean ± SEM<br>22.22 ± 9.22	n = 7	N = 3 cultures	

Bin 4		mean ± SEM 23.54 ± 11.52	n = 7	N = 3 cultures	
Bin 5		mean ± SEM 29.44 ± 6.66	n = 7	N = 3 cultures	
Bin 6		mean ± SEM 38.23 ± 10.63	n = 7	N = 3 cultures	
Bin 7		mean ± SEM 36.26 ± 5.81	n = 7	N = 3 cultures	
Bin 8		mean ± SEM 20.50 ± 8.08	n = 7	N = 3 cultures	
Bin 9		mean ± SEM 8.34 ± 4.08	n = 7	N = 3 cultures	
Bin 10		mean ± SEM 5.37 ± 3.72	n = 7	N = 3 cultures	
Fig. 7G					
Bin 1		mean ± SEM 25.74 ± 7.83	n = 7	N = 3 cultures	One sample t test
Bin 2		mean ± SEM 18.58 ± 5.56	n = 7	N = 3 cultures	
Bin 3		mean ± SEM 25.69 ± 6.18	n = 7	N = 3 cultures	
Bin 4		mean ± SEM 30.58 ± 7.18	n = 7	N = 3 cultures	
Bin 5		mean ± SEM 41.06 ± 10.34	n = 7	N = 3 cultures	
Bin 6		mean ± SEM 41.29 ± 6.51	n = 7	N = 3 cultures	
Bin 7		mean ± SEM 32.39 ± 8.12	n = 7	N = 3 cultures	
Bin 8		mean ± SEM 45.75 ± 9.29	n = 7	N = 3 cultures	
Bin 9		mean ± SEM 53.63 ± 11.86	n = 7	N = 3 cultures	
Bin 10		mean ± SEM 49.93 ± 10.16	n = 7	N = 3 cultures	
Fig. 7H					
Bin 1		mean ± SEM 14.91 ± 10.28	n = 7	N = 3 cultures	One sample t test
Bin 2		mean ± SEM -4.32 ± 4.60	n = 7	N = 3 cultures	
Bin 3		mean ± SEM 4.47 ± 10.57	n = 7	N = 3 cultures	
Bin 4		mean ± SEM 7.04 ± 14.41	n = 7	N = 3 cultures	
Bin 5		mean ± SEM 11.62 ± 5.95	n = 7	N = 3 cultures	
Bin 6		mean ± SEM 3.07 ± 10.37	n = 7	N = 3 cultures	
Bin 7		mean ± SEM	n = 7	N = 3	

		-3.88 ± 4.45		cultures	
Bin 8		mean ± SEM<br>25.26 ± 14.09	n = 7	N = 3<br>cultures	
Bin 9		mean ± SEM<br>45.29 ± 9.78	n = 7	N = 3<br>cultures	
Bin 10		mean ± SEM<br>44.56 ± 10.28	n = 7	N = 3<br>cultures	

Table S4. Numerical values, sample sizes and statistical analysis for supplementary figures.

Figure	mean ± SEM	median ± IQR	n – number of experiments (equivalent to coverslips or slices)	N – number of tissue culture preparations or animals	Statistical test used to calculate P values
Fig. S2D					
40Hz, 600AP	mean ± SEM 1.00 ± 0.17		n = 12	N = 3 cultures	one-way ANOVA with Tukey's multiple comparisons test
5Hz, 600AP	mean ± SEM 0.70 ± 0.09		n = 14		
20Hz, 40AP	mean ± SEM 0.56 ± 0.06		n = 15		
Fig. S2E					
40Hz, 600AP	median 0.93		n = 33 filopodia	N = 12 cells (3 cultures)	Kruskal-Wallis with Dunn's multiple comparisons test
	25th percentile 0.64				
	75th percentile 1.31				
5Hz, 600AP	median 0.54		n = 40 filopodia	N = 14 cells (3 cultures)	
	25th percentile 0.45				
	75th percentile 0.84				
20Hz, 40AP	median 0.89		n = 56 filopodia	N = 15 cells (3 cultures)	
	25th percentile 0.69				
	75th percentile 1.31				
Fig. S2F					
40Hz, 600AP	median 0.46		n = 33 filopodia	N = 12 cells (3 cultures)	Kruskal-Wallis with Dunn's multiple comparisons test
	25th percentile 0.31				
	75th percentile 0.67				
5Hz, 600AP	median 0.55		n = 40 filopodia	N = 14 cells (3 cultures)	
	25th percentile 0.36				
	75th percentile 0.73				
20Hz, 40AP	median 0.40		n = 56 filopodia	N = 15 cells (3 cultures)	
	25th percentile				

	0.33<br>75th percentile<br>0.59				
Fig. S2G					
40Hz, 600AP	stable mean ± SEM 53.19 ± 11.00	transient mean ± SEM 46.81 ± 11.00	n = 12	N = 3 cultures	2way ANOVA with Sidak's multiple comparisons test
5Hz, 600AP	stable mean ± SEM 59.10 ± 11.38	transient mean ± SEM 40.90 ± 11.38	n = 14	N = 3 cultures	
20Hz, 40AP	stable mean ± SEM 24.05 ± 6.45	transient mean ± SEM 75.95 ± 6.45	n = 15	N = 3 cultures	
Fig. S3A					
5 min basal	mean ± SEM 1.00 ± 0.07		n = 5 filopodia	N = 7 cells (3 cultures)	2way ANOVA with Sidak's multiple comparisons test
5 min 4-AP	mean ± SEM 1.07 ± 0.13		n = 27 filopodia	N = 7 cells (3 cultures)	
60 min basal	mean ± SEM 1.39 ± 0.28		n = 13 filopodia	N = 7 cells (3 cultures)	
60 min 4-AP	mean ± SEM 0.94 ± 0.07		n = 61 filopodia	N = 7 cells (3 cultures)	
Fig. S3B					
5 min basal	mean ± SEM 0.44 ± 0.04		n = 5 filopodia	N = 7 cells (3 cultures)	2way ANOVA with Sidak's multiple comparisons test
5 min 4-AP	mean ± SEM 0.40 ± 0.04		n = 27 filopodia	N = 7 cells (3 cultures)	
60 min basal	mean ± SEM 0.45 ± 0.06		n = 13 filopodia	N = 7 cells (3 cultures)	
60 min 4-AP	mean ± SEM 0.45 ± 0.03		n = 61 filopodia	N = 7 cells (3 cultures)	
Fig. S4B					
20Hz 40AP	median 1.00<br>25th percentile 1.00<br>75th percentile 1.00		n = 15	N = 3 cultures	Mann-Whitney test
Sucrose	median 0.00		n = 13	N = 3 cultures	

	25th percentile 0.00			
	75th percentile 0.00			
Fig. S4D				
Control	mean ± SEM 1.00 ± 0.01	N = 3	N = 3 samples	Unpaired t test
TeNT	mean ± SEM 0.44 ± 0.02	N = 3	N = 3 samples	
Fig. S4F				
control	median 0.84	n = 15	N = 3 cultures	Mann-Whitney test
	25th percentile 0.78			
	75th percentile 1.41			
TeNT	median 0.32	n = 15	N = 3 cultures	
	25th percentile 0.00			
	75th percentile 0.61			
Fig. S4G				
Control	median 0.91	n = 56 filopodia	N = 15 cells (3 cultures)	Mann-Whitney test
	25th percentile 0.63			
	75th percentile 1.02			
TeNT	median 0.43	n = 18 filopodia	N = 15 cells (3 cultures)	
	25th percentile 0.31			
	75th percentile 0.69			
Fig. S4H				
Control	median 0.47	n = 56 filopodia	N = 15 cells (3 cultures)	Mann-Whitney test
	25th percentile 0.32			
	75th percentile 0.64			
TeNT	median 0.62	n = 18 filopodia	N = 15 cells (3 cultures)	

	25th percentile<br>0.26			
	75th percentile<br>0.82			
Fig. S6A				
live	mean ± SEM<br>1.00 ± 0.16	n = 12	N = 3<br>cultures	Unpaired t test
fixed	mean ± SEM<br>0.45 ± 0.06	n = 18	N = 3<br>cultures	
Fig. S6B				
live	mean ± SEM<br>1.00 ± 0.08	n = 33<br>filopodia	N = 12 cells<br>(3 cultures)	Unpaired t test
fixed	mean ± SEM<br>0.93 ± 0.06	n = 44<br>filopodia	N = 18 cells<br>(3 cultures)	
Fig. S6C				
live	mean ± SEM<br>0.48 ± 0.04	n = 33<br>filopodia	N = 12 cells<br>(3 cultures)	Unpaired t test
fixed	mean ± SEM<br>0.50 ± 0.03	n = 44<br>filopodia	N = 18 cells<br>(3 cultures)	
Fig. S7A				
Without filopodia	mean ± SEM<br>78.20 ± 23.87	n = 11	N = 3<br>cultures	Paired t test
With filopodia	mean ± SEM<br>78.72 ± 19.51	n = 11	N = 3<br>cultures	
Fig. S7B				
Without filopodia	mean ± SEM<br>25.77 ± 2.24	n = 11	N = 3<br>cultures	Paired t test
With filopodia	mean ± SEM<br>26.58 ± 2.45	n = 11	N = 3<br>cultures	
Fig. S7C				
Without filopodia	mean ± SEM<br>35.67 ± 5.60	n = 11	N = 3<br>cultures	Paired t test
With filopodia	mean ± SEM<br>32.09 ± 5.65	n = 11	N = 3<br>cultures	
Fig. S7D				
Without filopodia	mean ± SEM<br>37.47 ± 3.72	n = 11	N = 3<br>cultures	Paired t test
With filopodia	mean ± SEM<br>49.05 ± 5.18	n = 11	N = 3<br>cultures	

Fig. S8B				
LifeAct signal bouton forming filopodia	mean ± SEM<br>98.44 ± 1.56	n = 16	N = 3 cultures	
LifeAct signal filopodia	mean ± SEM<br>92.19 ± 4.40	n = 16	N = 3 cultures	
Fig. S10B				
scr	median<br>0.98	n = 18	N = 3 cultures	Mann-Whitney test
	25th percentile<br>0.77			
	75th percentile<br>1.22			
NHE1 KD	median<br>0.34	n = 14	N = 3 cultures	
	25th percentile<br>0.26			
	75th percentile<br>0.72			
Fig. S10D				
scr	median<br>1.05	n = 15	N = 3 cultures	Mann-Whitney test
	25th percentile<br>0.39			
	75th percentile<br>1.47			
NHE1 KD	median<br>0.46	n = 15	N = 3 cultures	
	25th percentile<br>0.00			
	75th percentile<br>0.58			
Fig. S10E				
scr	mean ± SEM<br>1.00 ± 0.08	n = 30 filopodia	n = 15 cells (3 cultures)	Unpaired t test
NHE1 KD	mean ± SEM<br>0.90 ± 0.13	n = 19 filopodia	n = 15 cells (3 cultures)	
Fig. S10F				
scr	mean ± SEM<br>0.48 ± 0.04	n = 30 filopodia	n = 15 cells (3 cultures)	Unpaired t test
NHE1 KD	mean ± SEM<br>0.57 ± 0.05	n = 19 filopodia	n = 15 cells (3 cultures)	
Fig. S10G				
scr	transient: mean ± SEM<br>50.00 ± 11.02	n = 13	N = 3 cultures	2way ANOVA with Sidak's multiple comparisons test
	stable: mean ± SEM<br>50.00 ± 11.02			

NHE1 KD	transient: mean ± SEM 45.45 ± 14.41; stable: mean ± SEM 54.55 ± 14.41	n = 11	N = 3 cultures	
Fig. S11B				
control	mean ± SEM 1.00 ± 0.15	n = 11	N = 5 cultures	one-way ANOVA with Dunnett's multiple comparisons test
EIPA	mean ± SEM 0.30 ± 0.09	n = 10	N = 5 cultures	
amiloride	mean ± SEM 0.46 ± 0.13	n = 10	N = 5 cultures	
Fig. S11C				
control	median 0.96	n = 11	N = 5 cultures	Kruskal-Wallis with Dunn's multiple comparisons test
	25th percentile 0.56			
	75th percentile 1.33			
EIPA	median 0.27	n = 10	N = 5 cultures	
	25th percentile 0.15			
	75th percentile 0.33			
amiloride	median 0.31	n = 10	N = 5 cultures	
	25th percentile 0.20			
	75th percentile 0.34			
Fig. S11E				
control	median 0.44	n = 11	N = 3 cultures	Kruskal-Wallis with Dunn's multiple comparisons test
	25th percentile 0.21			
	75th percentile 0.70			
EIPA	median 0.34	n = 10		
	25th percentile 0.28			
	75th percentile 0.65			
amiloride	median 0.48	n = 11		
	25th percentile 0.18			
	75th percentile 0.81			

Fig. S12B				
3 s	Side1_ mean ± SEM<br>239.0 ± 40.77	n = 8	N = 2 cultures	Mixed-effects model with Sidak's multiple comparisons test
	Side2_ mean ± SEM<br>234.3 ± 52.45	n = 8	N = 2 cultures	
6 s	Side1_ mean ± SEM<br>181.0 ± 25.10	n = 8	N = 2 cultures	
	Side2_ mean ± SEM<br>205.2 ± 49.07	n = 8	N = 2 cultures	
9 s	Side1_ mean ± SEM<br>204.4 ± 19.72	n = 8	N = 2 cultures	
	Side2_ mean ± SEM<br>232.1 ± 39.39	n = 8	N = 2 cultures	
12 s	Side1_ mean ± SEM<br>207.5 ± 41.14	n = 8	N = 2 cultures	
	Side2_ mean ± SEM<br>182.5 ± 46.87	n = 8	N = 2 cultures	
Fig. S12C				
3 s	mean ± SEM<br>0.04 ± 0.16	n = 8	N = 2 cultures	One sample t test
6 s	mean ± SEM<br>-0.02 ± 0.11	n = 8	N = 2 cultures	
9 s	mean ± SEM<br>-0.03 ± 0.08	n = 8	N = 2 cultures	
12 s	mean ± SEM<br>0.13 ± 0.15	n = 8	N = 2 cultures	

Caption for Movie S1. Primary cultures of hippocampal neurons were co-transfected with GPI-EGFP and SypmOr2. Neurons were stimulated with 600 APs, 40 Hz. The movie shows time-lapse series of filopodia formation during stimulation at synapses that display concomitant SypmOr2 fluorescent responses. GPI-EGFP (gray scale) and SypmOr2 (pseudocolour) channels are shown side by side. The periods of stimulation and the time course of the experiment are indicated. Scale bar, 2 μm.

Caption for Movie S2. Primary cultures of hippocampal neurons were co-transfected with GPI-Apple (red) and GCaMP6f (green) and were incubated with 100 μM 4-AP for 30 min. Live imaging of transfected axons was performed for 5 min in the continuous presence of 4-AP. The movie illustrates the spatio-temporal coupling of filopodia formation and axonal calcium responses as reported by GCaMP6f. The time course of the experiment is indicated. Scale bar, 2 μm.

Captions for Movies S3 and S4. Simulated convection of membrane clutter over 600 ms.

S3. in the presence of filopodia (1000 nm/s)

S4. in the absence of filopodia (0 nm/s)

Captions for Movies S5 and S6. Simulated release probability at the active and peri-active zone over 600 ms.

S5. in the presence of filopodia (1000 nm/s)

S6. in the absence of filopodia (0 nm/s)

REFERENCES

1. M. Matsuzaki, N. Honkura, G. C. R. Ellis-Davies, H. Kasai, Structural basis of long-term potentiation in single dendritic spines. *Nature* **429**, 761–766 (2004).

2. J. Noguchi, M. Matsuzaki, G. C. Ellis-Davies, H. Kasai, Spine-neck geometry determines NMDA receptor-dependent Ca^{2+} signaling in dendrites. *Neuron* **46**, 609–622 (2005).

3. A. Destexhe, M. Rudolph, D. Paré, The high-conductance state of neocortical neurons in vivo. *Nat. Rev. Neurosci.* **4**, 739–751 (2003).

4. K. C. Bittner, A. D. Milstein, C. Grienberger, S. Romani, J. C. Magee, Behavioral time scale synaptic plasticity underlies CA1 place fields. *Science* **357**, 1033–1036 (2017).

5. W. Truccolo, J. A. Donoghue, L. R. Hochberg, E. N. Eskandar, J. R. Madsen, W. S. Anderson, E. N. Brown, E. Halgren, S. S. Cash, Single-neuron dynamics in human focal epilepsy. *Nat. Neurosci.* **14**, 635–641 (2011).

6. T. Xu, X. Yu, A. J. Perlik, W. F. Tobin, J. A. Zweig, K. Tennant, T. Jones, Y. Zuo, Rapid formation and selective stabilization of synapses for enduring motor memories. *Nature* **462**, 915–919 (2009).

7. A. Hayashi-Takagi, S. Yagishita, M. Nakamura, F. Shirai, Y. I. Wu, A. L. Loshbaugh, B. Kuhlman, K. M. Hahn, H. Kasai, Labelling and optical erasure of synaptic memory traces in the motor cortex. *Nature* **525**, 333–338 (2015).

8. S. O. Rizzoli, Synaptic vesicle recycling: Steps and principles. *EMBO J.* **33**, 788–822 (2014).

9. V. De Paola, S. Arber, P. Caroni, AMPA receptors regulate dynamic equilibrium of presynaptic terminals in mature hippocampal networks. *Nat. Neurosci.* **6**, 491–500 (2003).

10. W.-B. Gan, E. Kwon, G. P. Feng, J. R. Sanes, J. W. Lichtman, Synaptic dynamism measured over minutes to months: Age-dependent decline in an autonomic ganglion. *Nat. Neurosci.* **6**, 956–960 (2003).

11. N. Matsumoto, D. Barson, L. Liang, M. C. Crair, Hebbian instruction of axonal connectivity by endogenous correlated spontaneous activity. *Science* **385**, eadh7814 (2024).

12. T. Sutula, Seizure-induced axonal sprouting: Assessing connections between injury, local circuits, and epileptogenesis. *Epilepsy Curr.* **2**, 86–91 (2002).

13. H. De Belly, S. Yan, H. Borja da Rocha, S. Ichbiah, J. P. Town, P. J. Zager, D. C. Estrada, K. Meyer, H. Turlier, C. Bustamante, O. D. Weiner, Cell protrusions and contractions generate long-range membrane tension propagation. *Cell* **186**, 3049–3061.e15 (2023).

14. Q. Yan, C. Gomis Perez, E. Karatekin, Cell membrane tension gradients, membrane flows, and cellular processes. *Physiology (Bethesda)* **39** (2024), 231, 245.

15. A. R. Houk, A. Jilkine, C. O. Mejean, R. Boltyanskiy, E. R. Dufresne, S. B. Angenent, S. J. Altschuler, L. F. Wu, O. D. Weiner, Membrane tension maintains cell polarity by confining signals to the leading edge during neutrophil migration. *Cell* **148**, 175–188 (2012).

16. A. Diz-Muñoz, D. A. Fletcher, O. D. Weiner, Use the force: Membrane tension as an organizer of cell shape and motility. *Trends Cell Biol.* **23**, 47–53 (2013).

17. V. Gerganova, S. G. Martin, Going with the membrane flow: The impact of polarized secretion on bulk plasma membrane flows. *FEBS J.* **290**, 669–676 (2023).

18. V. Gerganova, I. Lamas, D. M. Rutkowski, A. Vjestica, D. G. Castro, V. Vincenzetti, D. Vavylonis, S. G. Martin, Cell patterning by secretion-induced plasma membrane flows. *Sci. Adv.* **7**, eabg6718 (2021).

19. T. H. Ogunmowo, H. Jing, S. Raychaudhuri, G. F. Kusick, Y. Imoto, S. Li, K. Itoh, Y. Ma, H. Jafri, M. B. Dalva, E. R. Chapman, T. Ha, S. Watanabe, J. Liu, Membrane compression by synaptic vesicle exocytosis triggers ultrafast endocytosis. *Nat. Commun.* **14**, 2888 (2023).

20. C. G. Perez, N. R. Dudzinski, M. Rouches, A. Landajuela, B. Machta, D. Zenisek, E. Karatekin, Rapid propagation of membrane tension at retinal bipolar neuron presynaptic terminals. *Sci. Adv.* **8**, eabl4411 (2022).

21. P. J. Wen, S. Grenklo, G. Arpino, X. Tan, H. S. Liao, J. Heureaux, S.-Y. Peng, H.-C. Chiang, E. Hamid, W.-D. Zhao, W. Shin, T. Näreoja, E. Evergren, Y. Jin, R. Karlsson, S. N. Ebert, A. Jin, A. P. Liu, O. Shupliakov, L.-G. Wu, Actin dynamics provides membrane tension to merge fusing vesicles into the plasma membrane. *Nat. Commun.* **7**, 12604 (2016).

22. I. Nikonenko, P. Jourdain, D. Muller, Presynaptic remodeling contributes to activity-dependent synaptogenesis. *J. Neurosci.* **23**, 8498–8505 (2003).

23. G. F. Kusick, M. Chin, S. Watanabe, Zap-and-freeze electron microscopy captures synaptic vesicle exocytosis with unprecedented temporal precision. *Microsc. Microanal.* **24**, 1254–1255 (2018).

24. C. F. Stevens, J. M. Sullivan, Regulation of the readily releasable vesicle pool by protein kinase C. *Neuron* **21**, 885–893 (1998).

25. P. J. Buckle, H. L. Haas, Enhancement of synaptic transmission by 4-aminopyridine in hippocampal slices of the rat. *J. Physiol.* **326**, 109–122 (1982).

26. C. Rosenmund, C. F. Stevens, Definition of the readily releasable pool of vesicles at hippocampal synapses. *Neuron* **16**, 1197–1207 (1996).

27. T. Söllner, S. W. Whiteheart, M. Brunner, H. Erdjument-Bromage, S. Geromanos, P. Tempst, J. E. Rothman, SNAP receptors implicated in vesicle targeting and fusion. *Nature* **362**, 318–324 (1993).

28. A. Morton, J. R. K. Marland, M. A. Cousin, Synaptic vesicle exocytosis and increased cytosolic calcium are both necessary but not sufficient for activity-dependent bulk endocytosis. *J. Neurochem.* **134**, 405–415 (2015).

29. M. L. Dustin, T-cell activation through immunological synapses and kinapses. *Immunol. Rev.* **221**, 77–89 (2008).

30. A. Shapson-Coe, M. Januszewski, D. R. Berger, A. Pope, Y. Wu, T. Blakely, R. L. Schalek, P. H. Li, S. Wang, J. Maitin-Shepard, N. Karlupia, S. Dorkenwald, E. Sjostedt, L. Leavitt, D. Lee,

J. Troidl, F. Collman, L. Bailey, A. Fitzmaurice, R. Kar, B. Field, H. Wu, J. Wagner-Carena, D. Aley, J. Lau, Z. Lin, D. Wei, H. Pfister, A. Peleg, V. Jain, J. W. Lichtman, A petavoxel fragment of human cerebral cortex reconstructed at nanoscale resolution. *Science* **384**, eadk4858 (2024).

31. A. Aggarwal, R. Liu, Y. Chen, A. J. Ralowicz, S. J. Bergerson, F. Tomaska, B. Mohar, T. L. Hanson, J. P. Hasseman, D. Reep, G. Tsegaye, P. Yao, X. Ji, M. Kloos, D. Walpita, R. Patel, M. A. Mohr, P. W. Tillberg, GENIE Project Team, L. L. Looger, J. S. Marvin, M. B. Hoppa, A. Konnerth, D. Kleinfeld, E. R. Schreiter, K. Podgorski, Glutamate indicators with improved activation kinetics and localization for imaging synaptic transmission. *Nat. Methods* **20**, 925–934 (2023).

32. P. S. Kaeser, W. G. Regehr, Molecular mechanisms for synchronous, asynchronous, and spontaneous neurotransmitter release. *Annu. Rev. Physiol.* **76**, 333–363 (2014).

33. D. J. Hagler Jr., Y. Goda, Properties of synchronous and asynchronous release during pulse train depression in cultured hippocampal neurons. *J. Neurophysiol.* **85**, 2324–2334 (2001).

34. E. Macia, M. Ehrlich, R. Massol, E. Boucrot, C. Brunner, T. Kirchhausen, Dynasore, a cell-permeable inhibitor of dynamin. *Dev. Cell* **10**, 839–850 (2006).

35. S. A. Rizvi, E. M. Neidt, J. Cui, Z. Feiger, C. T. Skau, M. L. Gardel, S. A. Kozmin, D. R. Kovar, Identification and characterization of a small molecule inhibitor of formin-mediated actin assembly. *Chem. Biol.* **16**, 1158–1168 (2009).

36. S. P. Denker, D. C. Huang, J. Orlowski, H. Furthmayr, D. L. Barber, Direct binding of the Na–H exchanger NHE1 to ERM proteins regulates the cortical cytoskeleton and cell shape independently of H^+ translocation. *Mol. Cell* **6**, 1425–1436 (2000).

37. W.-C. Sin, D. M. Moniz, M. A. Ozog, J. E. Tyler, M. Numata, J. Church, Regulation of early neurite morphogenesis by the Na^+/H^+ exchanger NHE1. *J. Neurosci.* **29**, 8946–8959 (2009).

38. A. Lagana, J. Vadnais, P. U. Le, T. N. Nguyen, R. Laprade, I. R. Nabi, J. Noël, Regulation of the formation of tumor cell pseudopodia by the Na^+/H^+ exchanger NHE1. *J. Cell Sci.* **113** (Pt 20), 3649–3662 (2000).

39. M. Koivusalo, C. Welch, H. Hayashi, C. C. Scott, M. Kim, T. Alexander, N. Touret, K. M. Hahn, S. Grinstein, Amiloride inhibits macropinocytosis by lowering submembranous pH and preventing Rac1 and Cdc42 signaling. *J. Cell Biol.* **188**, 547–563 (2010).

40. S. Sankaranarayanan, T. A. Ryan, Real-time measurements of vesicle-SNARE recycling in synapses of the central nervous system. *Nat. Cell Biol.* **2**, 197–204 (2000).

41. Y. Hua, A. Woehler, M. Kahms, V. Haucke, E. Neher, J. Klingauf, Blocking endocytosis enhances short-term synaptic depression under conditions of normal availability of vesicles. *Neuron* **80**, 343–349 (2013).

42. N. Gimber, G. Tadeus, T. Maritzen, J. Schmoranzer, V. Haucke, Diffusional spread and confinement of newly exocytosed synaptic vesicle proteins. *Nat. Commun.* **6**, 8392 (2015).

43. T. Schikorski, C. F. Stevens, Quantitative ultrastructural analysis of hippocampal excitatory synapses. *J. Neurosci.* **17**, 5858–5867 (1997).

44. C. B. Wit, P. R. Hiesinger, Neuronal filopodia: From stochastic dynamics to robustness of brain morphogenesis. *Semin. Cell Dev. Biol.* **133**, 10–19 (2023).

45. C. Lohmann, T. Bonhoeffer, A role for local calcium signaling in rapid synaptic partner selection by dendritic filopodia. *Neuron* **59**, 253–260 (2008).

46. F. R. Kiral, G. A. Linneweber, T. Mathejczyk, S. V. Georgiev, M. F. Wernet, B. A. Hassan, M. von Kleist, P. R. Hiesinger, Autophagy-dependent filopodial kinetics restrict synaptic partner choice during *Drosophila* brain wiring. *Nat. Commun.* **11**, 1325 (2020).

47. E. Sitarska, A. Diz-Muñoz, Pay attention to membrane tension: Mechanobiology of the cell surface. *Curr. Opin. Cell Biol.* **66**, 11–18 (2020).

48. H. De Belly, E. K. Paluch, K. J. Chalut, Interplay between mechanics and signalling in regulating cell fate. *Nat. Rev. Mol. Cell Biol.* **23**, 465–480 (2022).

49. A. Ghisleni, N. C. Gauthier, Mechanotransduction through membrane tension: It’s all about

propagation? *Curr. Opin. Cell Biol.* **86**, 102294 (2024).

50. A. S. Reddy, D. T. Warshaviak, M. Chachisvilis, Effect of membrane tension on the physical properties of DOPC lipid bilayer membrane. *Biochim. Biophys. Acta* **1818**, 2271–2281 (2012).

51. S. Boudkkazi, E. Carlier, N. Ankri, O. Caillard, P. Giraud, L. Fronzaroli-Molinieres, D. Debanne, Release-dependent variations in synaptic latency: A putative code for short- and long-term synaptic dynamics. *Neuron* **56**, 1048–1060 (2007).

52. P. R. F. Mendonca, E. Tagliatti, H. Langley, D. Kotzadimitriou, C. G. Zamora-Chimal, Y. Timofeeva, K. E. Volynski, Asynchronous glutamate release is enhanced in low release efficacy synapses and dispersed across the active zone. *Nat. Commun.* **13**, 3497 (2022).

53. A. G. Blankenship, M. B. Feller, Mechanisms underlying spontaneous patterned activity in developing neural circuits. *Nat. Rev. Neurosci.* **11**, 18–29 (2010).

54. L. A. Kirkby, G. S. Sack, A. Firl, M. B. Feller, A role for correlated spontaneous activity in the assembly of neural circuits. *Neuron* **80**, 1129–1144 (2013).

55. L. C. Katz, C. J. Shatz, Synaptic activity and the construction of cortical circuits. *Science* **274**, 1133–1138 (1996).

56. M. Munz, D. Gobert, A. Schohl, J. Poquerusse, K. Podgorski, P. Spratt, E. S. Ruthazer, Rapid Hebbian axonal remodeling mediated by visual stimulation. *Science* **344**, 904–909 (2014).

57. M. Anisimova, B. van Bommel, R. Wang, M. Mikhaylova, J. S. Wiegert, T. G. Oertner, C. E. Gee, Spike-timing-dependent plasticity rewards synchrony rather than causality. *Cereb. Cortex* **33**, 23–34 (2022).

58. N. G. Hedrick, W. J. Wright, T. Komiyama, Local and global predictors of synapse elimination during motor learning. *Sci. Adv.* **10**, eadk0540 (2024).

59. J. M. Rhee, M. K. Pirity, C. S. Lackan, J. Z. Long, G. Kondoh, J. Takeda, A. K. Hadjantonakis, In vivo imaging and differential localization of lipid-modified GFP-variant fusions in embryonic

stem cells and mice. *Genesis* **44**, 202–218 (2006).

60. V. Dunsing, A. Petrich, S. Chiantia, Multicolor fluorescence fluctuation spectroscopy in living cells via spectral detection. *eLife* **10**, e69687 (2021).

61. T.-W. Chen, T. J. Wardill, Y. Sun, S. R. Pulver, S. L. Renninger, A. Baohan, E. R. Schreiter, R. A. Kerr, M. B. Orger, V. Jayaraman, L. L. Looger, K. Svoboda, D. S. Kim, Ultrasensitive fluorescent proteins for imaging neuronal activity. *Nature* **499**, 295–300 (2013).

62. D. Ivanova, A. Dirks, C. Montenegro-Venegas, C. Schöne, W. D. Altrock, C. Marini, R. Frischknecht, D. Schanze, M. Zenker, E. D. Gundelfinger, A. Fejtova, Synaptic activity controls localization and function of CtBP1 via binding to Bassoon and Piccolo. *EMBO J.* **34**, 1056–1077 (2015).

63. J. Heck, P. Parutto, A. Ciuraszkiewicz, A. Bikbaev, R. Freund, J. Mitlöhner, M. Andres-Alonso, A. Fejtova, D. Holcman, M. Heine, Transient confinement of Ca_V2.1 Ca^{2+}-channel splice variants shapes synaptic short-term plasticity. *Neuron* **103**, 66–79.e12 (2019).

64. J. Schindelin, I. Arganda-Carreras, E. Frise, V. Kaynig, M. Longair, T. Pietzsch, S. Preibisch, C. Rueden, S. Saalfeld, B. Schmid, J. Y. Tinevez, D. J. White, V. Hartenstein, K. Eliceiri, P. Tomancak, A. Cardona, Fiji: An open-source platform for biological-image analysis. *Nat. Methods* **9**, 676–682 (2012).

65. J.-F. Gilles, M. Dos Santos, T. Boudier, S. Bolte, N. Heck, DiAna, an ImageJ tool for object-based 3D co-localization and distance analysis. *Methods* **115**, 55–64 (2017).

66. M. Weber-Boyvat, J. Kroll, T. Trimbuch, V. M. Olkkonen, C. Rosenmund, The lipid transporter ORP2 regulates synaptic neurotransmitter release via two distinct mechanisms. *Cell Rep.* **41**, 111882 (2022).

67. C. Schauer, T. Leinders-Zufall, Imaging calcium responses in GFP-tagged neurons of hypothalamic mouse brain slices. *J. Vis. Exp.*, e4213 (2012).

68. C. Butler, G. E. Saraceno, A. Kechkar, N. Bénac, V. Studer, J. P. Dupuis, L. Groc, R. Galland,

J.-B. Sibarita, Multi-dimensional spectral single molecule localization microscopy. *Front. Bioinform.* **2**, 813494 (2022).

69. D. F. Anderson, A modified next reaction method for simulating chemical systems with time dependent propensities and delays. *J. Chem. Phys.* **127**, 214107 (2007).

70. S. Takamori, M. Holt, K. Stenius, E. A. Lemke, M. Gronborg, D. Riedel, H. Urlaub, S. Schenck, B. Brügger, P. Ringler, S. A. Müller, B. Rammner, F. Gräter, J. S. Hub, B. L. De Groot, G. Mieskes, Y. Moriyama, J. Klingauf, H. Grubmüller, J. Heuser, F. Wieland, R. Jahn, Molecular anatomy of a trafficking organelle. *Cell* **127**, 831–846 (2006).

71. S. Watanabe, E. Boucrot, Fast and ultrafast endocytosis. *Curr. Opin. Cell Biol.* **47**, 64–71 (2017).

72. D. S. W. Lee, L. F. Oster, S. Son, D. A. Fletcher, Cell surface crowding is a tunable energetic barrier to cell-cell fusion. *Nat. Commun.* **16**, 7158 (2025).

73. G. Guigas, M. Weiss, Effects of protein crowding on membrane systems. *Biochim. Biophys. Acta* **1858**, 2441–2450 (2016).

74. M. Linden, P. Sens, R. Phillips, Entropic tension in crowded membranes. *PLoS Comput. Biol.* **8**, e1002431 (2012).

75. S. Reshetniak, J.-E. Ußling, E. Perego, B. Rammner, T. Schikorski, E. F. Fornasiero, S. Truckenbrodt, S. Köster, S. O. Rizzoli, A comparative analysis of the mobility of 45 proteins in the synaptic bouton. *EMBO J.* **39**, e104596 (2020).

76. J. Guo, J.-L. Ge, M. Hao, Z.-C. Sun, X.-S. Wu, J.-B. Zhu, W. Wang, P.-T. Yao, W. Lin, L. Xue, A three-pool model dissecting readily releasable pool replenishment at the calyx of held. *Sci. Rep.* **5**, 9517 (2015).

77. E. Hanse, B. Gustafsson, Vesicle release probability and pre-primed pool at glutamatergic synapses in area CA1 of the rat neonatal hippocampus. *J. Physiol.* **531**(Pt 2), 481–493 (2001).

78. L. E. Dobrunz, C. F. Stevens, Heterogeneity of release probability, facilitation, and depletion at central synapses. *Neuron* **18**, 995–1008 (1997).

79. C. F. Stevens, T. Tsujimoto, Estimates for the pool size of releasable quanta at a single central synapse and for the time required to refill the pool. *Proc. Natl. Acad. Sci. U.S.A.* **92**, 846–849 (1995).

80. S. Nadkarni, T. M. Bartol, T. J. Sejnowski, H. Levine, Modelling vesicular release at hippocampal synapses. *PLoS Comput. Biol.* **6**, e1000983 (2010).
